# Diversity and prevalence of type VI secretion system effectors in clinical *Pseudomonas aeruginosa* isolates

**DOI:** 10.3389/fmicb.2022.1042505

**Published:** 2023-01-04

**Authors:** Luca A. Robinson, Alice C. Z. Collins, Ronan A. Murphy, Jane C. Davies, Luke P. Allsopp

**Affiliations:** ^1^National Heart and Lung Institute, Imperial College London, London, United Kingdom; ^2^Department of Paediatric Respiratory Medicine, Royal Brompton Hospital, London, United Kingdom

**Keywords:** *Pseudomonas aeruginosa*, pangenome, T6SS, effectors, immunity, cystic fibrosis, mobile genetic elements

## Abstract

*Pseudomonas aeruginosa* is an opportunistic pathogen and a major driver of morbidity and mortality in people with Cystic Fibrosis (CF). The Type VI secretion system (T6SS) is a molecular nanomachine that translocates effectors across the bacterial membrane into target cells or the extracellular environment enabling intermicrobial interaction. *P. aeruginosa* encodes three T6SS clusters, the H1-, H2- and H3-T6SS, and numerous orphan islands. Genetic diversity of T6SS-associated effectors in *P. aeruginosa* has been noted in reference strains but has yet to be explored in clinical isolates. Here, we perform a comprehensive bioinformatic analysis of the pangenome and T6SS effector genes in 52 high-quality clinical *P. aeruginosa* genomes isolated from CF patients and housed in the Personalised Approach to *P. aeruginosa* strain repository. We confirm that the clinical CF isolate pangenome is open and principally made up of accessory and unique genes that may provide strain-specific advantages. We observed genetic variability in some effector/immunity encoding genes and show that several well-characterised *vgrG* and *PAAR* islands are absent from numerous isolates. Our analysis shows clear evidence of disruption to T6SS genomic loci through transposon, prophage, and mobile genetic element insertions. We identified an orphan *vgrG* island in *P. aeruginosa* strain PAK and five clinical isolates using *in silico* analysis which we denote *vgrG7*, predicting a gene within this cluster to encode a Tle2 lipase family effector. Close comparison of T6SS loci in clinical isolates compared to reference *P. aeruginosa* strain PAO1 revealed the presence of genes encoding eight new T6SS effectors with the following putative functions: cytidine deaminase, lipase, metallopeptidase, NADase, and pyocin. Finally, the prevalence of characterised and putative T6SS effectors were assessed in 532 publicly available *P. aeruginosa* genomes, which suggests the existence of accessory effectors. Our *in silico* study of the *P. aeruginosa* T6SS exposes a level of genetic diversity at T6SS genomic loci not seen to date within *P. aeruginosa,* particularly in CF isolates. As understanding the effector repertoire is key to identifying the targets of T6SSs and its efficacy, this comprehensive analysis provides a path for future experimental characterisation of these mediators of intermicrobial competition and host manipulation.

## Introduction

*Pseudomonas aeruginosa* is a gram-negative opportunistic pathogen that ubiquitously colonises the environment. The *P. aeruginosa* genome is larger (⁓5-7 Mbp) than other common gram-negative pathogens such as *Escherichia coli* (⁓3-4.7 Mbp) and has a mosaic composition (Stover et al., 2000). This diversity results in a large metabolic capacity and collection of virulence mechanisms that vary between strains, enabling *P. aeruginosa* to adapt to a broad range of niches (Moradali et al., 2017). *P. aeruginosa* is the dominant pathogen in individuals with Cystic Fibrosis (CF; an inherited genetic condition caused by mutations in the Cystic Fibrosis Transmembrane Conductance Regulator gene), particularly those in their teenage and adult years, and is therefore an agent of mortality and morbidity (Shteinberg et al., 2021). Infections of *P. aeruginosa* in CF patients can become chronic, forming biofilms and are difficult to eradicate (Maurice et al., 2018; Rossi et al., 2020). Several secreted virulence factors (e.g., Type III Secretion System) and surface structures (e.g., adhesins) that contribute to acute infection have been documented and the factors required for chronic infections (e.g., biofilm formation and quorum sensing) have remained elusive but are becoming better understood (Hauser, 2009; Lund-Palau et al., 2016; Jurado-Martín et al., 2021; Nolan and Allsopp, 2022). However, as virulence is multifactorial, and the diversity of clinical isolates is high, additional mechanisms that contribute to infection remain to be identified or characterised.

Bacterial secretion systems are a group of proteinaceous nanomachines, classified into Types I – XI, that enable the translocation of substrates across the bacterial membrane (Costa et al., 2015; Bhoite et al., 2019; Gorasia et al., 2020; Palmer et al., 2021; Filloux, 2022). The Type VI secretion system (T6SS) is a contractile transmembrane apparatus that delivers effector proteins and scavenging substrates into target cells or the surrounding milieu. Roles of this system include facilitating inter-microbial competition, host-pathogen manipulation, natural transformation, and nutrient acquisition (Matthey et al., 2019; Allsopp et al., 2020). The main body of the secretion apparatus is composed of three complexes: membrane associated (TssJLM), cytoplasmic baseplate (TssEFGK), and contractile sheath (TssBC; Wang et al., 2019). Further, hexameric stacks of Hcp proteins are encased by the sheath and topped by a spike complex consisting of a trimer of VgrG proteins and is sharpened by a PAAR protein. Contraction of the sheath propels the spike complex and any associated effectors across the bacterial membrane directly into the target cell, puncturing the recipient membrane (Wang et al., 2019). Recycling of the sheath components TssBC is then performed through depolymerisation of the sheath by the ATPase ClpV, allowing for additional rounds of assembly and firing (Zoued et al., 2014). Secreted effectors of the T6SS are classified according to their targets and functions. These effectors range in function with many found to possess anti-bacterial, anti-eukaryotic, and ion sequestering activity (Allsopp et al., 2020; Monjarás Feria and Valvano, 2020). Select effectors also exhibit trans-kingdom activity, affecting both prokaryotes and eukaryotes (Jiang et al., 2016). Many effectors are characterised as polymorphic, displaying modular composition with diversity in C-terminal toxicity (Zhang et al., 2012). To prevent self- or kin-intoxication, antibacterial effectors are generally encoded in tandem with neighbouring immunity genes which, when expressed, counteract the action of the toxic effectors (Coulthurst, 2019).

*Pseudomonas aeruginosa* encodes three evolutionarily distinct T6SS clusters (H1-, H2-, and H3-T6SS) within its large genome, with each harbouring the structural components, several effector-immunity (EI) pairs, and accessory proteins (Mougous et al., 2006; Allsopp et al., 2017; Wood et al., 2019b). Additional orphan EI pairs are also encoded distally from the central clusters within *vgrG* and *PAAR* islands (Allsopp et al., 2017) or solitarily, such as the EI pair *tse8*-*tsi8* (Nolan et al., 2021). The functions of these three T6SS clusters are some of the most studied T6SSs to date and exhibit antibacterial activity through the secretion of cognate effectors and anti-eukaryotic activity by the H2-and H3-T6SSs (Hood et al., 2010; Jiang et al., 2014). To date, the H1-T6SS has been shown to deliver nine characterised effectors that possess a range of cellular targets, including nucleic acids, cell membranes, NAD(P)^+^, and protein synthesis (Sana et al., 2016; Allsopp et al., 2020). Antibodies against Hcp1 in sera from CF patients suggest these systems are active during human infection (Mougous et al., 2006). Effectors belonging to the H2-and H3-T6SS clusters have been reported with lipase, metal ion sequestering, and trans-kingdom activities (Sana et al., 2015; Jiang et al., 2016; Berni et al., 2019; Wettstadt et al., 2019). Of particular note, the H2-T6SS has been demonstrated to promote internalisation into epithelial cells *in vitro*, via a VgrG2b-dependent mechanism, which may have consequences for human infection (Sana et al., 2012, 2015). The majority of effectors are dependent on cognate *vgrG*s for secretion which are usually encoded in close proximity (Hachani et al., 2014; Whitney et al., 2014). Interestingly, strain-specific effectors have been noted in reference strains PAO1, PAK and PA14, highlighting that genetic differences occur between genes encoded at the same chromosomal loci and exhibit discrete functions when expressed (Pissaridou et al., 2018; Ahmad et al., 2019). Further, specific effectors have also been shown to vary in prevalence within studied clinical isolates (Boulant et al., 2018). Thus, it appears *P. aeruginosa* isolates can harbour unique EI subsets that provide a mechanism to distinguish between “self” and “non-self” (Unterweger et al., 2012). Understanding the toxin repertoire of *P. aeruginosa* strains is therefore paramount as they may be used during infection to establish dominance over resident microbes or against competing strains. Thus, the T6SS could be acting as a direct virulence factor by targeting eukaryotic cells during infections in CF patients or indirectly through a role in interbacterial competition that could aid colonisation.

The bacterial pangenome is the complete collection of genes available to a species and is categorised into two distinct groups: ‘open’ or ‘closed’, depending on the propensity to acquire genetic material (Medini et al., 2005; Tettelin et al., 2005). Previous studies have reported that the *P. aeruginosa* species possesses an ‘open’ pangenome, where the core genome (genes present in all strains) contributes 90% of genes to an individual *P. aeruginosa* genome, but only 9% to the species’ complete pangenome (Ozer et al., 2014; Park et al., 2019). Generally, the accessory genome (genes present in a subset of strains) comprises significant proportions of the mobilome, virulome, and resistome (D’Auria et al., 2010). Many of these genes may not be necessary for overall species fitness and growth, but instead confer strain-specific advantages (Medini et al., 2005). As such, pangenome analysis possesses promising applications in clinical microbiology from vaccine design to identifying diagnostic and therapeutic targets (Bhardwaj and Somvanshi, 2017; Zeng et al., 2017; Anani et al., 2020) and understanding the microevolutionary dynamics of bacterial populations. Analyses of the *P. aeruginosa* accessory and unique (genes present in only one strain) genomes revealed the enrichment of mobilisable genetic material, including: phage, integrative and conjugative element, and plasmid genes, suggesting an important role in strain evolution (Ozer et al., 2014; Freschi et al., 2019). Dissemination of mobile elements, as well as virulence factors such as the T6SS, could lead to the development of core and accessory components, with the latter encoding specialised functions. This is further facilitated by toxin polymorphism, as is seen for T6SS effectors and the T5SS contact-dependent inhibition protein CdiA (Allen and Hauser, 2019), which result in toxin variants that have occurred after homologous recombination of genes encoding distinct C-terminal (CT) effector domains, varying in prevalence amongst strains (Ma et al., 2017).

**Figure 1:**
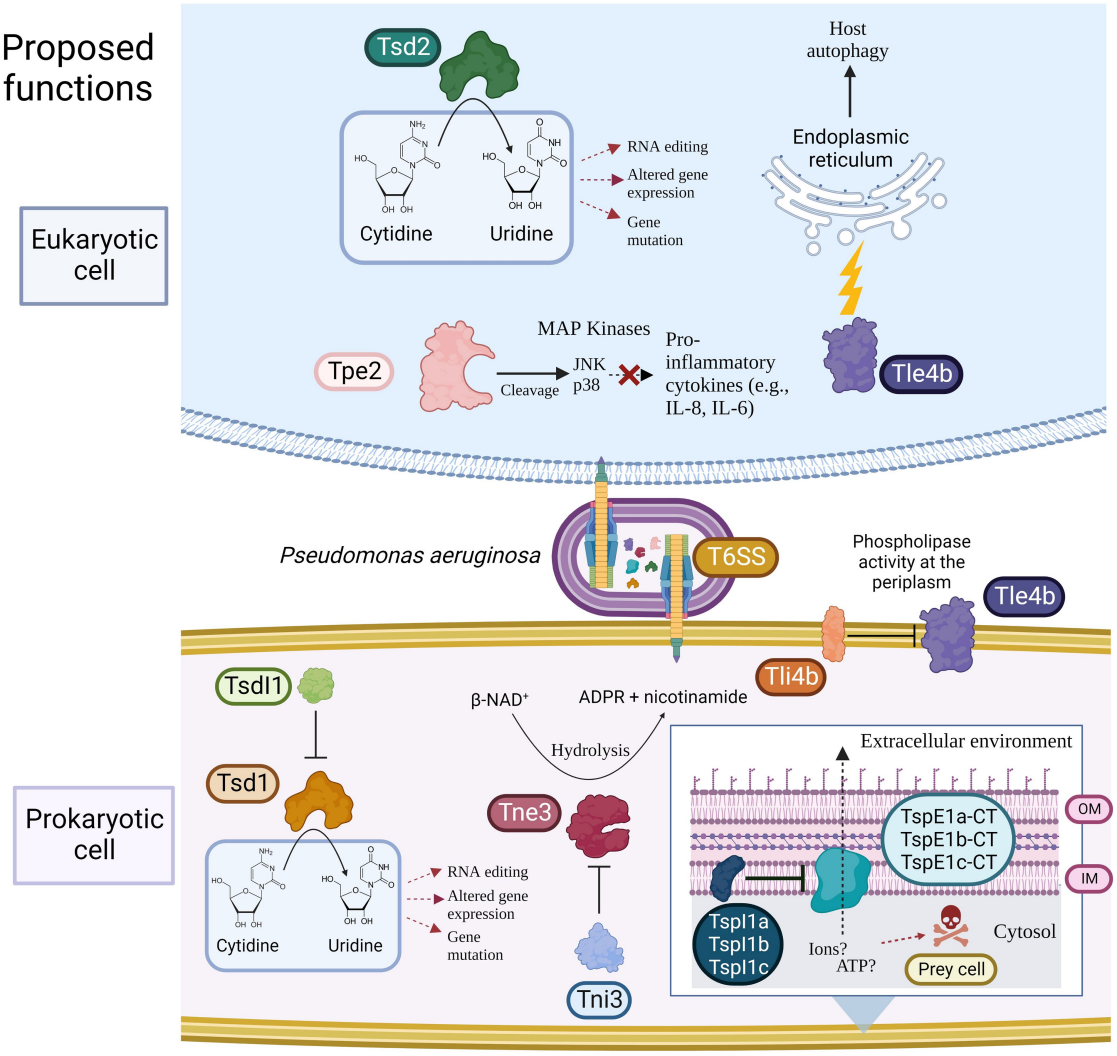
Model showing the predicted toxic functions of the eight putative T6SS effectors identified in this study towards either eukaryotic and/or prokaryotic cells. Putative immunity proteins are shown if present. T6SS = Type VI secretion system, Tsd = Type Six Cytidine Deaminase Effector, TsdI = Type Six Cytidine Deaminase Immunity, Tne/i = Type Six NADase Effector/Immunity, Tpe/i = Type Six Metallopeptidase Effector/Immunity, Tle/i = Type Six Lipase Effector/Immunity TspE/I = Type Six Pyocin Effector/Immunity, OM = Outer membrane, and IM = Inner membrane. Created using BioRender.com.

In this study, we bioinformatically investigate the *P. aeruginosa* pangenome and its T6SSs to uncover the prevalence and diversity of encoded effectors in 52 high-quality clinical *P. aeruginosa* genomes isolated from people with CF and stored in the Strategic Research Centre: Personalised Approach to *Pseudomonas aeruginosa* (SRC: PAPA) strain repository. Our data show that the investigated clinical pangenome is ‘open’ and the accessory genome is enriched for horizontal gene transfer (HGT) components and possesses an array of virulence factors and fitness-promoting genes. We observed significant diversity in the prevalence of effectors in both the 52 selected clinical isolates under study and 532 publicly available “complete” *P. aeruginosa* genomes from the NCBI RefSeq database. We used a detailed systematic comparative genomic approach, starting with all known T6SS-encoding genes and comparing these regions between strains. We particularly focused on those encoded in proximity to *vgrG*, *hcp* or *PAAR* encoding genes. Our analysis identified variants or novel genes encoding putative T6SS effectors and detailed protein function analysis revealed the presence of eight putative uncharacterised T6SS effectors predicted within the clinical isolates investigated and reference strains PAO1, PAK, and PA14. We putatively identify a Tle2 lipase effector within the *vgrG7* island of strain PAK, as well as bioinformatically define the putative function of eight new effector proteins (Figure 1). These analyses provide insight into inter-strain variability between *P. aeruginosa* isolates and the make-up of the population’s genes with regards to core and accessory clusters of the T6SS and effector diversity between strains.

## Materials and methods

### Strain samples and genome extraction of clinical *Pseudomonas aeruginosa* isolates

The 57 studied *P. aeruginosa* isolates had been cultured from samples of 49 patients with CF between February 2014 and February 2020: 44 individuals provided one isolate each, five provided two, and one subject provided three at different time points (Supplementary Table S1). Samples (sputum or cough swabs) had been processed by the Clinical Microbiology Laboratory at the Royal Brompton Hospital. Once diagnostic testing was complete, agar plates were provided to the Strategic Research Centre. Colonies were removed and stored on beads at -80°C in the SRC: PAPA strain repository. Isolates are coded for pseudonymisation; studies such as this, utilising no clinical data, do not require ethical approval or participant consent.

Single colonies of each strain were grown in broth. Cells were harvested and resuspended in a tube with DNA/RNA Shield (Zymo Research, United States) following Microbes NG strain submission procedures. Five to 40 μl of the suspension was lysed with 120 μl of TE buffer containing lysozyme (final concentration 0.1 mg/ml) and RNase A (ITW Reagents, Barcelona, Spain; final concentration 0.1 mg/ml) and incubated for 25 min at 37°C. Next, Proteinase K (VWR Chemicals, Ohio, United States; final concentration 0.1 mg/ml) and SDS (Sigma-Aldrich, Missouri, United States; final concentration 0.5% ^v^/_v_) were added and incubated for 5 min at 65°C. Genomic DNA was purified using an equal volume of SPRI beads and resuspended in EB buffer (Qiagen, Germany). DNA was quantified with the Quant-iT dsDNA HS kit (ThermoFisher Scientific) assay in an Eppendorf AF2200 plate reader (Eppendorf UK Ltd., United Kingdom).

### Genome sequencing, assembly and annotation

#### Illumina sequencing

Genomic DNA libraries were prepared using the Nextera XT Library Prep Kit (Illumina, San Diego, United States) following the manufacturer’s protocol with the following modifications: input DNA was increased 2-fold, and PCR elongation time was increased to 45 s. DNA quantification and library preparation were carried out on a Hamilton Microlab STAR automated liquid handling system (Hamilton Bonaduz AG, Switzerland). Pooled libraries were quantified using the Kapa Biosystems Library Quantification Kit for Illumina. Libraries were sequenced using Illumina sequencers (HiSeq/NovaSeq) using a 2 × 250 bp paired-end protocol by Microbes NG.^1^ Reads were adapter trimmed using Trimmomatic (v0.30) with a “sliding window” setting of Q15 (Bolger et al., 2014).

#### Illumina and Oxford Nanopore sequencing

Long read genomic DNA libraries were prepared with Oxford Nanopore SQK-RBK004 kit and/or SQK-LSK109 kit with Native Barcoding EXP-NBD104/114 (ONT, United Kingdom) using 400-500 ng of HMW DNA. Barcoded samples were pooled together into a single sequencing library and loaded in a FLO-MIN106 (R.9.4.1) flow cell in a GridION (ONT, United Kingdom). Short read Illumina reads were adapter trimmed using Trimmomatic (v0.30) with a “sliding window” setting of Q15 (Bolger et al., 2014). *De novo* hybrid genome assembly was performed using Unicycler (v0.4.0; Wick et al., 2017) or SPAdes (v3.7; Bankevich et al., 2012). The contigs were annotated using Prokka (v1.11; Seemann, 2014).

### Molecular sequence typing

Sequence types (ST) were confirmed against the PubMLST *P. aeruginosa* typing database (Jolley et al., 2018) for the 57 *P. aeruginosa* genomes and reference strains, using default parameters [last accessed November 2021].

### Average nucleotide identity of *Pseudomonas aeruginosa* isolates

*Pseudomonas aeruginosa* PAO1 (NC_002516.2), UCBPP-PA14 (NC_008463.1), and PAK (LR657304.1; Cain et al., 2019) genomes were downloaded from the NCBI GenBank database (Genome List - Genome - NCBI).^2^ Alignment-free average nucleotide identity (ANI) of sequenced *P. aeruginosa* clinical isolates and reference *P. aeruginosa* genomes, PAO1, PA14, and PAK, was calculated using FastANI (v1.32; Jain et al., 2018) with a fragment length of 3,000 bp and ‘many to many’ protocol. An ANI of >95-96% is considered the boundary for species delineation and represents a DNA–DNA hybridization value of >70% (Goris et al., 2007; Richter and Rosselló-Móra, 2009).

### Genomic and metadata data collection

Genome size (base pairs), G + C% content, number of coding DNA sequences (CDS) and tRNAs, N(50), and L(50) were collected using the sequencing data summary file, Artemis (v18.1.0; Carver et al., 2012), and the respective genome .gff file. Isolate metadata was supplied by the PAPA collection database. Genomes PALA31, PALA41, PALA46, PALA49, and PALA57 were removed from further downstream *in silico* analysis due to the possession of an N(50) below 6 Mbp, more than two contigs, and/or poor comparative alignment to reference *P. aeruginosa* strain PAO1. This left 52 *P. aeruginosa* genomes for the remainder of the analyses.

### Phylogenetic analysis of isolates by SNP calling

A phylogeny based on Single Nucleotide Polymorphism (SNP) calling and a concatenated alignment of the SNPs was inferred in the CSI Phylogeny (v1.4) webserver (Kaas et al., 2014), using default parameters and *P. aeruginosa* PAO1 as reference. This analysis involved the remaining 52 *P. aeruginosa* genomes and three reference genomes PAO1, PA14, and PAK. The Newick file generated by CSI Phylogeny was then visualised and annotated with ST using the iTOL (v6) webserver (Letunic and Bork, 2021).

### Pangenome analysis

To calculate the pangenome of the clinical isolates under study (n = 52), the genomes were analysed in Roary (v3.13.0; Page et al., 2015) using default parameters, except for core gene alignment specification which was defined using the options ‘-e --mafft’ (Katoh et al., 2002). Roary output files ‘number_of_genes_in_pan_genome.Rtab’ and ‘number_of_conserved_genes.Rtab’ were used to plot the power-law model curves in Microsoft Excel.

To describe whether the pangenome is ‘open’ or ‘closed’, Heaps’ law was applied to calculate the fitted constants κ and γ (Tettelin et al., 2008). Heap’s law is expressed by the formula:

*n* = κN^γ^

where *n* is the size of the pangenome, N is the total number of genomes, and κ and γ are fitting parameters. The exponent γ can be used to determine the open property of the pangenome by calculating α (α = 1 - γ). The pangenome is considered closed if α > 1 (γ < 0), and open if α < 1 (0 < γ < 1).

### Functional classification of orthologous (COG) cluster analysis

Annotation of core and accessory genes was achieved using eggNOG-mapper v5.0 (Huerta-Cepas et al., 2019). First, all gene identifiers from the Roary output file ‘pan_genome_reference.fa’ were extracted and copied into a .tsv file. Then, core and accessory gene names and identifiers were selected from the .tsv file using grep. Finally, FASTA sequences for the genes were extracted from the ‘pan_genome_reference.fa’ using seqtk.^3^ Gene nucleotide sequences were translated into amino acid sequences using the ‘Sequence Manipulation Suite’ (Stothard, 2000).^4^ EggNOG-mapper (v5.0; Huerta-Cepas et al., 2019) was then employed to annotate the protein sequences and assign COG categories using default parameters.

Statistical analysis was applied to determine the enrichment between pangenome groups (core and accessory genome) and COG category. Associations between the pangenome group and the abundance of each COG category’s orthologs were assessed using a two-tailed Chi-squared test (χ^2^). Analysis was performed in Microsoft’s Excel and significance determined on a value of *p* <0.0025 after Bonferroni correction (20 tests were conducted = 1 pangenome group × 20 COGs). The log odds ratio (LOR) was calculated based on the odds of the COG category occurring in the core relative to the odds of the category occurring in the accessory genome (LOR > 0 and LOR < 0 = positively or negatively associated with the core genome, respectively). Enrichment analysis was conducted if at least five genes in either the core or accessory genomes matched with a COG category. Those that possessed fewer than five were not investigated.

### Bioinformatic data collection

*Pseudomonas aeruginosa* T6SS components, effectors, immunity proteins, accessory proteins, and other secreted substrates from the literature were collated (Supplementary Table S2) for the identification of T6SSs amongst the sequenced clinical isolates. Nucleotide and amino acid sequences of the genes from reference *P. aeruginosa* strains PAO1 and PA14, well-characterised T6SS-expressing isolates (Mougous et al., 2006; Hachani et al., 2011; Allsopp et al., 2017), were downloaded from the NCBI RefSeq Genome and Protein databases [Genome List - Genome - NCBI (see footnote 2); Home - Protein - NCBI]^5^ and the *Pseudomonas* Genome Database^6^ (Winsor et al., 2016) in October 2021. Genes *PA0822, PA1659.1, PA2372, PA2684.1*, and *PA5086* were absent from the protein sequence file for *P. aeruginosa* PAO1 from the NCBI Protein Database (Home - Protein - NCBI) (see footnote 5), therefore their amino acid sequences were extracted from the *Pseudomonas* Genome Database (Winsor et al., 2016).

Nucleotide, amino acid, and annotated whole genome (.gbk and .gff file) sequences of 52 clinical *P. aeruginosa* genomes were used to create a local nucleotide and protein database using the respective files (Supplementary Table S1).

### *In silico* identification of *Pseudomonas aeruginosa* T6SS components

tBLASTn in BLAST+ (v2.12.0; Altschul et al., 1997; Camacho et al., 2009) was employed to screen the amino acid sequences of characterised T6SS components (Supplementary Table S2) against a local nucleotide dataset created for the *P. aeruginosa* genomes under study (*n* = 52) using an expected value (E-value) = 1e-10.

### Comparative genome visualisation of *Pseudomonas aeruginosa* clinical isolates to reference *Pseudomonas aeruginosa* genomes

Circular genome comparative visualisation was conducted using BLAST ring image generator (BRIG v0.95; Alikhan et al., 2011) with default parameters, except for ‘upper threshold’ and ‘lower threshold’ set as 80 and 50%, respectively. All 52 *P. aeruginosa* clinical isolates and reference strain PAO1, PA14, and PAK genomes were used in this analysis. *P. aeruginosa* PAO1 GenBank annotation file (NC_002516.2) was the reference. A tab-delimited file containing selected genome features of *P. aeruginosa* strain PAO1 and the location of the eight putative T6SS effectors identified in this study were set as the second-to-last and final ring, respectively.

For comparative analysis to *P. aeruginosa* strains PAO1, PA14, and PAK using Artemis Comparison Tool (v18.1.0; Carver et al., 2005), a crunch file was created using BLASTn in the BLAST+ command-line tool (v2.12.0; Altschul et al., 1990; Camacho et al., 2009) for each isolate genome.

To generate the gene cluster comparison figures, a perl script, ‘gb2fasta’^7^, was first used to trim the genome isolate and reference sequence .gbk files. Trimmed .gbk files were visualised using Clinker (v0.0.23; Gilchrist and Chooi, 2021), with default parameters.

### Protein function prediction

Webtools NCBI Conserved Domain Database (CDD; Marchler-Bauer et al., 2017) and Pfam (v35.0; El-Gebali et al., 2019) were used to identify protein domains. Signal peptides and transmembrane helices were predicted using SignalP-5.0 (Armenteros et al., 2019), TMHMM (v2.0; Sonnhammer et al., 1998) and Phobius (Käll et al., 2007) webservers. Subcellular localisation was predicted using the CELLO2GO webserver (Yu et al., 2014). Protein secondary structure prediction was calculated using PSIPred (UCL; Jones, 1999; Buchan and Jones, 2019) and JPred4 (Drozdetskiy et al., 2015). Protein sequences were aligned using ClustalOmega (ClustalO, v1.2.4, EBI; Madeira et al., 2022). Protein structure homology was predicted using the hidden Markov modelling (HMM) program HHpred (MPI Bioinformatics Toolkit; Zimmermann et al., 2018; Gabler et al., 2020). Protein sequence homologs were identified using NCBI Position-Specific Iterative (PSI)-BLAST against the RefSeq protein database (last accessed May 2022; Altschul et al., 1990, 1997). Amino acid sequence identity between two proteins was calculated with BLAST Global Alignment–Protein (Needleman-Wunsch Global Align; Altschul et al., 1990). Protein molecular weight (kDa) was predicted using the ExPASy ‘Compute pI/Mw’ tool^8^ (Bjellqvist et al., 1994; Wilkins et al., 1999; Gasteiger et al., 2005). Further protein function was also inferred using PROSITE (Sigrist et al., 2013). Default parameters were used throughout, with an E-value of 1e-2 as a cut-off and organism group defined as gram-negative where required. Structural homology modelling was performed using the Phyre2 server (Kelley et al., 2015) with parameter ‘intensive’, and the output PDB file was searched in the DALI protein structure comparison server against the Protein Data Bank (RCSB; Holm, 2020). Protein models were downloaded from Alphafold (Jumper et al., 2021; Varadi et al., 2022) and visualised using PyMOL (The PyMOL Molecular Graphics System, Version 2.5.4, Schrödinger, LLC). Homologs of protein sequences were searched by HMM using JackHMMER (EMBL-EBI, HmmerWeb v2.41.2; Potter et al., 2018) against the UniProtKB database with a maximum of five iterations, using an E-value of 1e-6 (last accessed May 2022).

To identify conserved residues in select proteins, a Position-Specific Scoring Matrix (PSSM) methodology was used similarly to that performed in Fridman et al. (Fridman et al., 2020). Briefly, a PSSM of the query protein was constructed using the full or partial amino acid sequence when referred. Five iterations of PSI-BLAST (Altschul et al., 1997) were performed against the NCBI RefSeq protein database with parameters for each iteration: a maximum of 500 hits and an E-value threshold of 1e-6 (last accessed May 2022). The PSSM was then filtered using an E-value of 1e-9. Five hundred identified homologs and their protein sequences were then downloaded from the NCBI RefSeq Protein database. Protein homolog amino acid sequences were aligned using MUSCLE (Edgar, 2004) in MEGAX (v10.2.6; Kumar et al., 2018) with default parameters. Identification of conserved residues was illustrated using the WebLogo3 server (v3.7.4; Crooks et al., 2004).

### Phylogenetic analysis of T6SS proteins

Amino acid sequences of investigated proteins were obtained from UniProtKB (Bateman et al., 2021) or NCBI Protein (Home - Protein - NCBI) (see footnote 5) databases and aligned using MUSCLE (Edgar, 2004). All phylogenetic trees were constructed using the Maximum-Likelihood method, Whelan and Goldman (WAG) modelling, Nearest-neighbour-interchange (NNI), and bootstrapping (*n* = 500) parameters. ‘Rates amongst sites’ and ‘Gaps/missing data treatment’ were the only parameters to vary between phylogenetic tree constructions and was either set as Gamma distributed (G) or Gamma distributed with invariant sites (G + I; *n* = 5), or “partial deletion (95%)” or “use all sites,” respectively. All analyses were conducted in MEGAX (Kumar et al., 2018).

### *In silico* identification of T6SS effectors within NCBI *Pseudomonas aeruginosa* genomes

Nucleotide sequences of *P. aeruginosa* genomes were collected from the NCBI RefSeq genome database release 212 (May 2022) at assembly level “complete” or higher (Genome List - Genome - NCBI) (see footnote 2) and a local nucleotide database was constructed. This local dataset population contained 532 genomes (Supplementary Table S3).

tBLASTn (Altschul et al., 1990) was employed to identify the presence and genomic context of characterised and putative T6SS effectors in the *P. aeruginosa* genome database. The amino acid sequences of T6SS effectors were aligned against the local nucleotide dataset created for the *P. aeruginosa* genomes.

## Results and discussion

### Sequencing and phylogenetic analysis reveals two distinct clades of CF *Pseudomonas aeruginosa* strains in our study

To investigate the diversity and prevalence of the T6SS in *P. aeruginosa* isolates from people with CF, we first sequenced 57 strains to generate high-quality genomes through sequencing with Illumina short-read and Oxford Nanopore long-read technologies (Supplementary Table S1). We then compared each genome to every other genome in the population under study using an average nucleotide identity (ANI) calculation, as well as to reference strains PAO1, PA14, and PAK (Supplementary Figure S1). The resulting ANI values were distributed between 97.24 and 100.00%, highlighting intraspecies variation, but all within the species delineation boundary (>95-96%). To minimise the influence of genomic outliers, we performed a quality control step to ensure all genomes possessed similar assembly attributes and genome characteristics (see Methods). This resulted in the removal of PALA31, PALA41, PALA46, PALA49 and PALA57, leaving 52 genomes that assembled into two or fewer contigs (Supplementary Table S1). The high-quality nature of these genomes made the subsequent analysis more accurate and minimised the effect of repetitive or highly homologous genes, such as *vgrG* genes, confounding the results.

**Figure 2:**
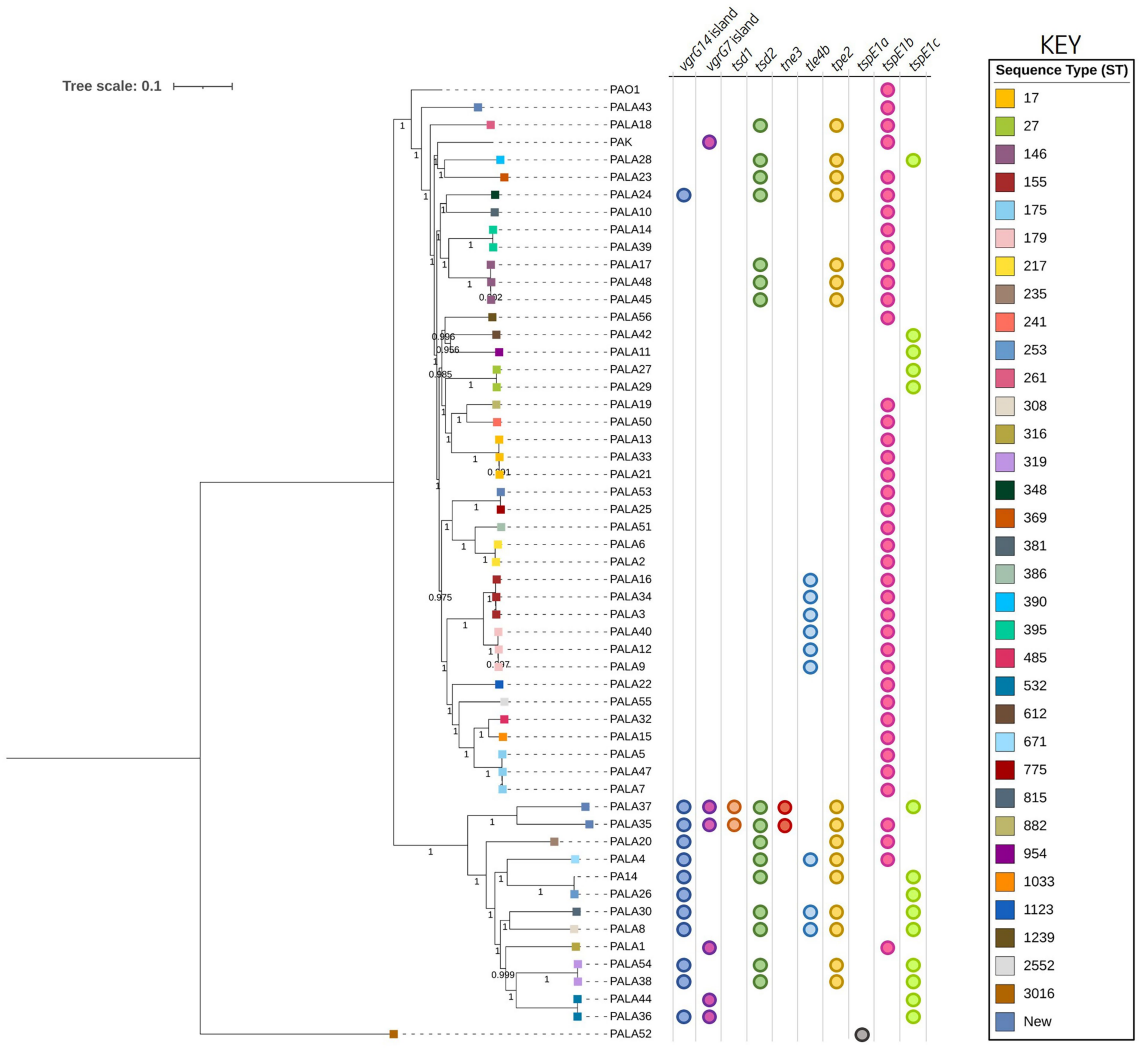
Phylogenetic tree highlighting the diversity of clinical *P. aeruginosa* isolates under study and distribution of effector genes in the accessory genome. The tree was constructed using the clinical isolate genomes and reference *P. aeruginosa* strains PAO1, PA14, and PAK. SNP analysis was inferred using CSI Phylogeny with PAO1 as a reference and visualised using iTOL. Sequence type (ST) is annotated on the isolate node with squares coloured according to the key. The scale bar indicates the genetic distance and bootstrap values are indicated. Circles indicate the presence of genes encoding putative effectors/*vgrG* islands in the respective genomes.

A phylogenetic analysis was then conducted to assess the genetic relationship between clinical isolates and reference strains PAO1, PA14, and PAK. This analysis showed that the *P. aeruginosa* isolates grouped most commonly according to their sequence type (ST) with our strains belonging to 34 STs, four of which were new, showing the diversity of sequenced strains. This analysis also highlighted that potentially related clones belonging to the same ST were present within our population (Figure 2). Two distinct clades could be discerned, with one possessing 12 isolates grouping with PA14 and the second possessing 39 isolates clustering most closely with PAO1 and PAK. Isolate PALA52 was the only outlier and possessed significant nucleotide sequence divergence. As expected, the grouping of most isolates into two distinct clades with reference strains PAO1/PAK or PA14 suggests a shared common ancestor; however, the diversity present within the population of *P. aeruginosa* isolates highlights the importance of investigating clinical strains.

### Clinical *Pseudomonas aeruginosa* isolates possess an open pangenome and a large accessory component

To explore the genetic makeup and distribution of gene functions in the *P. aeruginosa* clinical CF isolates, we then performed pangenome analysis using Roary (Page et al., 2015). We detected 16,195 orthologs across the 52 isolate pangenome. The relaxed core genome (present in >95% of isolates) consisted of 5,145 genes (32% of the pangenome; Supplementary Figure S2A) and is similar in size to core genomes from previous studies (Subedi et al., 2018). Genes in this core genome may represent those essential for the survival of *P. aeruginosa*. The largest proportion of the pangenome was the accessory genome (present in >0 and < 95% of strains) and contained 11,050 genes (68%). The contribution of the accessory to the complete genome of each isolate ranged from 8.59 – 20.99% with an average number of 961 (15.57%, 485 – 1,368) genes (Supplementary Figure S2B). Within the accessory genome, 5,178 singletons (genes found in one isolate) were identified (avg. 99.6, 0 – 374). We used Heap’s law to calculate whether the pangenome of our *P. aeruginosa* isolates is ‘open’ or ‘closed’ (Tettelin et al., 2008). Exponent α was calculated as 0.702 ± 0.065 indicating an ‘open’ pangenome (γ = 0.2979), supported by the increase in pangenome size and decrease in core genome size observed after each genome addition (Supplementary Figures S3A,B). A study by Gabrielaite et al. exploring the intra-lineage and pangenome of 45 *P. aeruginosa* lineages (474 isolates) from 34 CF patients determined 4,887 core, 9,575 accessory, and 4,932 unique genes (Gabrielaite et al., 2020). This is comparable to our results (5,145) and suggests a core genome of ~5,000 genes is required for CF *P. aeruginosa* isolates. Overall, these data suggest *P. aeruginosa* isolates from people with CF are diverse and as a population possess a pangenome that is amenable to genetic exchange and expansion (Klockgether et al., 2011).

After determining the clinical *P. aeruginosa* pangenome is open, we next investigated the genetic composition of both the core and accessory genomes and compared the enrichment of gene functions. We performed Cluster of Orthologous Groups (COG) analysis on the core and accessory genomes to assign gene family functions (Supplementary Figure S3C). Approximately, 98% of the core genes matched the eggNOG-mapper database, whereas 21% of the accessory genome had no match. The core genome was significantly enriched for COG families encoding housekeeping and metabolic functions, whilst the accessory genome was enriched for DNA replication/recombination/repair, intracellular secretion, and defence families (Supplementary Table S4). Strikingly, our core genome possessed only five genes identified as ‘transposase’ and two as ‘integrase’. In contrast, our accessory genome possessed 455 ‘transposase’ and 263 ‘integrase’ genes. This result shows the diversity of HGT elements present in our strains and suggests that HGT may be driving genetic diversification increasing the accessory genome. This is in line with previous work showing that *P. aeruginosa*’s regions of genome plasticity and mobile genetic elements (MGE) are key components of its accessory genome, harbouring antimicrobial resistance genes, virulence factors, metabolism genes, transcriptional regulators, and other survival genes (Mathee et al., 2008; Kung et al., 2010; Klockgether et al., 2011). Genes of unknown function (S) contributed 19.28 and 22.99% to the core and accessory genomes, respectively (Supplementary Table S4). Inversely, genes with a metabolic function (COG families E-I, P and Q) contributed 35.80 and 11.89% to the core and accessory genomes, respectively. This was an expected result as genes required for metabolism are far more likely to be essential for key cellular processes and metabolic pathways required for bacterial growth. On the other hand, as the accessory genome is more variable and tends to contain more MGE or genes for conditional survival within particular niches, such as virulence factors not present in all strains, more variability is anticipated.

Following investigation of gene family differences between the clinical *P. aeruginosa* core and accessory genomes, we next surveyed the presence of several fitness-promoting factors in the accessory genome, including secretion systems, that may highlight strain-specific functions. Notably, the accessory genome of the *P. aeruginosa* isolates under study possessed several significant survival and fitness-promoting factors, including components of Type I (*symE, ptaRNA1*, and *ydaTS*), II (*parDE, hicAB, prlF/yhaV, mazE, phd, yefM/yoeB*, and *mqsA*), and IV (*abiEii*) toxin-antitoxin systems. We also observed differences in components of the Type II (*pilC, pilB, pilR, gspI, xcsE, gspK, gspH, exeA*, and *pilQ*), IV (*trbD, trbB, virB11, virB4/trbE, virD2, virD4/traG, traF, trbF*, and *virB3*-like), and V (DUF637, PT-VENN, and POTRA domain-containing proteins) secretion systems. Numerous resistance factors (e.g., drug transporters, efflux pumps, antimicrobial resistance genes, copper, arsenic, mercury, tellurium, and cadmium resistance genes) were also present. Of particular note the *P. aeruginosa* Type VI secretion system (T6SS) components, including *vgrG14*, *PAAR2*, *PAAR*-*like* domain DUF4150 (PF13665), *tle3, pldA*, and *hcp2* homologues (File S1), were also present in the accessory genome. This range of T6SS components in the accessory genome warranted further investigation and suggests that the diversity of these components may provide *P. aeruginosa* with an advantage in the CF environment.

### Prevalence of characterised T6SS components varies within clinical *Pseudomonas aeruginosa* isolates

**Figure 3:**
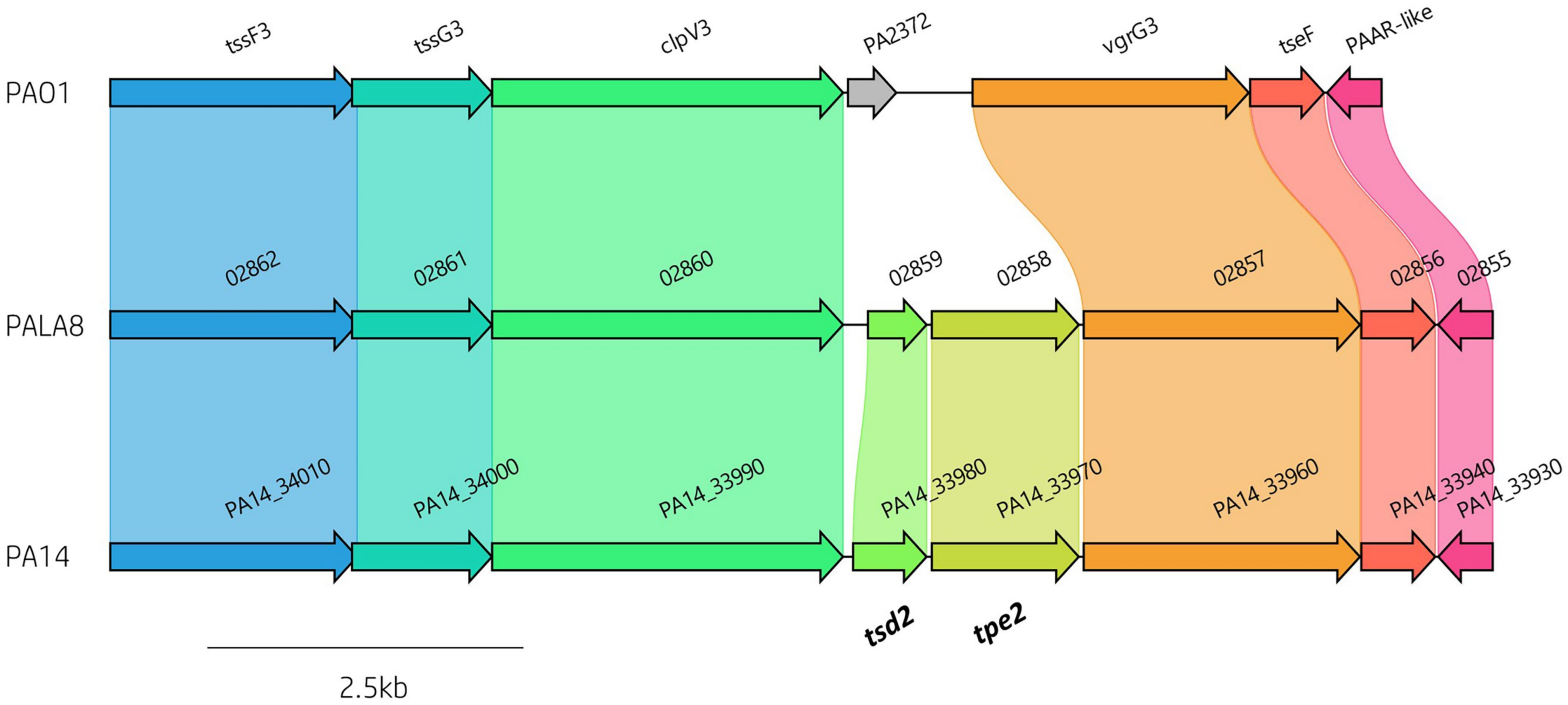
Comparative visualisation of the H3-T6SS cluster in PAO1, PA14 and PALA8 showing genomic differences in putative effector gene complement. The H3-T6SS cluster in PA14 and PALA8 contains *tsd2* that encodes a predicted cytidine deaminase effector and *tpe2* that encodes a predicted type VI peptidase effector.

To define the prevalence of 127 identified *P. aeruginosa* T6SS genes (Supplementary Table S2) amongst the 52 clinical *P. aeruginosa* isolates a BLAST screening method was used, confirming the effector encoding genes through visualisation in Artemis and ACT (Carver et al., 2005, 2012). All isolates were confirmed to possess the three central T6SS clusters (H1-, H2-, and H3-T6SS). Further, our analysis showed that 88 T6SS genes were highly conserved in all strains, of which 82 were seen in the pangenome calculated “core” genome. However, we also observed numerous intraspecific genomic differences at T6SS component loci, including within the main clusters (e.g., H3-T6SS; Figure 3; Supplementary File S2; Table S5). The number of *vgrG* genes within the clinical isolates ranged from eight to 13, whilst the number of *hcp* genes ranged from four to six. Isolate PALA8 was predicted to possess the highest abundance of both *vgrG*s and *hcp*s, encoding 13 and six, respectively (Table 1). Interestingly, two of the 13 *vgrG*s encoded by PALA8 appear to be exact copies of *vgrG3* and are downstream of the H3-T6SS cluster, inferring a genetic event that has trebled the cluster *tssG3* to *PAAR-like* (*PA2375*) and several neighbours, with a truncated version of *tssF3* in both the duplications. Manual checking of the long read assembly supports a genuine triplication of the region encoding *vgrG3* in the genome of PALA8, potentially providing insight into the creation of orphan *vgrG* islands in *P. aeruginosa*.

**Table 1:** Abundance of *vgrG* and *hcp* genes within the 52 *P. aeruginosa* genomes under study.

Isolate ID	Total No. of v*grGs*	Total No. of *hcps*	*vgrG14* island present	*vgrG7* island present
PALA26	11	6	Yes	
PALA27	9	5		
PALA28	9	5		
PALA29	9	5		
PALA30	11	6	Yes	
PALA32	9	5		
PALA33	8	4		
PALA34	9	5		
PALA35	12	6	Yes	Yes
PALA36	12	6	Yes	Yes
PALA37	10	5	Yes	Yes
PALA38	10	6	Yes	
PALA8	13	6	Yes	
PALA39	9	5		
PALA40	9	5		
PALA10	9	5		
PALA24	10	6		
PALA42	9	5		
PALA23	8	4		
PALA25	9	5		
PALA43	9	5		
PALA44	12	6	Yes	Yes
PALA9	9	5		
PALA45	9	5		
PALA22	9	5		
PALA20	11	6	Yes	
PALA12	9	5		
PALA6	9	5		
PALA7	9	5		
PALA47	9	5		
PALA48	9	5		
PALA16	9	5		
PALA15	9	5		
PALA4	11	6	Yes	
PALA18	9	5		
PALA50	9	5		
PALA3	9	5		
PALA1	10	4		Yes
PALA13	8	4		
PALA17	9	5		
PALA11	9	5		
PALA5	9	5		
PALA51	9	5		
PALA52	9	5		
PALA21	8	4		
PALA53	9	5		
PALA54	10	6	Yes	
PALA55	9	5		
PALA14	9	5		
PALA56	9	5		
PALA19	9	5		
PALA2	9	5		

**Figure 4:**
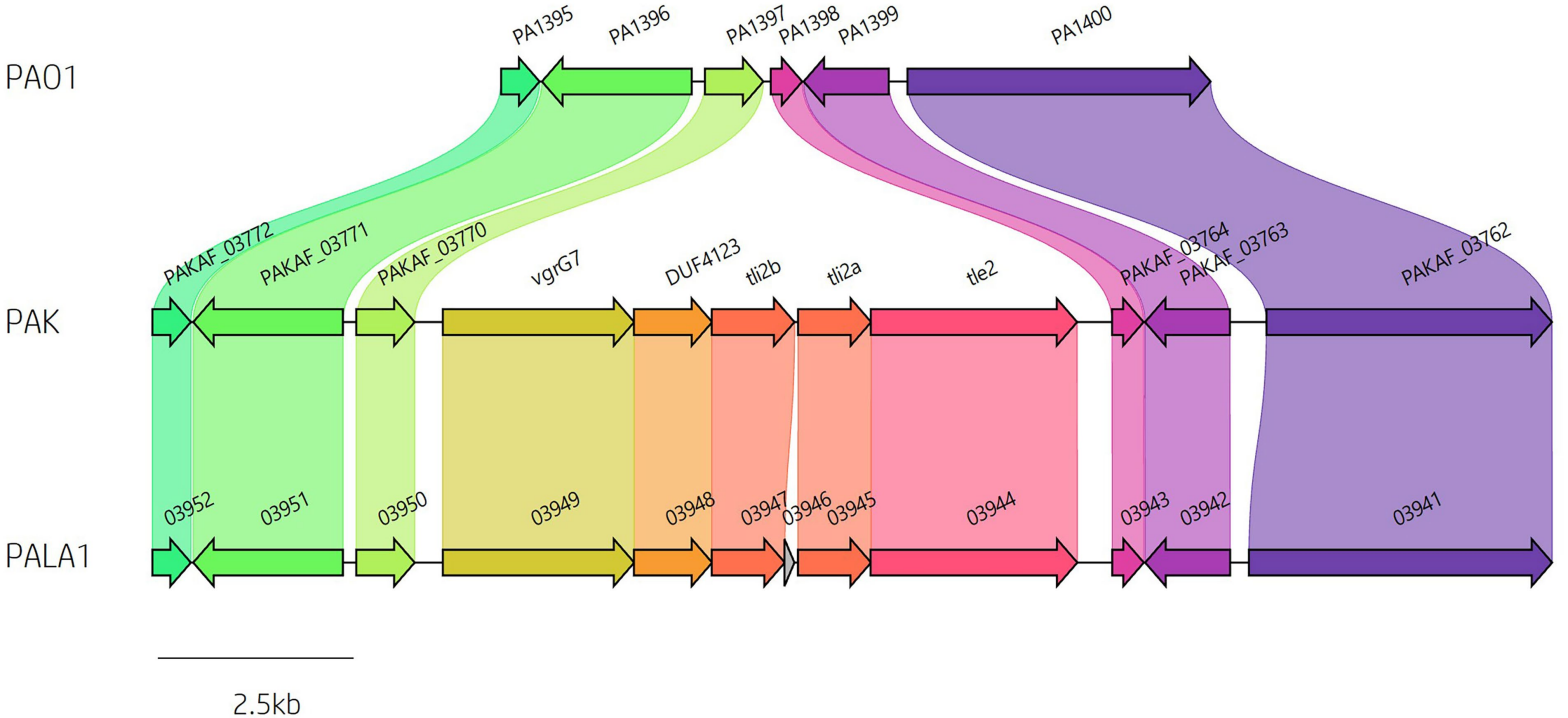
Identification of *vgrG7* and the *vgrG7* island. Genome sequence alignment between PAO1, PAK and clinical isolate PALA1 identifies the *vgrG7* island that contains *tle2*/*tli2* which encodes a putative lipase effector and immunity pair.

**Figure 5:**
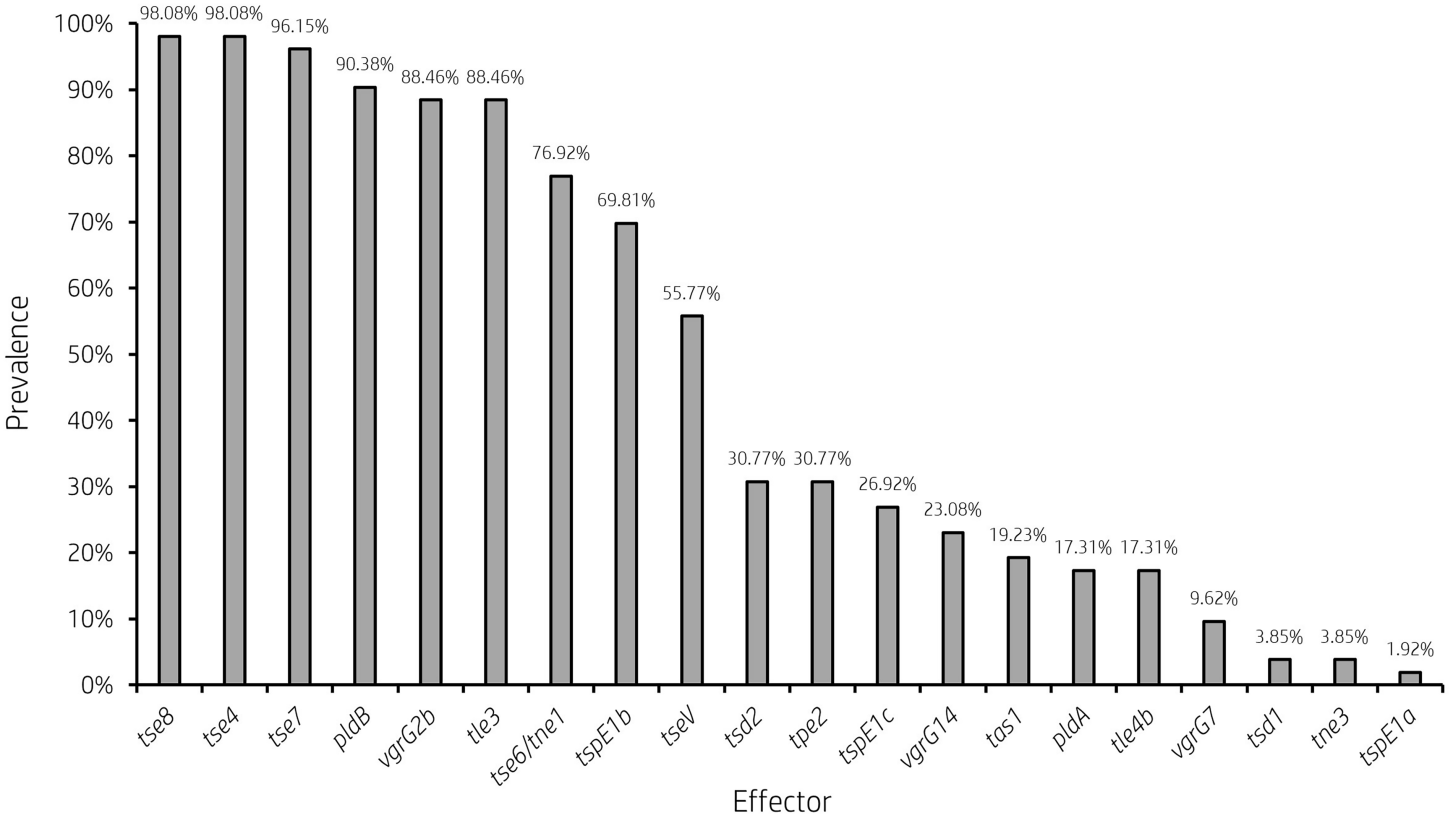
Prevalence of accessory T6SS effector encoding genes varies across clinical *P. aeruginosa* isolates. Bar graph showing the prevalence of select characterised and putative T6SS effector genes within 52 clinical *P. aeruginosa* isolates under study.

Analysis of *vgrG* and *PAAR* islands revealed 11 isolates out of the 52 investigated (21.15%) were predicted to possess the *vgrG14* island seen in PA14 (but not in PAK or PAO1) which is encoded divergently to the H2-T6SS cluster and harbours effector *rhsP2* (Hachani et al., 2014; Supplementary Figure S4), whilst five (9.62%) encoded the *vgrG7* island noted in *P. aeruginosa* strain PAK (discussed below; Figure 4; Table 1). Furthermore, two isolates (PALA36 and PALA44) were observed to have undergone a duplication event of the *PAAR2* island, integrating the *PAAR2* gene and cognate effector and immunity into the region of genome plasticity 28, previously designated by Mathee et al., which is adjacent to a tRNA^Pro^ encoding gene (Mathee et al., 2008; Klockgether et al., 2011). Notably, multiple isolates also lacked several previously identified T6SS gene islands, in addition to T6SS effectors distributed throughout the genomes. In total, six isolates (11.54%) were missing the *vgrG2b* island which encodes *hcpC*, *tle3*, *tli3* and *tla3*; 23 isolates (44.23%) lacked the *PAAR2* island which contains *tseV* and *tsiV*; and 43 isolates (82.69%) were missing the *vgrG4b* island which contains *pldA/tle5a* and *tli5a* (Figure 5; Supplementary Tables S5, S6). Several isolates were also missing other characterised T6SS effector genes (e.g., *pldB* and *tas1*; Figure 5; Supplementary Tables S5, S6).

It is clear from this data that interpatient diversity of T6SS components encoded within *P. aeruginosa* isolates is high with the presence of effectors varying between patients (Supplementary Tables S5, S6). Clinical isolates observed to lack T6SS effectors suggest the likelihood of isolates never having gained the effector encoding genes or genetic recombination resulting in the replacement of genes at effector loci with either scenario potentially possible. Further, clinical isolates that harbour unique subsets of effectors could provide advantages during competition and enable strains to distinguish between “self” and “non-self.” Indeed, it has been proposed that the acquisition of additional antibacterial toxin and immunity pairs may facilitate a bacterial ‘arms race’ between strains. This could provide a particular benefit in the context of a CF lung, with isolates able to differentiate kin from competitors and kill microorganisms that prevent colonisation or compete for resources.

### Comparative genomics reveals genetic diversity at several T6SS loci driven by mobile genetic elements

**Figure 6:**
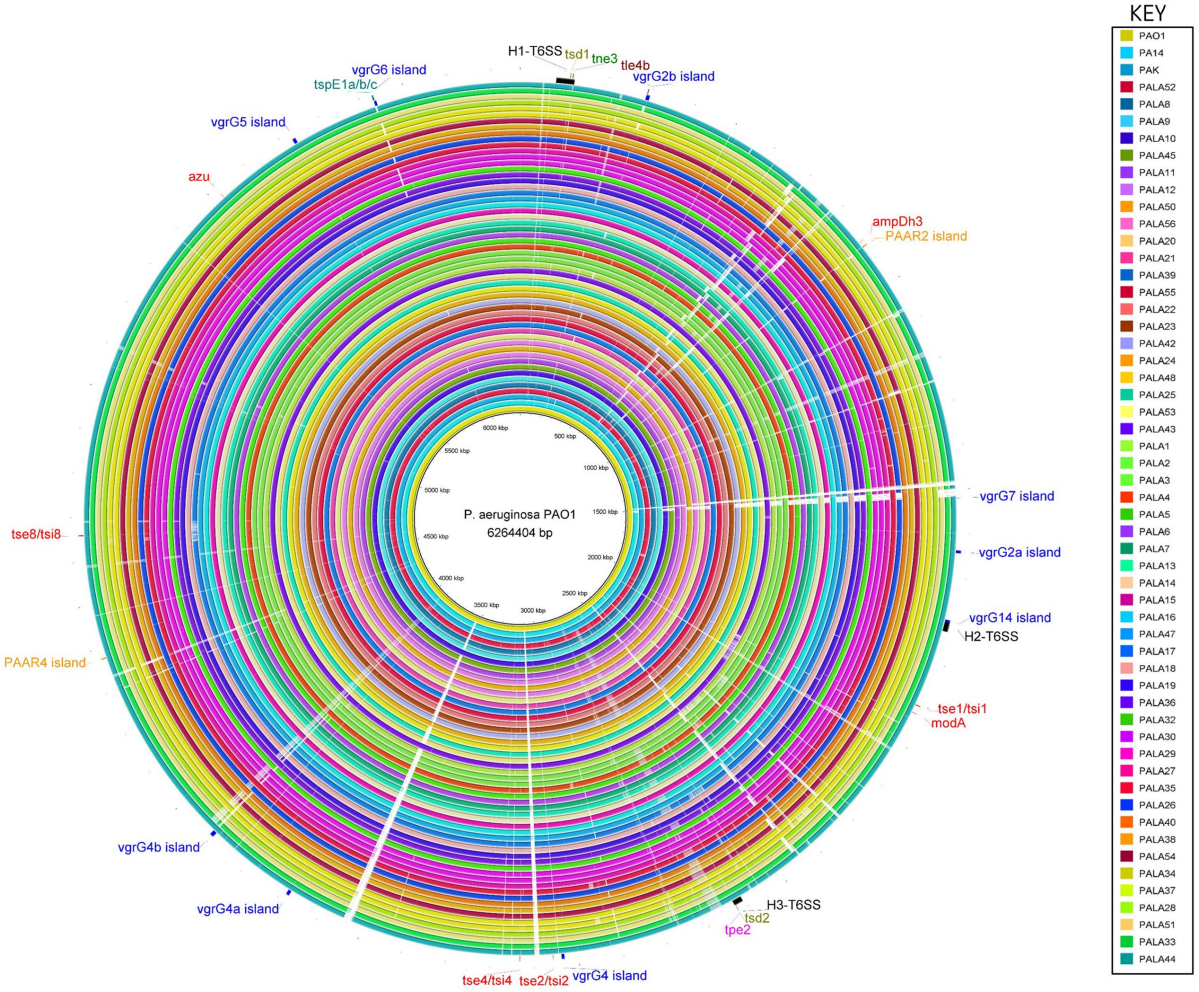
Comparative analysis of PAO1 to 52 clinical isolates under study and reference strains PA14 and PAK using the BLAST Ring Image Generator (BRIG) shows conservation of the genomes, and location, distribution and variation of T6SS genes. The innermost ring represents the genomic sequence of PAO1, followed by PA14 and then PAK. The remaining concentric rings represent the isolate sequences coloured according to the key. The intensity of the coloured ring signifies the genetic similarity of the reference sequence (PAO1) to the compared isolate (e.g., gaps = <50% similarity at the specific genomic loci). T6SS clusters, islands and effectors are labelled at the respective genomic loci in the reference strain PAO1. Central T6SS gene clusters are coloured black, *vgrG* islands are blue, *PAAR* islands are orange, and orphan effectors are red. The positions of the genes encoding the eight putative T6SS effectors described in this study are colour coded: putative cytidine deaminases (*tsd1*/*tsd2*) are olive, putative NADase (*tne3*) are green, putative lipases (*tle4b*) are maroon, putative metallopeptidase (*tpe2*) are pink, and putative pyocins are teal (*tspE1a*/*b*/*c*). The figure was produced using BRIG (Alikhan et al., 2011).

A comparative genomic approach against the best annotated reference strain PAO1 was then used to investigate strain-specific variation in genes encoding T6SS components. Multiple regions that possessed genetic differences (e.g., insertions, deletions) at T6SS loci spread throughout the genome were frequently identified in the clinical isolate genomes (Figure 6). Interestingly, our pangenome analysis showed the clinical *P. aeruginosa* accessory genome is enriched for HGT and MGE genes and are likely driving the differences observed.

To investigate this, we first focused our attention on the T6SS clusters. We observed that PALA9 possesses an insertion sequence (IS) element within the H2-T6SS cluster between the genes *orfX* and *fha2*, harbouring an IS1182 family ISPmo1 transposase (Supplementary Figure S5A). This has likely resulted in a truncated version of the *orfX* (*PA1664*) homolog (Supplementary Figure S5B), of which the implication is difficult to predict since no definitive function has been assigned to the gene. An additional example is present in isolate PALA22 which possesses a putative prophage (~39.6 kbp) integrated into the H2-T6SS cluster after the gene *tssA2,* an essential component of the H2-T6SS (Allsopp et al., 2017). Such insertion events could likely disrupt expression and potentially switch off the H2-T6SS or activate neighbouring gene expression.

**Figure 7:**
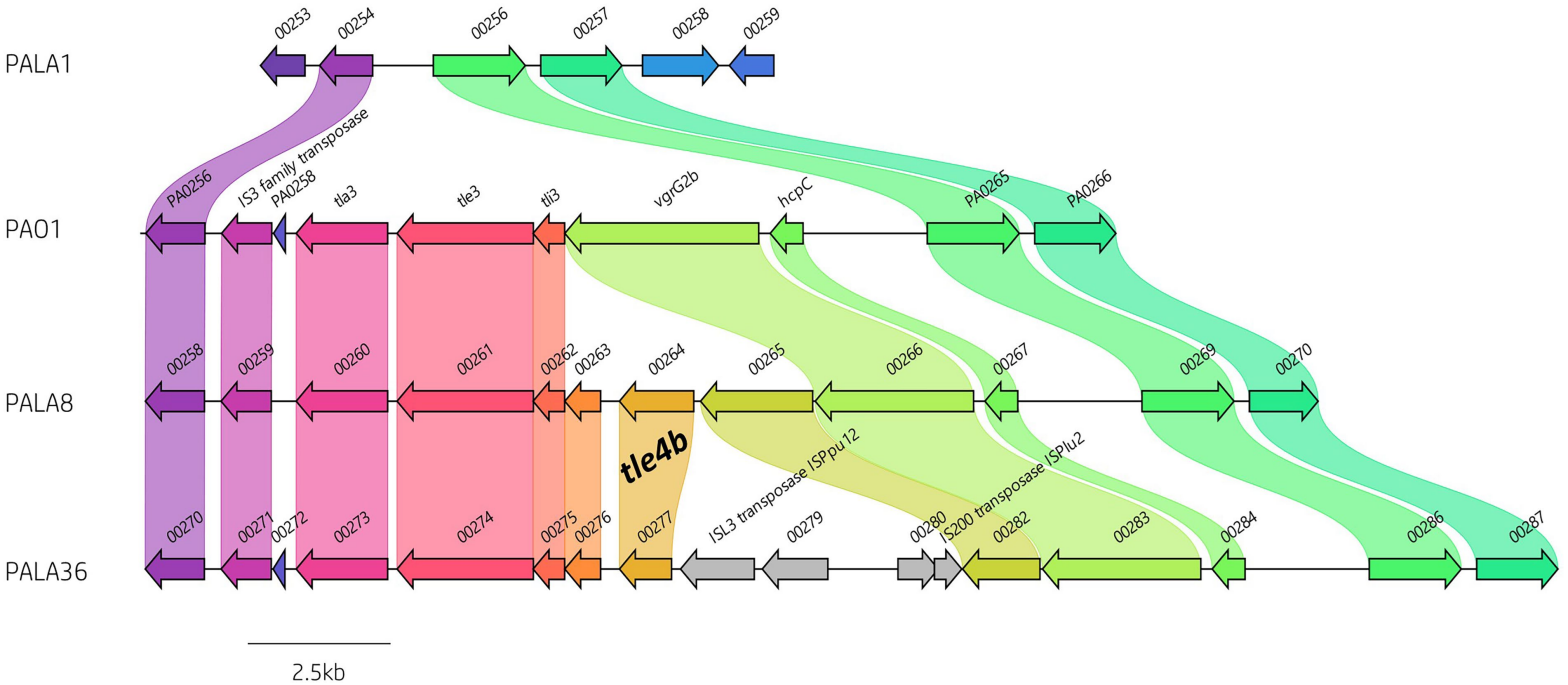
Comparative visualisation of the *vgrG2b* island highlighting the variable genetic architecture of this region in the genomes of different strains. PAO1 is the representative island example. Isolate PALA1 lacks the *vgrG2b* island. Isolate PALA8 possesses an additional gene encoding the putative T6SS effector *tle4b* (PALA8_00264) within the *vgrG2b* island. Isolate PALA36 is an example of the insertion of transposons into the island. Genes coloured the same with connected bands are homologs. Genes found in only one genome are in grey arrow. Arrowheads represent gene direction for transcription. Figure generated using Clinker (Gilchrist and Chooi, 2021).

Next, we investigated orphan *vgrG* islands for the impact of such genetic elements. The *vgrG2b* island provides a clear example of genetic variation caused by HGT/MGE. PAO1 harbours the classic *vgrG2b* island with a downstream gene encoding homology to an IS3 family transposase. In contrast, isolates cladding with PA14 and separately to PAO1 in the phylogenetic tree (Figure 2) contained a variety of differences. For instance, isolate PALA1 lacks the *vgrG2b* island, and isolate PALA8 possesses two additional genes, one of which encodes a new putative T6SS effector *tle4b* (PALA8_00264) that we identified within the *vgrG2b* island (discussed below). Two isolates (PALA36 and PALA44) were also observed to possess a transposon harbouring IS200 and ISL3 transposases, as well as a putative ArsP permease (PF03773, E-value 1.21e-45; PALA36_00279) that likely inactivate the *tle4b* genes in these strains (Figure 7).

Similarly, evidence of MGEs was observed for an orphan *PAAR* island, *PAAR2*. PAO1 encodes the best-annotated version of this island (Supplementary Figure S6; Wang et al., 2021); however, our analysis showed it was absent from 23 clinical isolates (representative example present in PALA11), either unintegrated or displaced by IS110 and/or IS3 transposases, MGEs, integrases, and/or HTH XRE-like genes (Supplementary Figure S6). These data suggest this locus is a target for insertion or recombination events leading to the loss of the virulence island. Additionally, we only observed one isolate (PALA21) that retained the *PAAR2* island whilst also possessing MGEs. PALA21’s *PAAR2* island contains two IS4 family ISPa45 transposases on either side of the operon and a downstream MGE. In addition to adding or disrupting operons, transposon and IS element insertion can result in upregulation of neighbouring gene transcription (Schnetz and Rak, 1992; Podglajen et al., 1994; Peschel et al., 1999).

These examples demonstrate that MGEs are active in proximity to T6SS genes which could result in gain, loss, or altered regulation of neighbouring genes. If these changes were advantageous in a particular niche and selected for, these could correspond to pathoadaptive mutations within *P. aeruginosa* (Winstanley et al., 2016).

**Figure 8:**
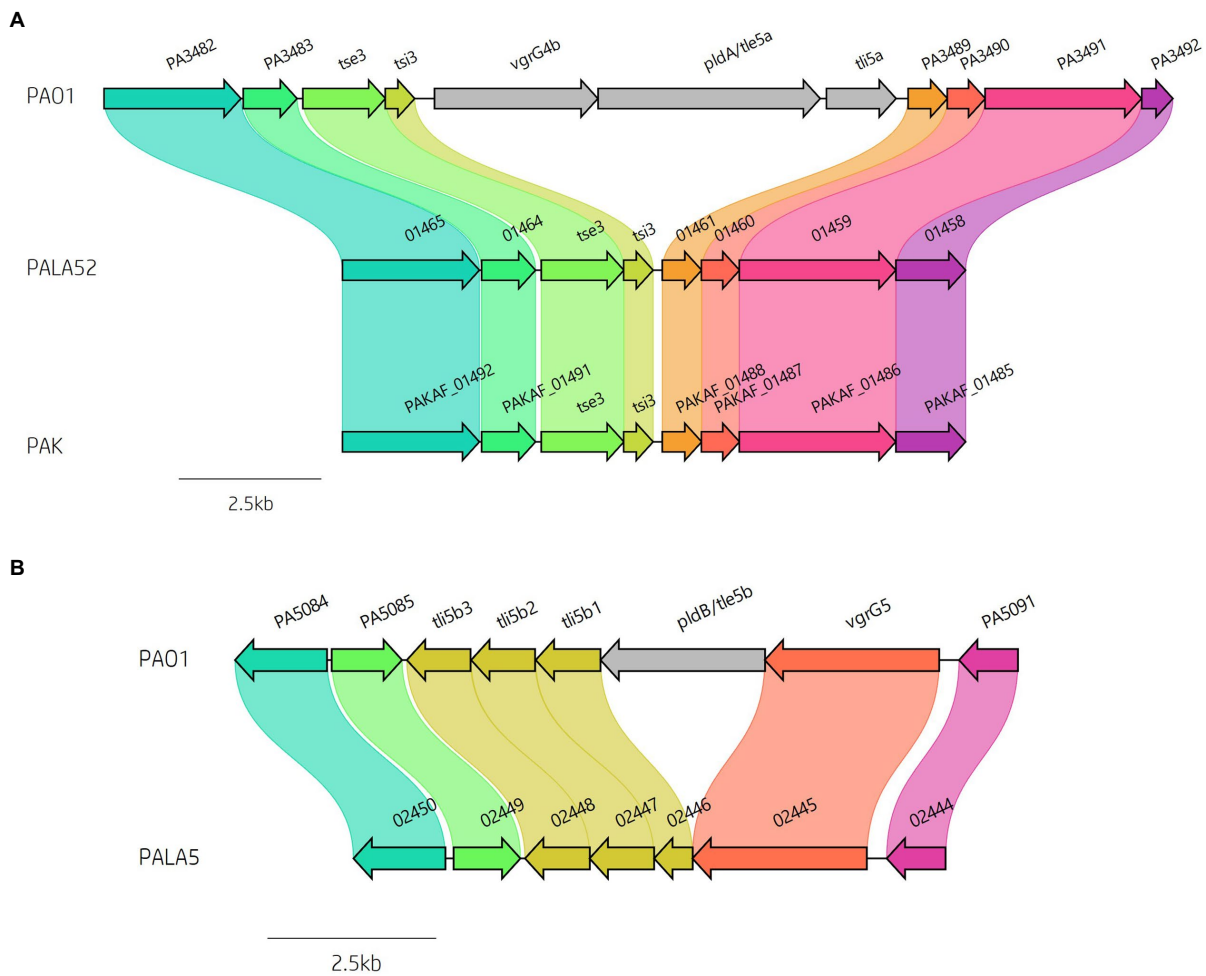
Comparative visualisation showing variation in the presence of the **(A)** *vgrG4b* and **(B)** *vgrG5* islands encoded within the genomes of clinical isolates compared to reference *P. aeruginosa* strains PAO1 and PAK. Isolate PALA52 and PAK are used as representatives for the absence of the *vgrG4b* island. Isolate PALA5 shows deletion of the region encoding the phospholipase effector *pldB/tle5b*.

We then focused our attention on the impact of these insertions on the effector gene complement of our strains of interest. As previously mentioned, multiple isolates in this study lacked several *vgrG* and *PAAR* islands, in addition to their cognate effectors that are part of *P. aeruginosa*’s arsenal of accessory genes. Discrepancies in effector presence are common amongst T6SS-harbouring bacteria (Unterweger et al., 2014; Vazquez-Lopez and Navarro-Garcia, 2020; Robinson et al., 2021), with strain variation as a consequence of random chance, diversification, independent survival requirements, and evolutionary selection. Previous studies have shown the *vgrG4b* island encoding *vgrG4b* and cognate EI pair *pldA/tle5a*-*tli5a* is absent from many strains including PAK, PA7, and several CF isolates (Jiang et al., 2014; Boulant et al., 2018; Subedi et al., 2018; Luo et al., 2019). In our isolates, 43 out of 52 (82.69%) lacked the *vgrG4b* island seen in PAO1 (Figure 8A; Supplementary Table S5), whilst retaining the upstream encoded EI pair *tse3*-*tsi3*, consistent with the prevalence amongst chronic CF isolates noted by Boulant et al. (Boulant et al., 2018). Furthermore, the second phospholipase effector characterised in *P. aeruginosa*, *pldB/tle5b*, located within the *vgrG5* island (Jiang et al., 2014; Wettstadt et al., 2019), was also missing in 5 isolates (PALA5, PALA7, PALA15, PALA32 and PALA47) leading to the predicted expression of a truncated *tli5b_1_* (PALA5_02446) without altering *tli5b_2_* or *tli5b_3_* (Figure 8B). The PA14-associated *vgrG14* island was observed in 11 (21.15%) isolates (Supplementary Figure S4; Table 1) and the EI pair *tse4*-*tsi4* was missing in only one isolate (PALA37; Supplementary Figure S7), as was the EI pair *tse8*-*tsi8* (PALA17); however, in PALA17, a different putative genomic island (~42 kb) was present at the same genomic location.

### The putative Tle2 lipase effector is encoded within the *vgrG7* island

**Figure 9:**
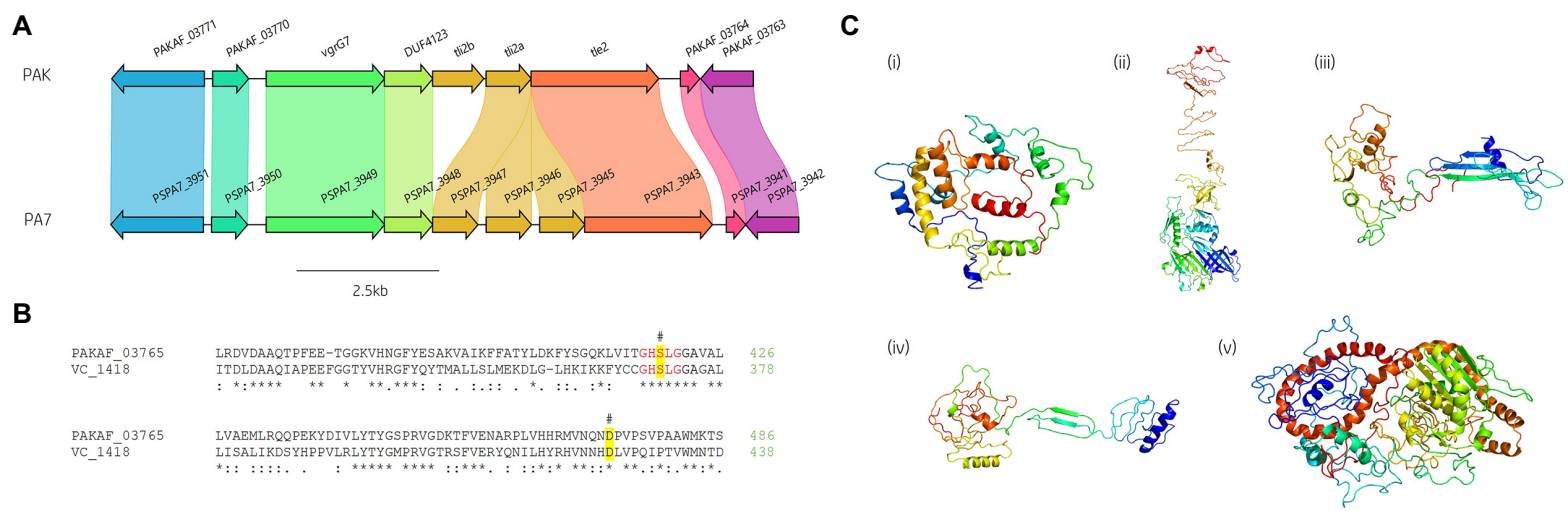
*In silico* analysis of the *vgrG7* island shows that it contains a putative lipase effector. **(A)** Comparative visualisation of the *vgrG7* island in reference *P. aeruginosa* strains PAK and PA7 (CP000744.1). **(B)** Partial amino acid sequence alignment of PAKAF_03765 (Tle2^PAK^) from PAK to Tle2^VC^ (TseL/VC_1418, AAF94575.1) from *Vibrio cholerae* O1 biovar El Tor str. N16961. The catalytic residues are indicated by a # and highlighted yellow. The GxSxG motif is highlighted in red. An asterisk (*) indicates fully conserved residues, a colon (:) indicates a strongly similar property at the residue, and a period (.) indicates a weakly similar property at the residue. The alignment was created using ClustalOmega (EMBL-EBI; Madeira et al., 2022). **(C)** Phyre2 predicted models of (i) DUF4123-containing protein (PAKAF_03768), (ii) VgrG7 (PAKAF_03769), (iii) Tli2_b_ (PAKAF_03767), (iv) Tli2_a_ (PAKAF_03766), and (v) Tle2 (PAKAF_03765).

A predicted uncharacterised *vgrG* gene and several genes comprising a new *vgrG* island were present in the accessory genome and found within the genomes of PAK and five of the clinical isolates in this study (Figure 4; Table 1). Following the *P. aeruginosa vgrG* nomenclature used in Hachani et al., we use the next available number denoting this gene as *vgrG7* (this cluster is also found in PA7, PSPA7_3949 [RefSeq: WP_012076464.1, Genbank: ABR82887.1]; Figure 9A). In all cases, the island is inserted between homologs of genes *PA1397* and *PA1398* from strain PAO1 (Figure 4). In PAK, VgrG7 (encoded by *PAKAF_03769*) possesses no identifiable C-terminal (CT) domains and aligns best to VgrG4a from PA14 (49% amino acid (aa) sequence identity) and groups with H2-T6SS secreted *vgrGs* (Supplementary Figure S8), suggesting that it would be coupled to the H2-T6SS for secretion. Downstream of the *vgrG7* gene is *PAKAF_03765,* encoding a protein that is predicted to possess a lipase (class 3) domain (cd00519, aa 288-494) and fits with the previously identified Tle2 family (Russell et al., 2013). Structural homology searching using Phyre2 returned multiple hits, with the top 5 being fungal lipases (6QPP_A, 3TGL_A, 3UUE_A, 1TIB_A, and 1DUWC_A - all with 100% confidence). All lipases identified with the Phyre2 search belong to triglyceride lipases (EC 3.1.1.3) designated class 3 lipases (PF01764) and are members of the α/β-hydrolase superfamily with catalytic residues Ser-Asp-His (Moroz et al., 2019). To determine the sequence similarity of Tle2^PAK^ to a characterised Tle2 lipase, Tle2^VC^ (TseL/VC_1418) from *Vibrio cholerae* O1 biovar El Tor str. N16961, an amino acid alignment was conducted confirming the catalytic motif GxSxG and aspartate-425 (D, #) is conserved in both proteins (Figure 9B; Dong et al., 2013; Russell et al., 2013). A candidate residue for the Histidine catalytic residue could not be determined in Tle2^PAK^. Collectively, this data suggests that the protein encoded by *PAKAF_03765* belongs to T6SS lipase family Tle2 and possesses part of the Ser-Asp-His catalytic triad common for lipases.

Protein function prediction of the neighbouring genes to *tle2*^PAK^ revealed two putative lipase immunity proteins in PAK (PAKAF_03767 [aa 298] and _03766 [aa 263]) which share 75% aa identity with each other (Figure 4). However, the *vgrG7* island in PA7 appears to harbour an additional *tli2* immunity gene alongside with *tli2_a_* and *tli2_b_* (Figure 9A). Prediction of signal peptides by SignalP 5.0 for both 03767 (Tli2_b_^PAK^) and 03766 (Tli2_a_^PAK^) identified an N-terminal lipoprotein signal peptide (Sec/SPII, Likelihood: 0.9169 and 0.8955, respectively) with cleavage sites predicted between residues 19 and 20 (Probability: 0.9167 and 0.8967, respectively) in both proteins. Further, both proteins were predicted to possess the lipobox motif ‘AQGC (+1)’, plus an L residue at position (-4) which may play a function in the motif. The ‘+2 rule’ suggests both proteins are translocated to the outer membrane (Zückert, 2014). In addition to the lipase effector and immunity genes, a putative DUF4123-encoding gene was also observed between the genes *vgrG7* and *tli2_b_* in PAK (Figure 4 and Figure 9Ci). DUF4123 is a superfamily domain associated with T6SS chaperone/adaptor proteins, directly interacting with their cognate effectors to transport them to the VgrG protein complex before secretion (Liang et al., 2015; Unterweger et al., 2015). It is therefore likely that this DUF4123 protein mediates the passage or coupling of the putative effector Tle2^PAK^ to its cognate *vgrG,* VgrG7. Phyre2 structure models of the predicted proteins are shown in Figure 9C, where the model of the VgrG7 monomer shows a densely folded N-terminus possessing a protruding polypeptide extension rotating towards the C-terminus (Figure 9Cii) and the putative puncturing region which is fully formed as a trimer *in situ*. Both putative immunities Tli2_a_ and Tli2_b_ (Figure 9Ciii and iv) share structural homology, possessing distinct N-and C-terminal regions linked by a central peptide chain which forms at least two β strands. The putative effector Tle2^PAK^ appears to possess a long α helical chain (Figure 9Cv), however, no discernible features from the model could further elucidate the mechanism by which the effector exhibits its putative lipase function. Collectively, our analysis identifies a new *vgrG7* island from PAK, PA7, and five clinical isolates that encodes a putative antibacterial Tle2 family lipase which may be used during competition to colonise the CF lung. These data further highlight that additional T6SS components and effectors remain uncharacterised from common *P. aeruginosa* strains.

### *In silico* analysis reveals eight putative T6SS effectors within clinical *Pseudomonas aeruginosa* isolates

Building on our analysis above where we first collated all know *P. aeruginosa* T6SS protein sequences (Supplementary Table S2) and used these as inputs for a tBLASTn approach, comparative genome visualisation using BRIG, Artemis and Clinker lead to the identification of eight regions encoding putative uncharacterised T6SS effectors with clear genetic diversity to reference strain PAO1. These eight effectors were encoded in proximity to know T6SS components and are genetically associated with all three T6SS systems (H1-, H2-, H3-T6SS) in our reference and clinical strains. Subsequent analysis was performed with a suite of protein function predictions tools (see Methods) to identify conserved domains, motifs and structural similarities to uncover the putative toxic activities of these effectors. Genes encoding for eight new putative effectors were identified within the clinical isolates (Figure 1), where two are shared with strain PA14 and one with strain PAO1. Their predicted functions are outlined below.

#### Identification of two Cytidine Deaminases putative effectors

**Figure 10:**
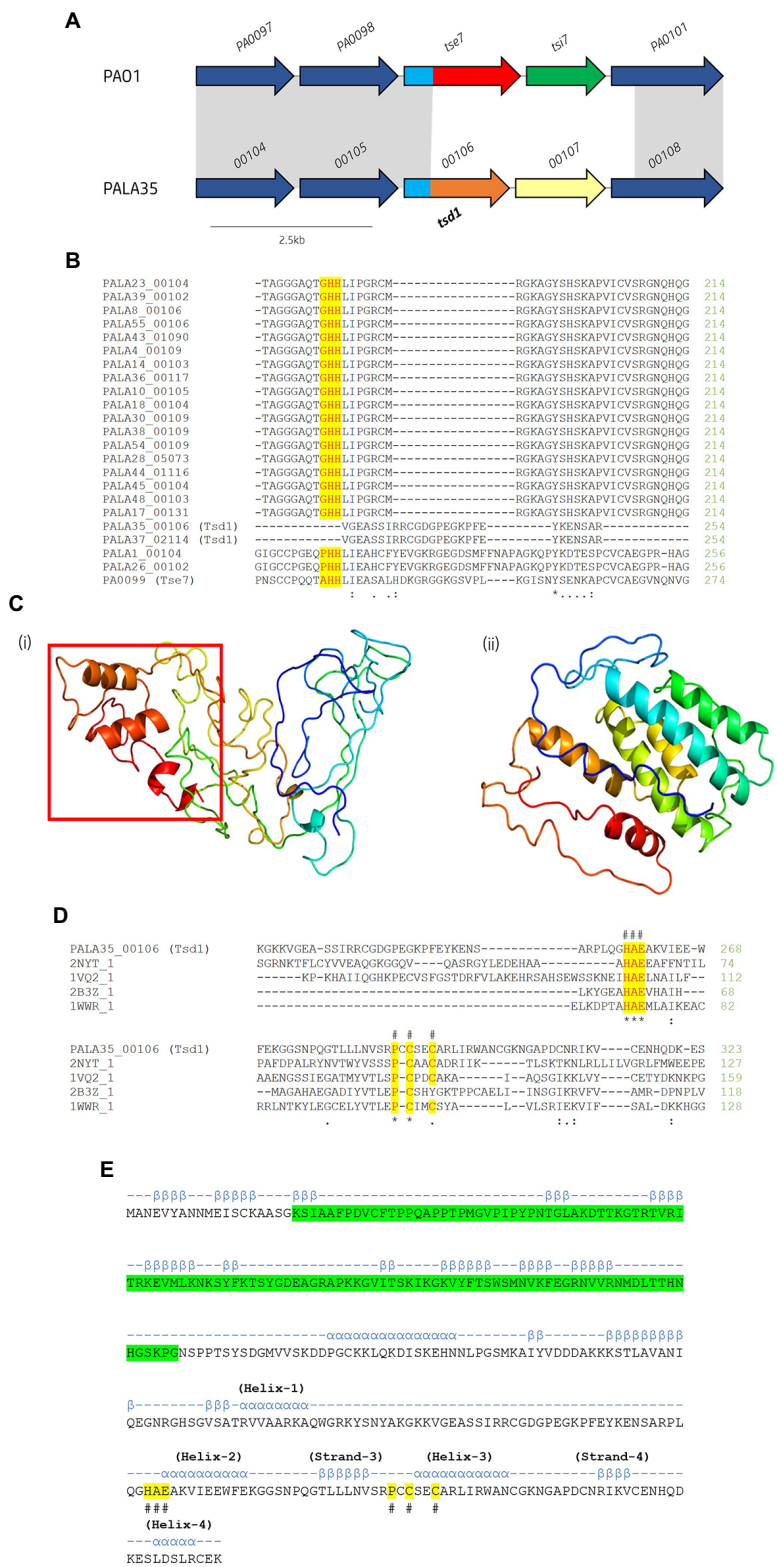
*In silico* analysis of putative cytidine deaminase Tsd1. **(A)** Schematic of the orphan effector-immunity island *tse7*-*tsi7* in reference *P. aeruginosa* and representative clinical isolate PALA35. Homologous genes are connected by grey bands. Proteins of unknown function are coloured dark blue. The N-terminal PAAR-like domain DUF4150 is light blue [aa 1-135]. The variable C-terminal is coloured red for the domain Tox-GHH2 in *PA0099/tse7* [aa 135-315] and light brown for the C-terminal cytidine deaminase domain in *PALA35_0106/tsd1* [aa 135-331]. Cognate immunities are coloured green for *PA0100/tsi7* and yellow for *PALA35_0107/tsdI1*. **(B)** Partial amino acid sequence alignment of Tse7 homologs from the clinical isolates to Tse7 (PA0099) from reference strain PAO1. Residues of the characteristic GHH motif of Tox-GHH2 are highlighted yellow. Note the absence of the GHH motif from PALA35/36 Tsd1 suggesting Tsd1 has a different mechanism of action **(C)** Phyre2 predicted models of (i) Tsd1 and (Continued)FIGURE 10 (Continued)(ii) TsdI1. The red box highlights the location of the four α helical toxic activity-containing C-terminus. **(D)** Tsd1 is a putative deaminase as shown by amino acid alignment of Tsd1 from isolate PALA35 (00106) to deaminase APOBEC-2 from *Homo sapiens* (PDB: 2NYT_1), T4-bacteriophage deoxycytidylate deaminase from *Escherichia* virus T4 (PDB: 1VQ2_1), bifunctional deaminase Riboflavin biosynthesis protein RibD from *Bacillus subtilis* (PDB: 2B3Z_1), and tRNA adenosine deaminase TadA from *Aquifex aeolicus* (PDB: 1WWR_1). Residues of the HAE and PCxxC deaminase motifs are highlighted yellow and indicated by an #. Additional symbols are the same as in Figure 8. **(E)** Tsd1 encodes a putative PAAR-like DUF4150 domain in addition to the deaminase motifs. PSIPred secondary structure prediction of Tsd1 with the PAAR-like domain DUF4150 (PF13665) highlighted in green, alpha-helices are annotated by α and Beta-strands by β. The location of important α and β structure of the cytidine deaminases superfamily as described by Iyer et al. (Iyer et al., 2011). Labelled HAE and PCxxC motifs are highlighted in yellow and denoted with #.

Cytidine deaminases (CDA) are hydrolytic enzymes responsible for the deamination of cytidine and 2′-deoxycytidine to uridine and 2′-deoxyuridine, respectively (Navaratnam and Sarwar, 2006). Bacterial deaminase toxins are predicted to mutate target nucleic acid leading to RNA editing, DNA hypermutations, altered gene expression, or providing an anti-viral defence (Iyer et al., 2011). To date, only one T6SS effector possessing deaminase toxicity has been characterised (Mok et al., 2020), although several have been predicted including XOO_2897 from *Xanthomonas oryzae pv. oryzae* MAFF311018 (Iyer et al., 2011). Within the isolates under study, we identified two putative T6SS effectors possessing CDA functions which we will refer to as Type six cytidine deaminase effectors 1 (Tsd1) and 2 (Tsd2; Figures 3, 10; Supplementary Figure S9).

Within the isolates under study, we observed 22 (42.31%) isolates possessing significant genetic diversity at the loci of EI pair *tse7*-*tsi7* (Figure 10A). Multiple sequence alignment of the isolates’ Tse7^PAO1^ (PA0099) homologs revealed lengths ranging from 315 to 386 aa (Figure 10B). All 22 homologs were predicted to possess the PAAR-like domain DUF4150 (PF13665) in their N-terminus, classifying them as PAAR subtype E2 (Zhang et al., 2021). Alignment of the 22 homologous CTs confirmed 20 possessed the Tox-GHH2 domain (PF15635) in their CT (Pissaridou et al., 2018), however, two *tse7* homologs, *PALA35_00106* and *PALA37_02114*, did not (Figure 10B). Proteins PALA35_00106 and PALA37_02114 possessed 100% aa sequence identity to each other. A JackHMMER search against the UniProtKB database for three iterations returned only four protein sequences possessing homology to the screened protein PALA35_00106-CT [aa 135-331]: Q058_04662 (Evalue: 2.4e-117) from *P. aeruginosa* BL04, SAMN04244572_02496 (6.9e-47) from *Azotobacter beijerinckii* DSM 373, AWB83_01173 (4.4e-42) from *Caballeronia ptereochthonis*, and ENH01_03070 (6.2e-32) from *Nitrospirae bacterium* HyVt-268. All matches possessed identical domain architecture, harbouring the PAAR-like DUF4150 (PF13665) domain in their N-terminus and no identifiable domains in their CT. Q058_04662 possessed 100% aa identity to PALA35_00106 and is harboured at the same genomic loci. The three other proteins were also found to be encoded to neighbouring *vgrG* genes at homologous T6SS cluster loci. Structural homology of the PALA35_00106-CT [aa 135-331] using Phyre2 and DALI matched with 86.1 confidence to a Hydrolase/Deoxycytidylate (dCMP) deaminase (PDB: 4P9E_A. DALI Z-score: 4.5) from cyanophage S-TIM5, and 82.4 confidence to a CDA-like tRNA adenosine deaminase TadA (PDB: 1Z3A. DALI Z-score: 3.5) from *E. coli* (Figure 10Ci). Thus, we label PALA35_00106 Tsd1 as it is likely to encode a cytidine deaminase effector. In further support, a cytidine and deoxycytidylate deaminase zinc-binding region (PS00903) at aa 259-296 was also predicted using PROSITE and is similar to that seen in antibacterial T6SS toxin DddA from *Burkholderia cenocepacia*, a dsDNA CDA (Mok et al., 2020; de Moraes et al., 2021). Finally, a sequence alignment and secondary structure prediction of Tsd1 identified conserved secondary structure regions, including the HAE (helix-2) and PCxxC (helix-3 zinc ion binding) motifs from the ‘Helix-4’ division of the CDA superfamily categorised by Iyer et al. (Figures 10D,E; Iyer et al., 2011).

A putative cognate *tsd1* immunity gene was then identified in these two isolates (*PALA35_00107* and *PALA37_02113*) and were encoded at the same genomic loci as *tsi7* in PAO1, which we have denoted as Type six cytidine deaminase immunity family 1 (TsdI1). The gene *tsdI1* is predicted to encode a small protein (~24.1 kDa), sharing only 17% aa identity with Tsi7^PAO1^ and has no identifiable domains or transmembrane helices (Figure 10Cii). CELLO2GO (Yu et al., 2014) predicted TsdI1 to exhibit subcellular localisation at the cytoplasm (Score: 5.485), consistent with the putative target location of Tsd1.

A second putative T6SS CDA effector was predicted in 16 (30.77%) clinical isolates and reference strain PA14. This putative effector, which we refer to as Tsd2, is encoded within the H3-T6SS cluster between genes *clpV3* and *vgrG3* in PA14 (*PA14_33980*; Figure 3). Protein homology searches using HHpred matched Tsd2 to a CDA blasticidin S deaminase from *Aspergillus terreus* (Probability: 95.65, 2Z3G_C, E-value 0.18). Congruency was observed when aligning Tsd2 and CDA (Iyer et al., 2011) protein sequences (Supplementary Figure S9A), with conserved synapomorphies to the ‘C-terminal hairpin’ division, including a CxE signature (metal-chelating motif at the beginning of helix-2) and PCxxCR motif after predicting the secondary structure (Supplementary Figure S9B; Iyer et al., 2011). Unlike Tsd1, no cognate immunity could be predicted for Tsd2, suggesting putative eukaryote targets. RNAseq analysis additionally shows that *tsd2* (*PA14_33980*) was significantly upregulated along with other H3-T6SS components suggesting it is co-expressed with the H3-T6SS (Allsopp et al., 2022).

Phylogenetic analysis was then conducted to determine the relationship between the two putative CDA effectors, Tsd1 and Tsd2, from our clinical strains to other CDA toxins. Tsd1 and Tsd2 clade into two different CDA groups and fits with their categorisation into different CDA superfamilies. We observed that Tsd1 is most closely related to homologs identified using the JackHMMER search, and Tsd2 groups with predicted effector XOO_2987 from *Xanthomonas oryzae* (Supplementary Figure S10).

To conclude, we have observed the presence of two putative T6SS effectors that encode predicted CDA functions in several clinical *P. aeruginosa* genomes and reference strain PA14. The putative effector *tsd1* possesses a cognate immunity, *tsdI1*, suggesting it may be invloved in interbacterial competition, providing a level of strain-specificity useful to invading strains when trying to survive in environments such as the CF lung. The second putative effector, *tsd2*, does not encode any observable immunity protein and is therefore predicted to target eukaryotes. Based on the genetic proximity of the effector *tsd2* to the H3-T6SS and *vgrG* gene *vgrG3*, it is likely this effector and neighbouring gene, PA14_33970 (discussed below), are secreted by the H3-T6SS cluster.

#### Identification of one putative NADase effector

Recently, the genomic loci encoding T6SS effector *tse6* was noted to vary between *Pseudomonas aeruginosa* strains, with the PA14 homolog (Tas1^PA14^) displaying pyrophosphorylation activity of adenosine nucleotides to produce (p)ppApp (Ahmad et al., 2019), whilst the PAO1 homolog (Tse6^PAO1^/Tne1) exhibits NAD(P)^+^-glycohydrolase activity (Whitney et al., 2015). Both effectors contain a conserved PAAR_Rhs domain in their N-terminus, but distinct sequences in their CTs. Tse6^PAO1^ also possesses an mART fold observed in other mART-encoding toxins including diphtheria toxin and ExoA from *P. aeruginosa* (Simon et al., 2014). Tse6^PAO1^ was categorised as belonging to the Type VI NADase effector family 1 (Tne1) after the characterisation of Tne2 in *Pseudomonas protegens* Pf-5 (Tang et al., 2018).

**Figure 11:**
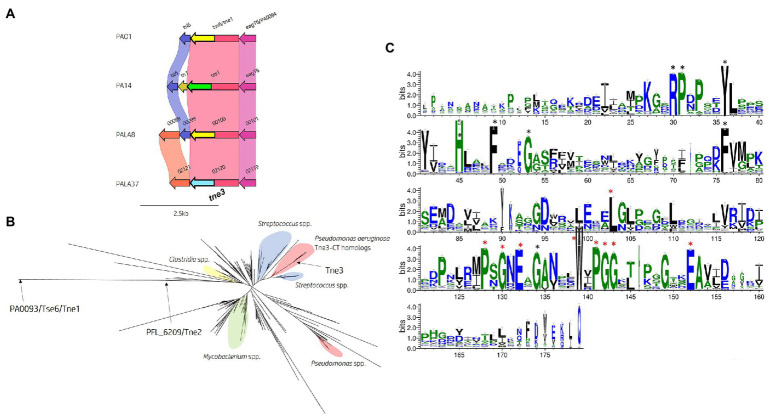
*In silico* analysis of the *tse6* genomic loci reveals diversity across multiple *P. aeruginosa* strains and identifies *tne3* as encoding a different effector class. **(A)** Comparative visualisation of the Tse6 effector loci in PAO1, PA14 and clinical isolates PALA8 and PALA37 shows variation in the immunity genes. Variation in the C-terminal region of Tse6, Tas1 PALA8_00100 and PALA37_02120 is indicated by the C-terminal coloured arrows but masked in the coloured connecting bands of the homology mapping due to the overall homology of the proteins. C-terminal toxic domains of *tse6/tne1* homologs are yellow, *tas1* homologs are green, and *tne3* homologs are blue. **(B)** Maximum likelihood phylogenetic tree derived from Tne3 homologous sequences identified from a JackHMMER search against Tne1 (PA0093/Tse6^PAO1^) from PAO1 and Tne2 (PFL_6209) from *P. protegens* Pf-5 using MEGAX. Select clades have been highlighted due to consensus clustering blue = *Streptococcus* spp., yellow = *Clostridium* spp., red = *Pseudomonas* spp., and green = *Mycobacterium* spp. Tne1, 2, and 3 are labelled with arrows. Phylogenetic analysis was conducted in MEGAX using the WAG model, G + I (n = 5), bootstrap n = 500, “partial deletion (95%),” and NNI ML heuristic model parameters. The tree was visualised using the iTOL (v6) webserver (Letunic and Bork, 2021). **(C)** WebLogo3 visualisation of the most frequent amino acids present in Tne3 homologs. Conserved residues are labelled with a black star and residues found in 90-100% of sequences aligned are labelled with a red star and are likely important for peptidase activity. Visualised using WebLogo3 (Crooks et al., 2004).

Within the isolates under study, 40 encoded homologs to Tse6^PAO1^/Tne1 and 10 possessed Tas1^PA14^ homologs. However, we uncovered two isolates (PALA35 and PALA37) harbouring a homolog of Tse6 encoding an N-terminal PAAR_Rhs domain (cd14742), classifying them all as PAAR subtype A2 (Zhang et al., 2021), but with a distinct CT (Figure 11A). Analysis of the protein PALA37_02120, which we refer to as our third new toxin designated as Type VI NADase effector family 3 (Tne3), revealed no identifiable domains and 61 and 62% aa identity to Tse6^PAO1^ and Tas1^PA14^, respectively. A BLASTp search of Tne3 in the *Pseudomonas* Genome Database identified 12 homologous hypothetical proteins, including PSPA7_0167 from *P. aeruginosa* strain PA7 (98.9% aa identity) and *Pseudomonas corrugata* RM1-1-4 protein AXG94_RS14835 that is encoded immediately downstream of a T6SS cluster homologous to the H1-T6SS. The putative function of the effector was revealed after structural homology searches using HHpred and Phyre2 matched the CT of Tne3 [aa 255-434] to a β-NAD^+^-glycohydrolase (4KT6_A, Probability 98.25%, E-value 2.7e-5) and NAD^+^-glycohydrolase SPN (3PNT, 91.1% Confidence, aa 256-382) from *Streptococcus pyogenes* (Supplementary Figure S11A). SPN is a well-characterised *S. pyogenes* virulence factor which contributes to pathogenesis (Ajdic et al., 2000; Ghosh et al., 2010). The toxin is injected into the host cell membrane before translocation to the cytosol where it hydrolyzes β-NAD^+^ to produce nicotinamide and adenosine diphosphoribose (Madden et al., 2001; Ghosh et al., 2010). *In vitro* and *in vivo* studies revealed that SPN is highly cytotoxic to yeast and epithelial cells, with only inactive mutants noncytotoxic in animal models (Madden et al., 2001; Bricker et al., 2005; Ghosh et al., 2010). Alignment of the amino acid sequence of Tne3 to SPN and Tse6^PAO1^ was attempted to identify the characteristic NAD^+^ binding pocket and distinctive residues noted in both proteins but was unsuccessful (Ghosh et al., 2010; Smith et al., 2011; Chandrasekaran et al., 2013; Whitney et al., 2015).

To identify any homologs of the Tne3-CT [aa 255-434], we performed a JackHMMER search against the UniProtKB database. After five iterations, this search matched 545 sequences belonging to multiple taxa including *Firmicutes* (*n* = 198) and *Proteobacteria* (n = 194), of which 86 belonged to *Pseudomonas* spp. and 34 to *Enterobacterales*. Exploring the domain architecture of sequences homologous to the CT of Tne3 revealed 364 sequences with no identifiable domains, 65 with PAAR domains in their N-terminus, 35 with LXG domains, 18 with Fil_Haem, TPS, PT-VENN, DUF637, and/or ESPR domains, and 47 with RHS_repeats. Collectively, this data shows that DNA encoding this CT exist in polymorphic toxins belonging to the T5bSS (Fil_Haem, TPS, PT-VENN, DUF637), T6SS (PAAR) and T7SS (LXG), and strongly supports evidence of a widespread toxin domain. Next, all search hits for Tne3 and amino acid sequence of Tne1 and Tne2 were aligned to construct a phylogenetic tree (Figure 11B). This tree illustrates Tne toxin diversity but shows the close phylogenetic clustering of Tne3 to SNP proteins from *Streptococcus* spp. than the Tne1 or Tne2 clades, confirming the predicted similarity identified by Phyre2. Using a PSSM-driven methodology of Tne3-CT [aa 255-434], we identified 18 conserved residues of which 9 were within a stretch of 25 amino acids covering a PxGxE and a WxPGG motif that were present between 90-100% of sequences aligned (Figure 11C), suggesting they may be important for effector activity.

Finally, we investigated the presence of a cognate immunity to *tne3*. Comparative genomic analysis of the Tse6 locus (Figure 11A) revealed an upstream gene (PALA37_02121) to *tne3* (PALA37_02120) which we denote as Type VI NADase immunity family 3 (Tni3). Structural homology searching using Phyre2 matched Tni3 to immunity factor IFS (spy0166) associated with the NAD^+^-glycohydrolase SPN toxin from *S. pyogenes* (3QB2_B, Probability 100%, E-value 1.7e-35; Supplementary Figure S11B). IFS has been shown to inhibit SPN activity (Meehl et al., 2005), therefore Tni3 is likely the putative immunity to Tne3. We also observed *tni3* (Figure 11A) present in another clinical isolate (PALA8_00097, 100% aa identity) that carried *tne1/tni1* (*tse6*^PAO1^-*tsi6*^PAO1^*)* seen in strain PAO1. Tsi6^PAO1^ and PALA8_00099 were predicted to share 98% aa identity and Tse6^PAO1^ and PALA8_00100 were predicted to share 99% aa identity. From this data, it appears PALA8 encodes immunity genes against both Tne1/Tse6^PAO1^ and the putative effector Tne3.

In conclusion, these data identify the genes encoding a new putative EI pair (Tne3-Tni3) that likely functions as a NADase effector that is encoded at the same genomic loci as *tne1/tse6*^PAO1^ and *tas1*^PA14^ and is present in select clinical strains (Figures 5, 11).

#### Identification of one putative lipase

We observed evidence of genetic recombination within the *vgrG2b* island that has resulted in the insertion of a putative lipase EI module (PALA8_00265/4) downstream of *vgrG2b* (Figure 7). This genetic event was noted in 9 isolates (17.31%) within this study and the putative lipase EI pair was conserved throughout. Previously, Wood et al. putatively classified this additional EI pair as homologous to neighbouring EI pair *tle3-tli3* (Wood et al., 2019a); however, our analysis predicts the inserted effector belongs to the lipase effector family 4 (Russell et al., 2013; Jiang et al., 2016), and in keeping with nomenclature we refer to the protein as Type VI lipase effector family 4b (Tle4b; Figure 7; Supplementary Figure S12).

Domain analysis using NCBI-CDD confirmed the protein encoded by *tle4b* putatively harbours an α/β-hydrolase (cl21494; E-value 2.59e-05) superfamily domain associated with lipases. Sequence alignment of Tle4b to Tle3^PAO1^ (PA0260) and Tle4a/TplE^PAO1^ (PA1510, Type VI lipase effector family 4a (Tle4a)) from strain PAO1 revealed 16 and 27% aa identity, respectively. Tle4a/TplE is a trans-kingdom effector that induces autophagy of eukaryotic cells through endoplasmic reticulum stress and also contributes to inter-bacterial killing (Jiang et al., 2016). Investigation into the alignment revealed a conserved region in both Tle4a and Tle4b containing the TxSxG motif (Supplementary Figure S12A) and overlap at the eukaryotic PGAP1-like domain (PF07819) seen in Tle4a/TplE^PAO1^ (Lu et al., 2014; Jiang et al., 2016), as well as the catalytic triad Ser-Asp-His (Russell et al., 2013). Congruently, when Tle4b was subject to a structural homology search using HHpred and Phyre2, aa 6-581 matched to Tle4 from *P. aeruginosa* strain PAO1 (4R1D_A, Probability 100%, E-value: 2.6e-64) and aa 39-573 to Tle4 from *P. aeruginosa* (4R1D_A) and a phospholipase (4X91_C) and an acetyltransferase (4X96_B) from *Homo sapiens* with 100% confidence. Phylogenetic analysis also confirmed the grouping of Tle4b to Tle4a/TplE^PAO1^ (Supplementary Figure S12B).

*In silico* analysis of the downstream gene (e.g., *PALA8_00264*) confirmed its putative function as the Tle4b immunity protein which we refer to as Type VI lipase immunity family 4b (Tli4b). Protein domain analysis predicted the putative immunity to possess the Tli4 immunity protein N- (cl39779, E-value 1.45e-04) and C- (cl39763, E-value 2.38e-14) terminal domains. Structural homology searches using HHpred matched aa 3-390 to Tli4a (PA0259) and Tli5b from *P. aeruginosa* strain PAO1 (4R1D_B, 1.1e-55, Probability: 100%, and 5XMG_A, 6.6e-38, Probability: 100%). Immunities against T6SS lipases are commonly located in the periplasm to counter lipase activity (Jiang et al., 2014, 2016; Berni et al., 2019); however, our analysis could not identify a signal peptide (SignalP, Other: 0.9969), nor the characteristic lipobox motif (Zückert, 2014). Further, no transmembrane helices were predicted by TMHMM v2.0, while CELLO2GO predicted cytoplasmic subcellular localisation (Score: 4.149) which was unexpected.

In summary, these data suggest a putative *tle4-tli4* EI pair is encoded in select *P. aeruginosa* strains, containing a putative T6SS lipase effector family 4 protein with homology to previously described effector Tle4a/TplE^PAO1^ (Jiang et al., 2016). Due to the similarities noted at important active residues and characterised domains, we predict protein Tle4b to exhibit similar activity to Tle4a/TplE within the clinical effector-containing isolates.

#### Identification of one Metallopeptidase

**Figure 12:**
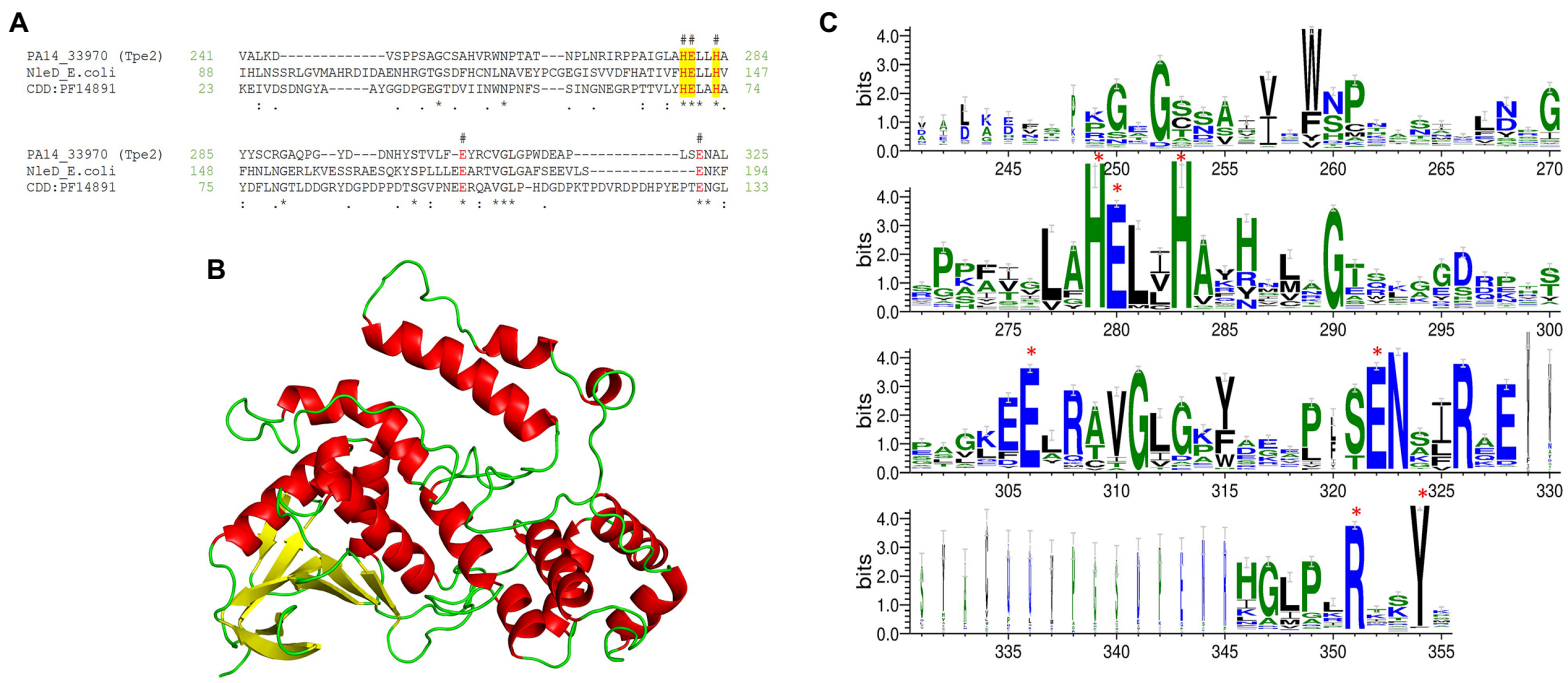
Tpe2 is a putative metallopeptidase. **(A)** Amino acid sequence alignment of Tpe2 (PA14_33970) from PA14, the characterised metallopeptidase NleD from *E. coli* (WP_247157463.1) and the consensus sequence of the peptidase M91 domain (NCBI-CDD domain PF14891) highlight the conservation of the metallopeptidase HExxH motif (highlighted yellow) and amino acids E261 and E350 conserved within BoNT neurotoxins as identified in Mansfield et al. (in red and labelled with #; Mansfield et al., 2019). **(B)** Alphafold (Jumper et al., 2021; Varadi et al., 2022) predicted model of Tpe2 visualised using PyMOL (The PyMOL Molecular Graphics System, Version 2.5.4, Schrödinger, LLC). **(C)** WebLogo3 diagram showing the most frequent residues found in homologs of Tpe2 from a PSSM approach. Conserved residues from the Botulinum neurotoxin metalloproteases family are labelled with a red star.

Our fifth new effector was observed within the H3-T6SS cluster of PA14 (PA14_33970) and 16 clinical isolates (Figure 3) we refer to it as Type VI peptidase effector family 2 (Tpe2). Domain analysis predicted Tpe2 to contain a CT peptidase M91 domain (PF14891, 7.77e-08) at aa 219-328 which is a domain harboured in the *E. coli* EPEC T3SS effector NleD (Baruch et al., 2011). NleD targets intracellular eukaryotic proteins JNK and p38, cleaving them and suppressing the inflammatory response via the zinc-dependent metallopeptidase HExxH motif (Baruch et al., 2011). Tpe2 alignment to *E. coli* NleD and the consensus sequence of domain PF14891 confirmed the presence of the HExxH motif in Tpe2 (Figure 12A). Phyre2 structural homology searches matched Tpe2 aa 245-311 to Botulinum neurotoxin (BoNT) type f (PDB: 2A97_B, Confidence: 94.5) and a *Clostridium* neurotoxin family metalloprotease (“zincins”; PDB: 1T3C_A, Confidence: 92.5; Figure 12B). A PSSM-methodology analysis of Tpe2 also revealed several conserved sequence features with Botulinum neurotoxin metalloproteases by possessing parallel residues to zinc ligand E261 and active site-refining residue E350 and RxxY motif (Figure 12C; Mansfield et al., 2019). In addition, PSSM search hits identified Tpe2 homologs were encoded in several bacterial genera including *Pseudomonas, Xanthomonas, Ralstonia, Escherichia,* and *Paraburkholderia.*

Several putative and characterised T6SS metallopeptidase effectors have been reported to date, including VgrG2b from *P. aeruginosa,* Fte1 from *Flavobacterium johnsoniae*, Rhs-MPTase from Shiga toxin-producing *E. coli,* TseH from *V. cholerae*, and two putative M23 metallopeptidase-containing proteins from *Paraburkholderia phymatum* (Russell et al., 2014; Altindis et al., 2015; Ma et al., 2017; Wood et al., 2019a; Hug et al., 2021). The M91 peptidase domain-containing protein (PA15_0330735) has been previously identified in *P. aeruginosa* strain HB15 (Mesquita et al., 2019), which was confirmed by our analysis to harbour an identical homolog to Tpe2 in location and sequence. The authors of the study did not characterise this protein but putatively labelled it as a predicted virulence factor analogous to our analysis. Similar to Tsd2 described above, Tpe2 was also predicted to have no cognate immunity protein and is present in 31% of our strains (Figure 5).

In summary, our analysis predicts that Tpe2 is an anti-eukaryotic effector, analogous to the neighbouring putative effector Tsd2, and possesses a putative metalloprotease activity (Figure 3). Moreover, to date the H3-T6SS cluster has only one secreted substrate, TseF, which acts as a metal ion scavenging protein (Lin et al., 2017; Wettstadt et al., 2019). From our data, we expand the putative repertoire of H3-T6SS secreted effectors through the identification of Tsd2 and Tpe2. Both predicted effectors appear to antagonistically target eukaryotes and support the hypothesis that the H3-T6SS facilitates anti-eukaryotic toxicity.

#### Identification of three putative T6SS Pyocins TspE1a/b/c

**Figure 13:**
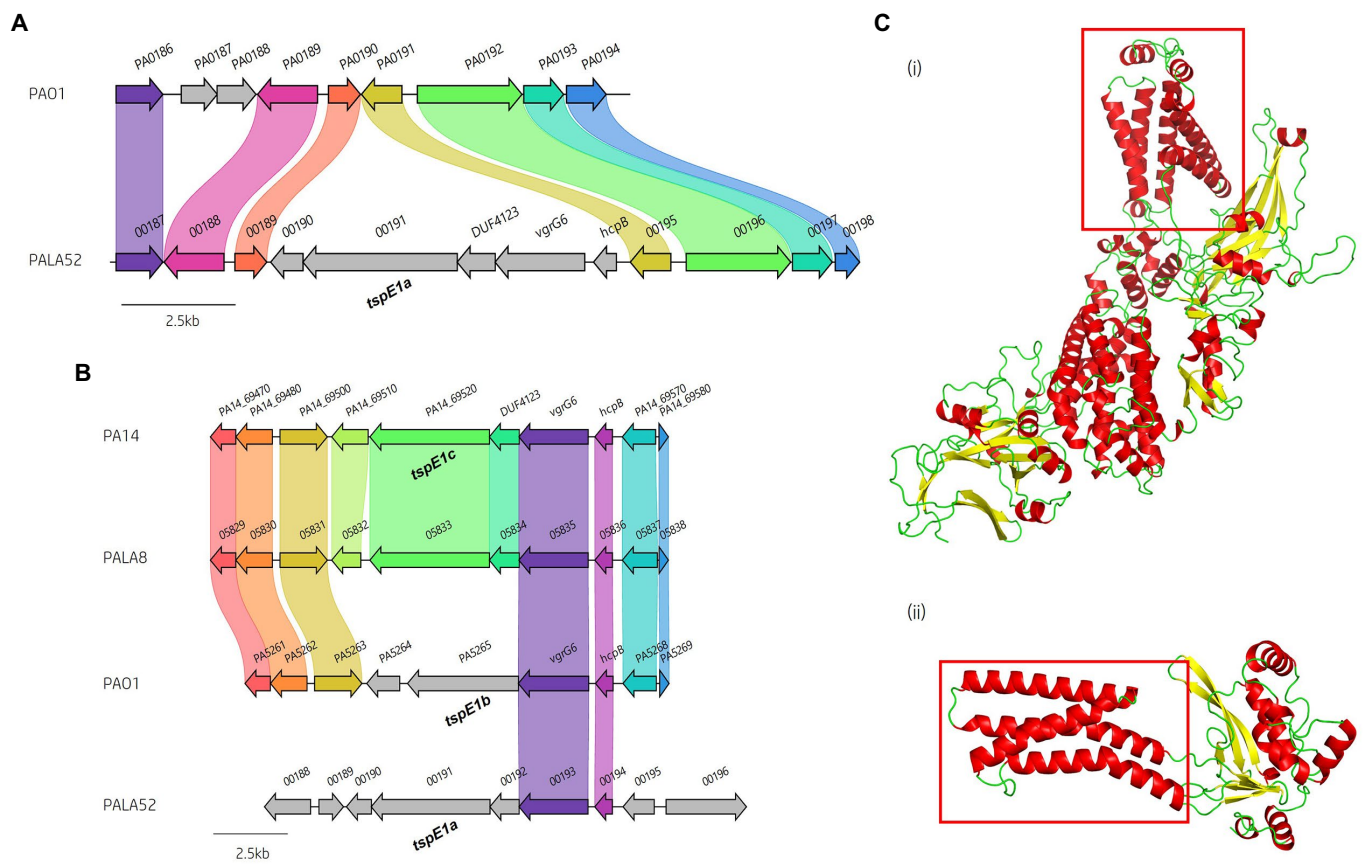
Genes encoding three different (*tsep1a/b/c*) putative T6SS secreted pyocin effectors are present in *vgrG6* islands from different isolates. **(A)** Visualisation showing the genomic location of the *vgrG6* island in PALA52 compared to PAO1. The *vgrG6* island genetic location in PAO1 and most strains is shown in panel B. **(B)** Comparative visualisation of *vgrG6* islands within clinical isolates to PAO1 and PA14 highlights the high conservation of *hcpB* and *vgrG6* but the strain variation of downstream genes. The *vgrG6* island from PAO1 encodes a putative pyocin effector *tspE1b* (PA5265). Isolate PALA8 is used as a representative for the possession of PA14 *vgrG6* island and EI module *tspE1c*/*tspI1c* (05833). The *vgrG6* island from PALA52 encodes a third putative pyocin-type effector *tspE1a* (00191). **(C)** Alphafold (Jumper et al., 2021; Varadi et al., 2022) prediction of (i) TspE1b (PA5265) and (ii) TspI1b (PA5264) from *P. aeruginosa* strain PAO1 visualised using PyMOL (The PyMOL Molecular Graphics System, Version 2.5.4, Schrödinger, LLC). The red box in (i) indicates the location of the pore-forming α-helical bundle, while the red box in (ii) indicates the four transmembrane helices of the putative immunity.

Orphan island *vgrG6* is the only *vgrG* island in *P. aeruginosa* to have no characterised cognate effectors associated to date. However, previous work shows that VgrG6 is required for secretion of effector TseT during antibacterial warfare (Burkinshaw et al., 2018). As VgrG6 groups with H2-T6SS VgrGs and HcpB’s sequence identity (encoded in the *vgrG6* island) to Hcp2, HcpA, and HcpC this suggests secretion *via* the H2-T6SS (Figure 13A; Supplementary Figure S8). Furthermore, ChIP-seq data has shown the *vgrG6-hcpB* cluster is co-regulated with the core H2-T6SS cluster *via* RpoN, and that RpoN can directly bind upstream of the *vgrG6* cluster promoter region, supporting the link with the H2-T6SS (Allsopp et al., 2022).

Comparative analysis of the *vgrG6* island across clinical isolates and reference strains PAO1 and PA14 revealed a level of diversity in neighbouring genes not previously observed. Three distinct EI modules were identified downstream of *vgrG6* genes (Figure 13). All three putative effectors encode a conserved N-terminus but divergent CT. The presence of the three EI modules was first observed through the *vgrG6* island insertion into a new locus in isolate PALA52, between homologs of *PA0190* and *PA0191* in PAO1 (Figure 13A). Unusually, the PALA52 *vgrG6* island variant encoded three genes downstream of *vgrG6,* whereas in PAO1 only two genes are encoded (Figure 13B). Further analysis revealed that PALA52 was unique in its possession of the *vgrG6* island at its genomic loci. However, strain PA14 and 13 clinical isolates (represented by PALA8) encode a homologous EI module that is highly conserved and likely shares the same function. Both variant *vgrG6* islands in PA14 and PALA52 encode a DUF4123 domain-containing protein immediately adjacent to *vgrG6.* DUF4123 domains are commonly found in T6SS chaperone proteins and are likely responsible for effector loading to their cognate secretion system (Liang et al., 2015; Unterweger et al., 2015).

Next, we predicted effector functions for each *vgrG6* island from PAO1, PA14 and PALA52. These three polymorphic toxins all contain pyocin-like CT and are therefore denoted as Type six pyocin Effector family 1a (TspE1a - PALA52_00191), 1b (TspE1b – PA5265), and 1c (TspE1c – PA14_69520). Putative effectors TspE1a and TspE1c were predicted to harbour a MIX_IV clan domain in their N-terminus (cd20708, E-value 2.46e-49 and 2.34e-44, respectively), whilst TspE1b was predicted to possess a MIX_I clan domain (cd20705, E-value 3.18e-31). Alignment of all TspE1 effectors and identification of the MIX motifs; shPhhR, hRxGhhYhh, and hhYSxxxWT confirmed this (Supplementary Figure S13A; Salomon et al., 2014).

VgrG6 and HcpB homologs encoded in the genome of PALA52 share 92 and 100% aa identity to their homologs in strain PAO1, respectively. In comparison, the aa identity of TspE1a to TspE1b and TspE1c is 18 and 21%, respectively. TpsE1a is a large predicted protein (~129.95 kDa) but possesses no identifiable CT domains. Therefore, we performed structural homology searches, where HHpred matched the TpsE1a-CT [aa741-944] to Toxin A (TcdA)/Glucosyl transferase from *Clostridium difficile* (4R04, Probability: 64.07) and to C-terminal pore-forming domains of Pyocin S5 (6THK, Probability: 46.58%) and Colicin S4 (3FEW, Probability: 34.90%). A congruent result was then seen with Phyre2, matching the aa 817-931 of TspE1a to Colicin S4 (3FEW, Confidence: 80.5, aa 376-483) and aa 842-919 to Pyocin S5 (6HTK, Confidence: 65.0, aa 405-482). Pyocin S5 is a bacteriocin that specifically targets *P. aeruginosa,* leading to cell death through pore formation (Ling et al., 2010; Elfarash et al., 2014; Behrens et al., 2020). Pyocin S5 and Colicin S4 CTs possess a 10-helical bundle pore-forming structure facilitating killing activity (Cascales et al., 2007; Arnold et al., 2009; Ling et al., 2010; Behrens et al., 2020). The HHpred aligned region of TspE1a [aa 741-944] was predicted to contain 7 α-helices using PSIPred; however, a larger region [aa 711-974] contained 9 predicted α-helices. It is therefore likely that *tspE1a* is a pore-forming effector.

The toxicity of Pyocin S5 is counteracted by a small membrane localised immunity protein, ImS5, preventing membrane depolarisation by the Pyocin S5 pore-forming domain (Ling et al., 2010). ImS5 possesses three transmembrane helices that allow it to sit in the periplasm and categorise it as part of the immunity proteins against E1-Type Colicins (Cascales et al., 2007; Behrens et al., 2020). Here, we predicted a small (~27.63 kDa) cognate immunity protein for TpsE1a, which we denote as Type six pyocin immunity family 1a (TspI1a), that possesses four predicted transmembrane helices (Supplementary Figure S13B) that categorises it into immunity proteins against A-Type Colicins. CELLO2GO predicted subcellular translocation to the inner membrane (Score: 5.962) which putatively fits with the site of action of its effector TspE1a.

We then analysed TpsE1b (PA5265) from PAO1, predicting a pore-forming domain in its CT [aa 644-833] through HHpred structural homology searches which matched to Toxin B/TcdB from *C. difficile* (6AR6_A, Probability: 66.35) and overlapped the toxin translocation region that possesses pore-forming activity (Albesa-Jové et al., 2010). Phyre2 analysis matched aa 757-840 to Colicin IA (7KDP, Confidence: 69.5, aa 535-619) and aa 759-840 to Pyocin S5 (6THK, Confidence, 48.9, aa 408-490). A highly accurate TpsE1b (PA5265) model was available from the Alphafold database (Jumper et al., 2021; Varadi et al., 2022) and shown in Figure 13Ci supporting the pore-forming activity. Modelling of the cognate immunity protein to TpsE1b (PA5264), denoted TspI1b, predicted four transmembrane helices (Supplementary Figure S13C). TspI1b is marginally larger (~37.90 kDa) than TspI1a from PALA52 and supports the evidence that TpsE1b is a T6SS delivered pyocin type effector (Figure 13Cii).

Finally, TspE1c (PA14_69520) from PA14 also encodes a predicted pore-forming CT (Figure 13C). Structural homology searches with HHpred matched TspE1c [aa 864-974] to Pyocin S5 (6THK, Probability: 32.48%) and the pore-forming domain of Colicin A (1COL, Probability: 37.98%), whilst Phyre2 matched to Pyocin S5 (Confidence 96.0, PDB: 6THK_A). The TspE1c region overlapping with Pyocin S5 was predicted to harbour 7 α-helices, but 10 were predicted in aa 730-971. The putative cognate immunity protein to TspE1c, denoted as TspI1c, was predicted to possess two transmembrane helices but did not fall into an immunity protein category against colicins. Clinical isolate PALA8 was also predicted to encode EI pair *tspE1c-tspI1c* and DUF4123 protein-encoding gene from PA14 (Figure 13B). The putative TspE1c effectors PA14_69520 and PALA8_05833 share 99% aa identity, but putative TspI1c immunities PA14_69510 and PALA8_05832 only share 79% aa identity. This lower identity was caused by an N-terminal truncation of the immunity protein in isolate PALA8 leading to the presence of only one transmembrane helix.

Thus, three new putative effector proteins with pore-forming activities at their CT have been identified to be encoded within the *vgrG6* island of reference strains PAO1 and PA14, as well as clinical *Pseudomonas aeruginosa* isolates. Distinct CT sequences implicate these putative T6SS effectors in inter-strain competition, likely targeting other *P. aeruginosa* strains, functioning similarly to bacteriocin Pyocin S5 (Ling et al., 2010). Furthermore, each putative effector possesses a unique immunity protein, two of which encode a neighbouring chaperone protein (DUF4123) which could assist in effector loading to their cognate VgrG6. Finally, the presence of a *hcp* gene, the homology of the VgrG6 proteins with those of the H2-T6SS VgrGs, and co-regulation support that these newly identified substrates belong to the H2-T6SS effector arsenal.

### Prevalence of T6SS effectors in publicly available *Pseudomonas aeruginosa* genomes

**Figure 14:**
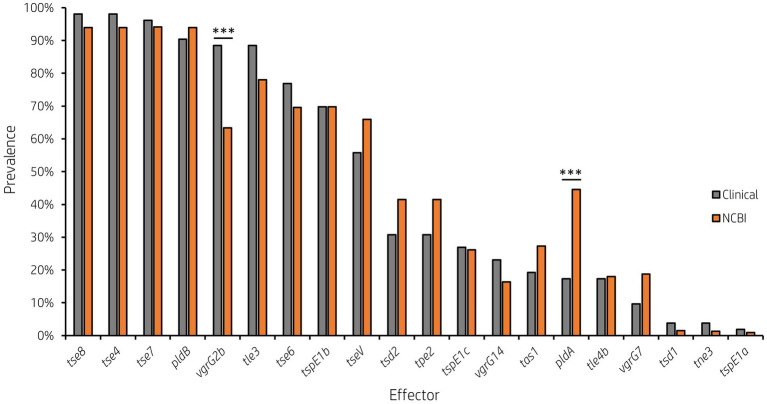
Prevalence of genes encoding T6SS effectors is variable across all publicly available *P. aeruginosa* isolates of assembly level ‘complete’ or higher. Grey bars represent the 52 clinical *P. aeruginosa* genomes under study and orange bars represent the 532 publicly available NCBI RefSeq *P. aeruginosa* genomes (*** *p* < 0.001, χ^2^ test).

After determining the prevalence of the 12 characterised and eight putative T6SS effectors identified in this work within our dataset of clinical *P. aeruginosa* isolates (Figure 5), we then performed a comparative prevalence study on 532 ‘complete’ *P. aeruginosa* genomes from the NCBI RefSeq using a BLAST screening method (File S3). Effector prevalence in our clinical isolates and the publicly available NCBI RefSeq *P. aeruginosa* genomes were compared (Figure 14). Only two effectors, *vgrG2b* and *pldA*, possessed a statistically significant difference in prevalence between the two *P. aeruginosa* groups (χ^2^, *p* < 0.001), suggesting potential enrichment of effectors within distinct populations of *P. aeruginosa* strains. As *vgrG2b* prevalence was significantly higher in CF isolates it could be selected amongst our clinical isolate population compared to the publicly available genomes, whereas *pldA* prevalence was significantly lower, suggesting it may be selected against in CF isolates. All eight putative T6SS effectors described in this study were found in at least five publicly available *P. aeruginosa* genomes, confirming that they are not exclusive to clinical isolates and are potentially utilised by other *P. aeruginosa* populations (Figure 14; Supplementary Tables S3, S6). In addition to these data, we show that certain effectors are either favoured or retained in *P. aeruginosa* isolates, suggesting its T6SS effectors could be grouped into either core or accessory. Core effectors (*tle4a, tse1, modA, tseF, tse5, tse2, tse3, tseT,* and *azu*) found in all strains would provide an analogous function and remain encoded in the genomes regardless of the selection conditions. Alternatively, accessory effectors (listed in Figure 5) would vary in prevalence based on population, indicating strain-specific advantages dependent on the environment. Our observation of T6SS component distribution in the core and accessory genomes of the clinical isolates under study supports this and is in line with a recent manuscript (Habich et al., 2022). Conservation of effector subsets, therefore, appears dependent on distinct selection pressures, driving their evolution towards a state with increased fitness and maximum effectiveness of the effectors they utilise.

## Conclusion

This study expands the current knowledge of the *P. aeruginosa* T6SS, particularly within clinical isolates from people with CF. We have determined the pangenome of the clinical *P. aeruginosa* isolates is ‘open’ and has an accessory genome enriched for HGT components and fitness-promoting genes. Several T6SS components were observed in the accessory genome, necessitating further investigation into their prevalence. Our analysis noted diversity at genomic loci encoding T6SS components in both clinical and publicly available genomes. Further, in-depth bioinformatics and protein function analysis predicted eight putative T6SS effectors encoding distinct functions that putatively target prokaryotes and/or eukaryotes (Figure 1). We also identify the *vgrG7* island in *P. aeruginosa* strain PAK and support the previous work suggesting that Tle2 is a lipase family effector. The major limitation of this study is that our analysis is purely *in silico* and limited to “inferred from sequence” or structural similarity. However, our rigorous analysis integrates multiple sources of evidence to predict functions, and in some instances identifies likely catalytic residues, of eight putative T6SS effectors in *P. aeruginosa* genomes, providing a foundation for future experimental characterisation. The *P. aeruginosa* T6SS genes appear to be under evolutionary pressures driving the selection of components for core and accessory functions. These roles appear dependent on the environmental factors required for pulmonary colonisation to distinct lung microenvironments encountered by each *P. aeruginosa* isolate. It is therefore conceivable that *P. aeruginosa* may use these accessory components, including the T6SS and the *vgrG2b* island for example, to establish dominance during CF infection by out-competing the commensal lung microbiome and other *P. aeruginosa* isolates as well as modulating the host through strain-specific fitness-promoting factors. Whilst T6SS components in the core may be more beneficial generally in a wider variety of environmental niches and hence harboured in a greater abundance of strains including those found in the CF lung.

## Data availability statement

The data presented in this study can be found in the NCBI repository. The accession numbers can be found in Supplementary Table S1.

## Author contributions

LA and LR conceived the study. LR conducted the bioinformatic analysis and analysed the data. LA managed the study. JD and RM established and managed the CF bacterial strain collection. LR, AC, JD, RM and LA drafted the manuscript. All authors contributed to data interpretation. All authors contributed to the article and approved the submitted version.

## Funding

This project was supported by the Academy of Medical Sciences/the Wellcome Trust/the Government Department of Business, Energy and Industrial Strategy/the British Heart Foundation/Diabetes UK Springboard Award [SBF006\1,161]. LA is also supported by a Research Grant 2020 from the European Society of Clinical Microbiology and Infectious Diseases as well as funds from the National Heart and Lung Institute at Imperial College London. LR is a recipient of a PhD studentship funded by the Academy of Medical Sciences and the National Heart and Lung Institute. AC is a recipient of a PhD studentship from the National Heart and Lung Institute. The PAPA sample repository was funded by the Cystic Fibrosis Trust through two Strategic Research Centre awards. JD and LA are supported by the NIHR through the Imperial Biomedical Research Centre, the Royal Brompton Clinical Research Facility and a Senior Investigator Award (JD).

## Conflict of interest

The authors declare that the research was conducted in the absence of any commercial or financial relationships that could be construed as a potential conflict of interest.

## Publisher’s note

All claims expressed in this article are solely those of the authors and do not necessarily represent those of their affiliated organizations, or those of the publisher, the editors and the reviewers. Any product that may be evaluated in this article, or claim that may be made by its manufacturer, is not guaranteed or endorsed by the publisher.

## Footnotes

^1^ http://www.microbesng.com

^2^ Genome List-Genome-NCBI [Internet]. *Bethesda (MD): National Library of Medicine (US), National Center for Biotechnology Information; 2004 - [cited 2020 Oct 01]*. Available at: https://www.ncbi.nlm.nih.gov/genome/browse#!/overview/ (Accessed October 1, 2020).

^3^ https://github.com/1h3/seqtk

^4^ https://www.bioinformatics.org/sms2/reference.html

^5^Home-Protein-NCBI [Internet]. *Bethesda (MD): National Library of Medicine (US), National Center for Biotechnology Information; 2004 - [cited 2020 Oct 01]*. Available at: https://www.ncbi.nlm.nih.gov/protein (Accessed October 1, 2020).

^6^ www.Pseudomonas.com

^7^ https://github.com/RoyChaudhuri/bioperl-scripts/blob/master/gb2fasta

^8^ https://web.expasy.org/compute_pi/
