# Supplementary material for: Diversity and prevalence of type VI secretion system effectors in clinical Pseudomonas aeruginosa isolates
Source: Front Microbiol. 2023 Jan 4;13:1042505. doi: 10.3389/fmicb.2022.1042505 (PMC9846239; doi:10.3389/fmicb.2022.1042505)
Supplement: Supplementary file 1 [file Data_Sheet_1.zip › Supplementary Materials, Figures S2-S13.DOCX]

***Supplementary Material***

**Supplementary Figures**

(B)


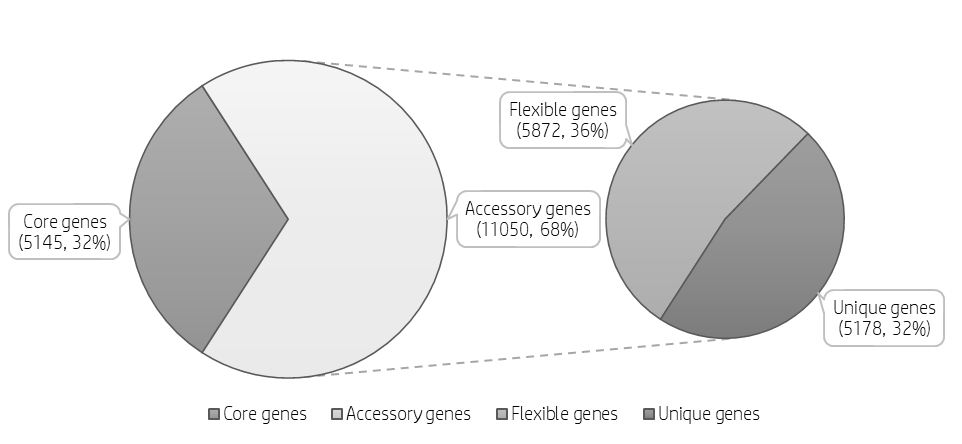


(A)


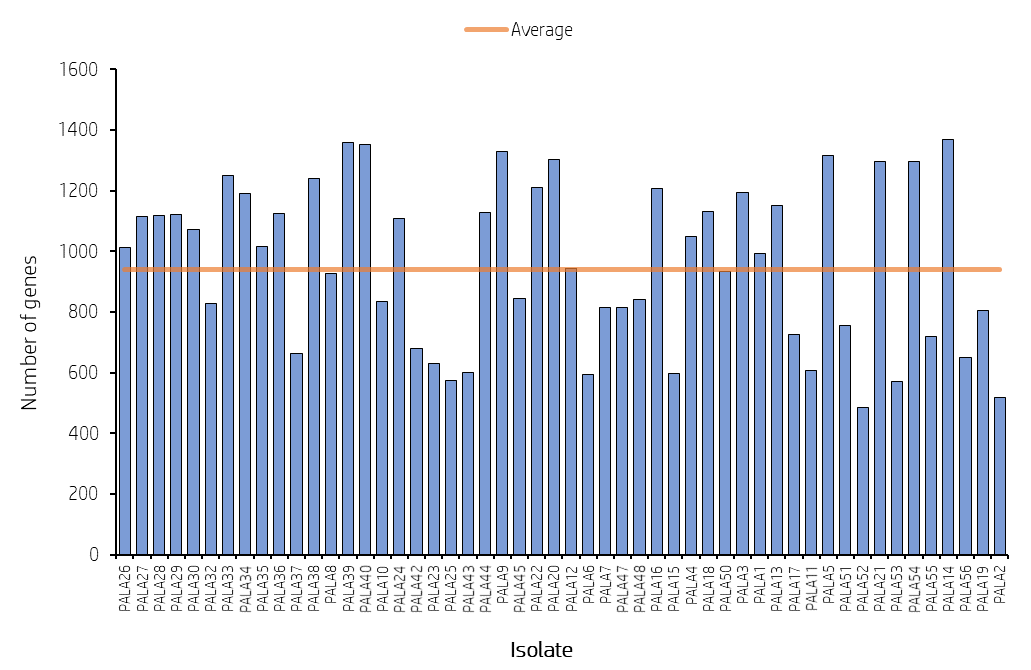


**Figure S2.** Pangenome analysis shows a large accessory genome. (**A**) Pie chart showing the distribution of core and accessory (flexible and unique) genes within the investigated *P. aeruginosa* isolates population pangenome. (**B**) Graph showing the number of accessory genes per genome of the isolates under study. The orange line is the average number of accessory genes across all genomes.


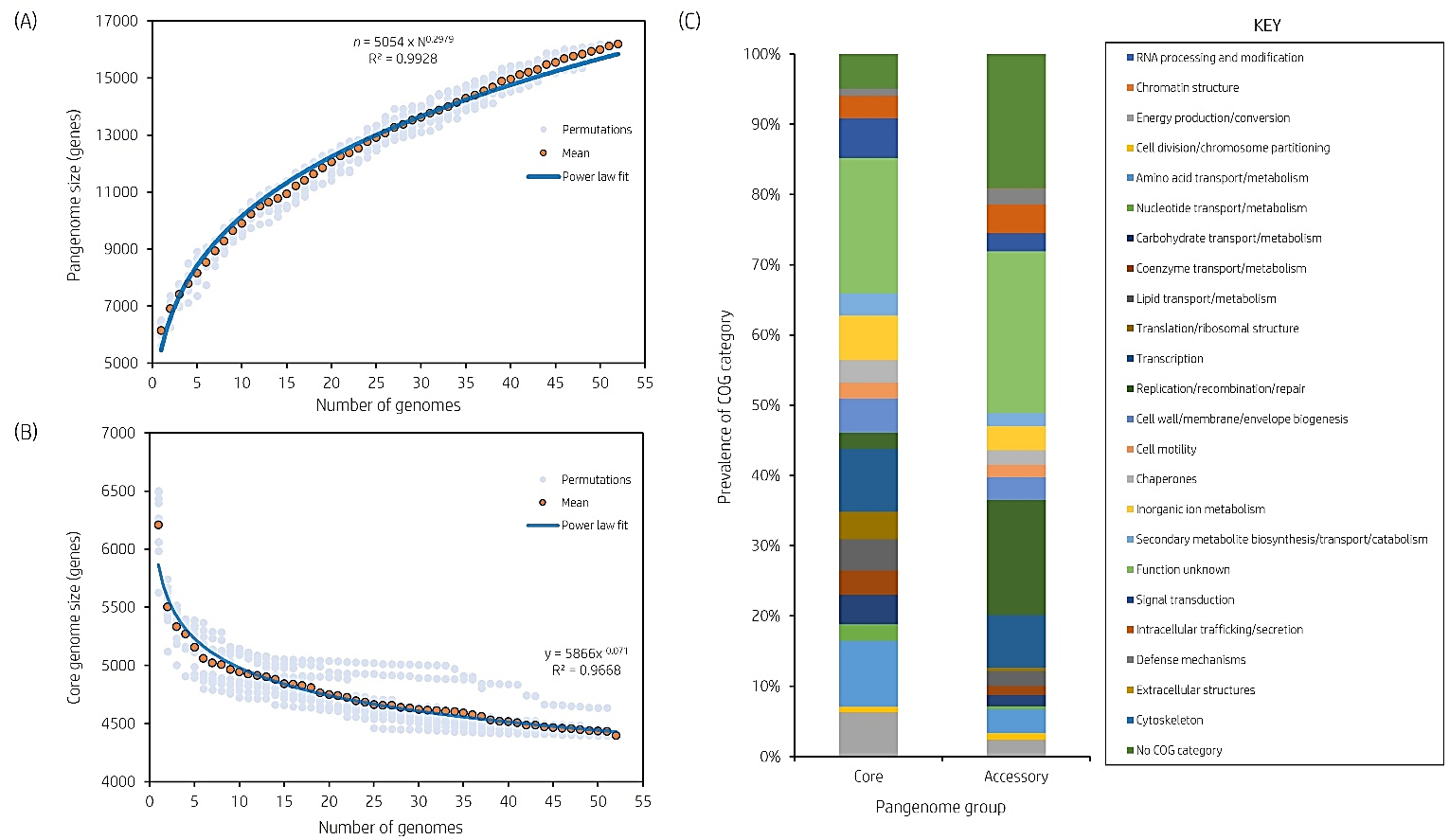


**Figure S3.** The clinical strains of *P. aeruginosa* have an ‘open’ pangenome receptive to genetic exchange and expansion. Gene plots of the pan- (**A**) and core-genomes (**B**) for the 52 *P. aeruginosa* isolates. Light blue circles represent 1 of 10 permutations at each sequential genome addition. Orange circles indicate the mean gene size of the permutations. Dark blue curves represent a power-law fit model of the mean gene size. The equation of the power-law fit and the coefficient of determination (R^2^) are shown. Exponent α was 0.702 ± 0.065 indicating an ‘open’ pangenome (γ = 0.2979), supported by the increasing pangenome size and decrease core genome size after each addition of a genome. **(C**) Genome-scale analysis of gene functions using COG shows variation in gene family presence in the core and accessory genomes. Percentage (%) of each COG category towards the total number of genes in the core and accessory genomes of the pangenome is indicated. COG categories for genes belonging to both groups were predicted using eggNOG-mapper v5.0.

2.5kb

PA14

PALA8

PAO1


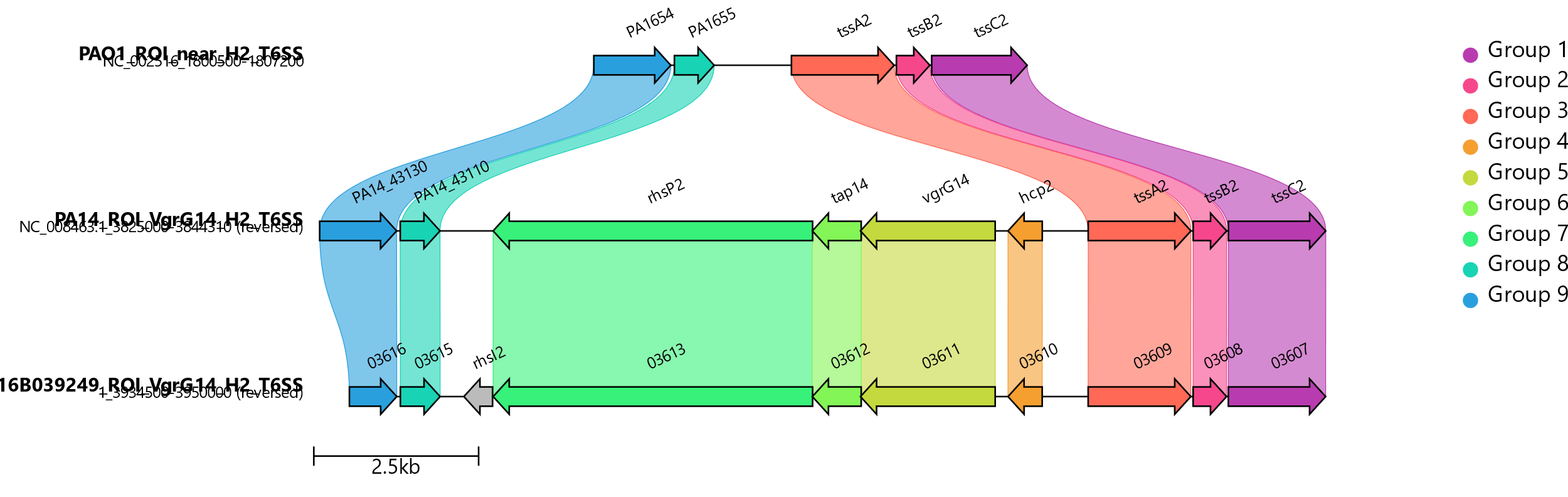


**Figure S4.** Comparative visualisation of the *vgrG14* island insertion in clinical isolate PALA8 and reference strain PA14 compared to PAO1. Genes coloured the same with connected bands are considered homologs. Genes found in only one genome possess a grey arrow. Gene sizes are to scale. The figure was generated using Clinker (Gilchrist and Chooi, 2021).

2.5kb

PALA9

PAO1

(A)

(B)


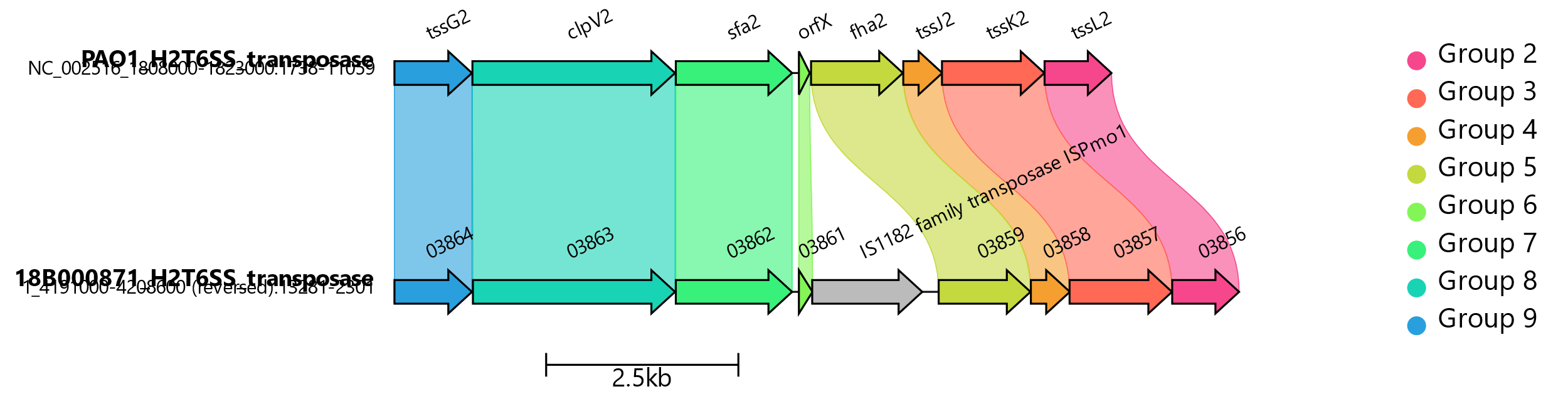


**Figure S5.** Transposase insertion into the H2-T6SS cluster. (**A**) Comparative visualisation of a transposase insertion within the H2-T6SS cluster of clinical isolate PALA9 compared to PAO1. (**B**) Amino acid sequence alignment of OrfX (PA1664) from PAO1 and homolog from the genome of PALA9 showing site of integration which likely disrupts OrfX. An asterisk (*) indicates fully conserved residues, a colon (:) indicates a strongly similar property at the residue, and a period (.) indicates a weakly similar property at the residue. Residue positions are given on the right-hand side of the alignment for each sequence, and the sequences identifier is given on the left-hand side of the sequence. The alignment was created using ClustalOmega (EMBL-EBI) (Madeira *et al.,* 2022).

PALA11

PAO1

PALA21

2.5kb


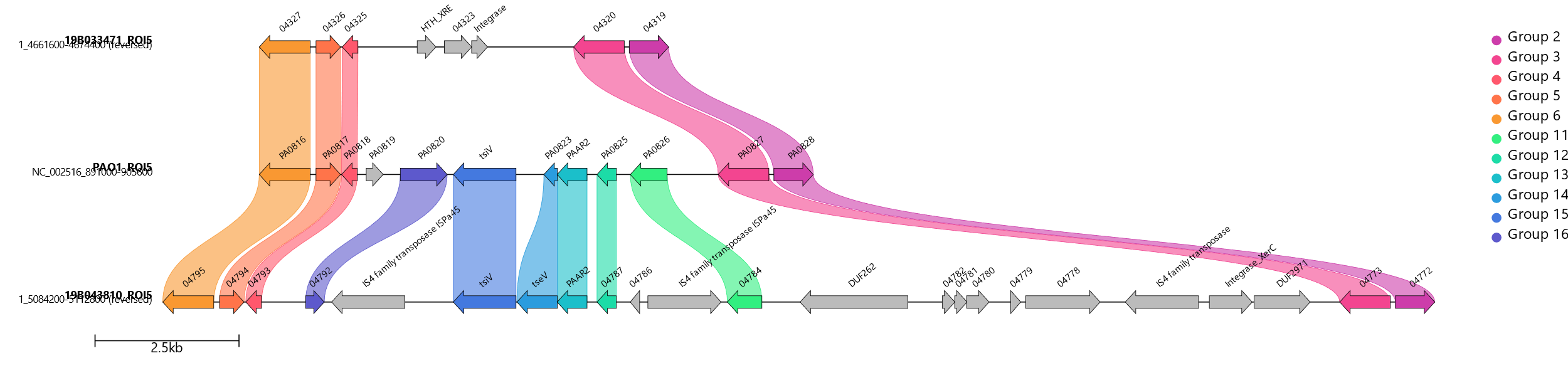


**Figure S6.** The *PAAR2* island has been subjected to transposon integration. Comparative visualisation of the *PAAR2* island encoded within the genomes of clinical isolates to PAO1. Isolate PALA11 is used as a representative for the insertion of genetic elements into the genetic location where *PAAR2* would be. Isolate PALA21 possesses the insertion of ISPa45 transposases on either side of the *PAAR2* island.

2.5kb

PAO1

PALA37


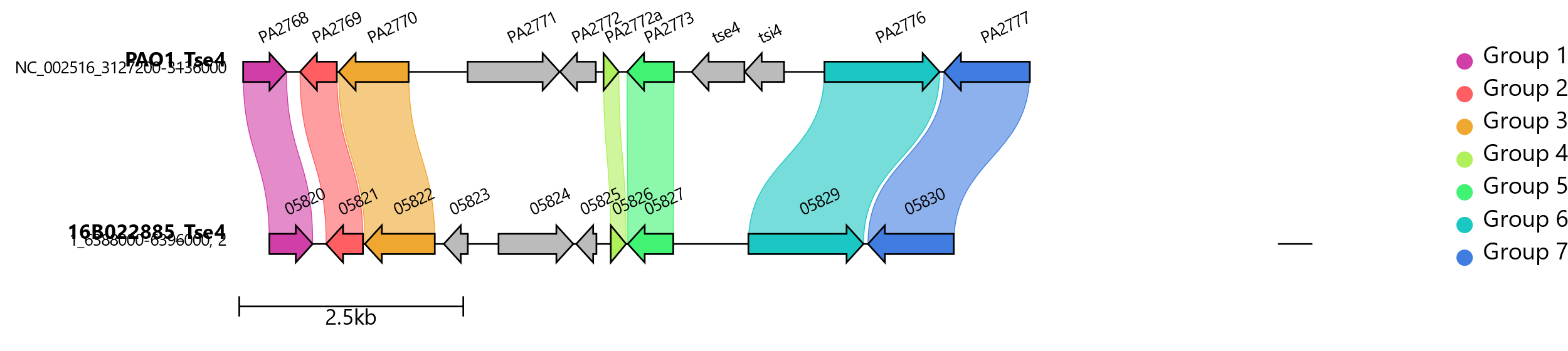


**Figure S7.** Comparative visualisation of the *tse4*-*tsi4* pair encoded within the genomes of clinical isolates to reference strain PAO1 displaying an example in the variation of effector complement.

**
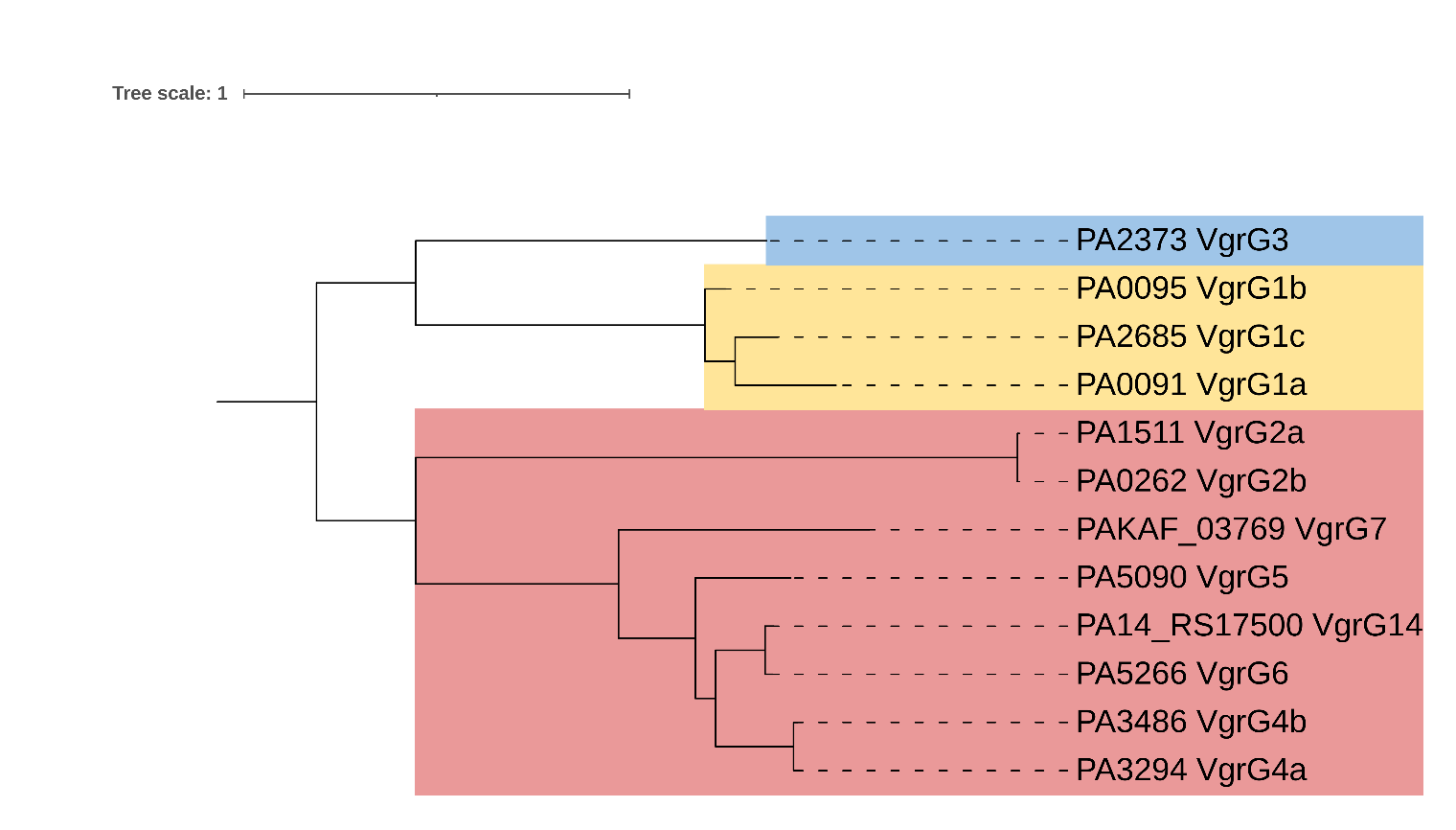
**

**Figure S8.** VgrG7 is likely secreted by the H2-T6SS as it clades with H2-T6SS associated VgrG proteins. Phylogenetic tree derived from a Maximum likelihood analysis on a ClustalW alignment of amino acid sequences of VgrGs from *P. aeruginosa* PAO1 (PA); PA14 (PA14_RS); and PAK (PAKAF). H1-T6SS VgrGs are coloured yellow, H2-T6SS VgrGs are coloured red, and H3-T6SS VgrGs are coloured blue. Phylogenetic analysis was conducted in MEGAX using the JTT model + Frequencies, G (n=5), partial deletion (95%), and NNI ML heuristic model parameters. The tree was visualised using the iTOL (v6) webserver (Letunic and Bork, 2021) and rooted at the midpoint. The scale bar indicates the genetic distance.

**
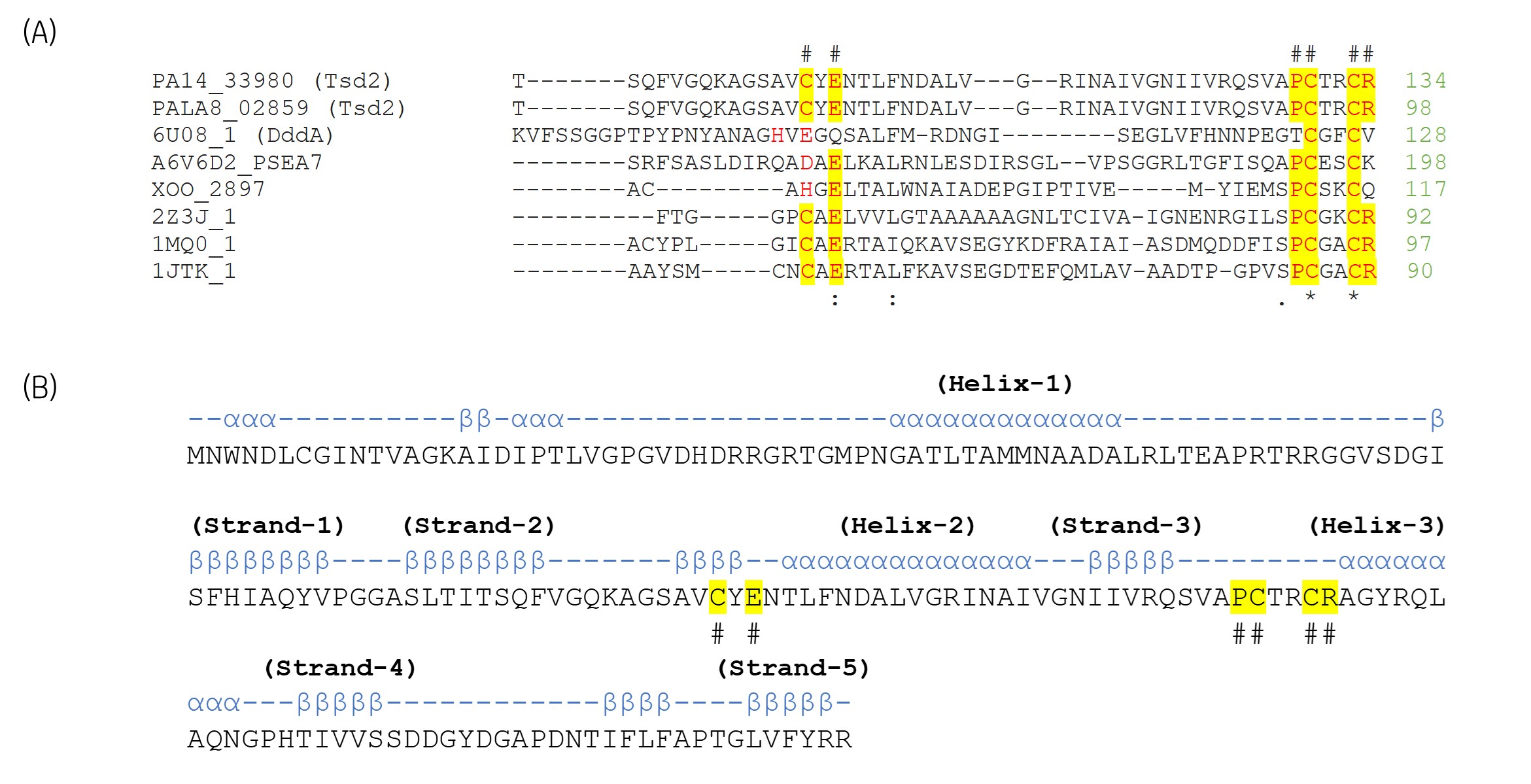
**

**Figure S9.** The H3-T6SS cluster in PA14 and PALA8 contains *tsd2* that encodes a predicted cytidine deaminase effector. (**A**) Targeted amino acid alignment of PA14_33980 and PALA8_02859 to deaminases DddA from *Burkholderia cenocepacia* (PDB: 6U08_1), A6V6D2_PSEA7 from PA7 (WP_012076009.1), XOO_2897 from *Xanthomonas oryzae pv. oryzae* MAFF311018 (WP_011408952.1), blasticidin S deaminase from *Aspergillus terreus* (PDB: 2Z3J_1), Human Cytidine Deaminase from *Homo sapiens* (PDB: 1MQ0_1), and cytidine deaminase from *Bacillus subtilis* (PDB: 1JTK_1). Residues of the CxE and PCxxCR motifs are highlighted yellow and indicated by an #. Key cytidine deaminase residues are coloured red. (**B**) Secondary structure prediction of Tsd2 with the important helices and strands described by Iyer et al. labelled and CxE and PCxxCR motifs highlighted yellow (Iyer *et al.,* 2011).


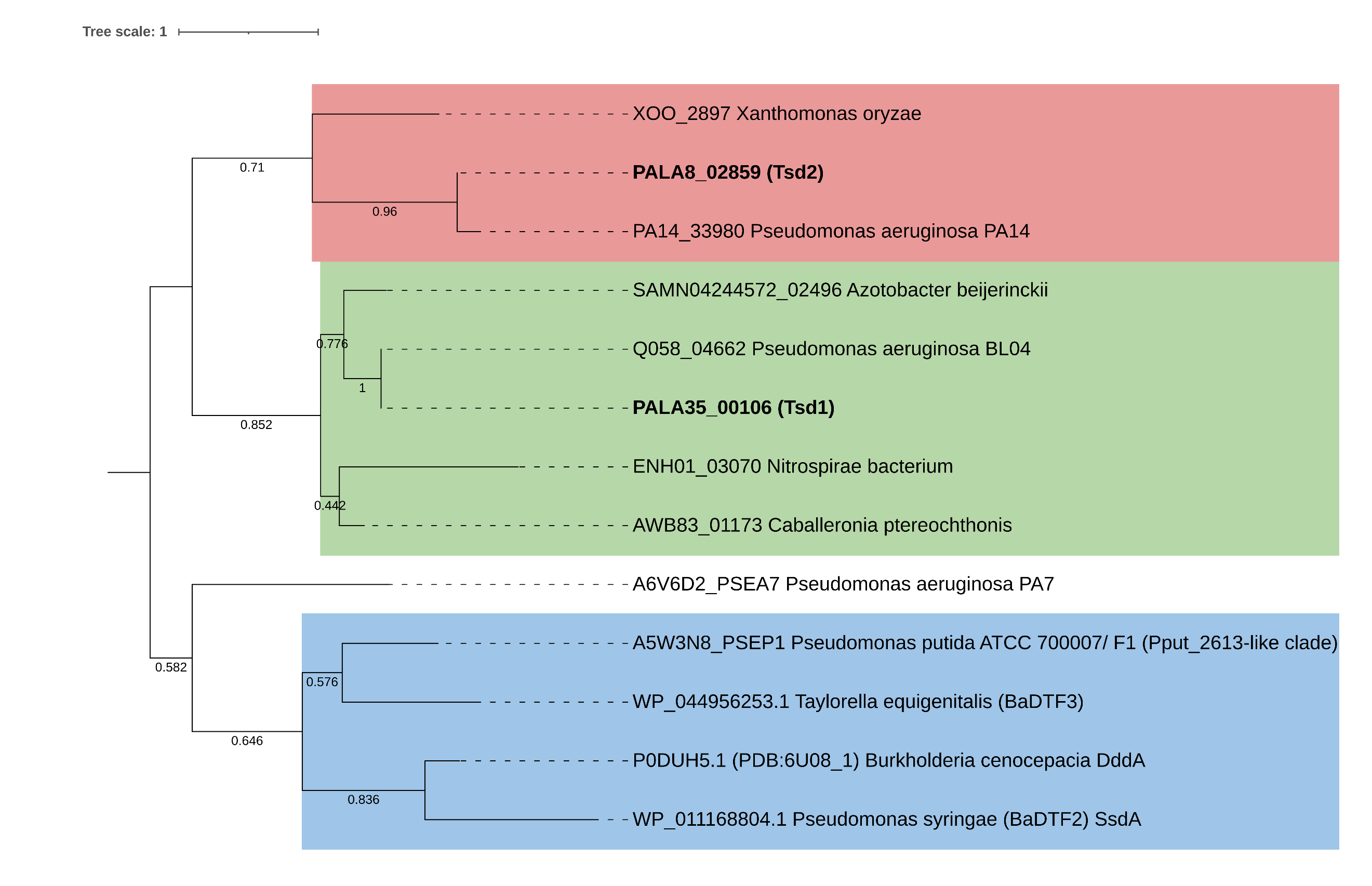
**Figure S10.** Tsd1 and Tsd2 claded into two different cytidine deaminase activity families. Maximum likelihood phylogenetic tree of amino acid sequences from Tsd1 (PALA35_00106), Tsd2 (PA14_33980) from *P. aeruginosa* UCBPP-PA14 and homolog, PALA8_02859, XOO_2897 from *Xanthomonas oryzae* (WP_011408952.1), SAMN04244572_02496 from *Azotobacter beijerinckii* (SEJ03043.1), AWB83_01173 from *Caballeronia ptereochthonis* (SAK50766.1), ENH01_03070 from *Nitrospirae bacterium* (HDH04682.1), Q058_04662 from *P. aeruginosa* BL04 (ERV74086.1), A6V6D2_PSEA7 from *P. aeruginosa* PA7 (WP_012076009.1), A5W3N8_PSEP1/Pput_2613 from *Pseudomonas putida* ATCC 700007 (ABQ78748.1) – a member of the clade Pput_2613-like (Iyer *et al.,* 2011), WP_044956253.1 from *Tayorella equigenitalis* – a member of the group BaDTF3 (de Moraes *et al.,* 2021), DddA from *Burkholderia cenocepacia* (PDB: 6U08_1) (Mok *et al.,* 2020), and SsdA from *Pseudomonas syringae* (WP_011168804.1) – a member of group BaDTF2 (de Moraes *et al.,* 2021). Phylogenetic analysis was conducted in MEGAX using the WAG model, G+I (n=5), bootstrap n = 500, partial deletion (95%), and NNI ML heuristic model parameters. The protein locus_tag, accession number, or protein identifier is given at each label, as well as species and strain if given. The tree was visualised using the iTOL (v6) webserver (Letunic and Bork, 2021) and rooted at the midpoint. The scale bar indicates the genetic distance. Bootstrap values are given at the respective nodes. BaDTF = Bacterial Deaminase Toxin Family.


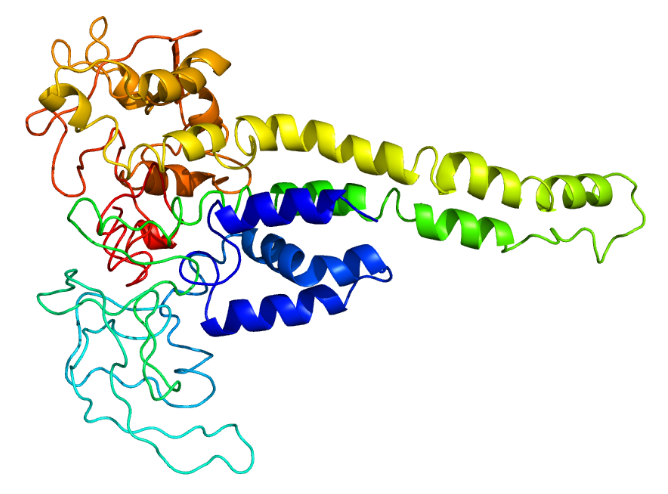

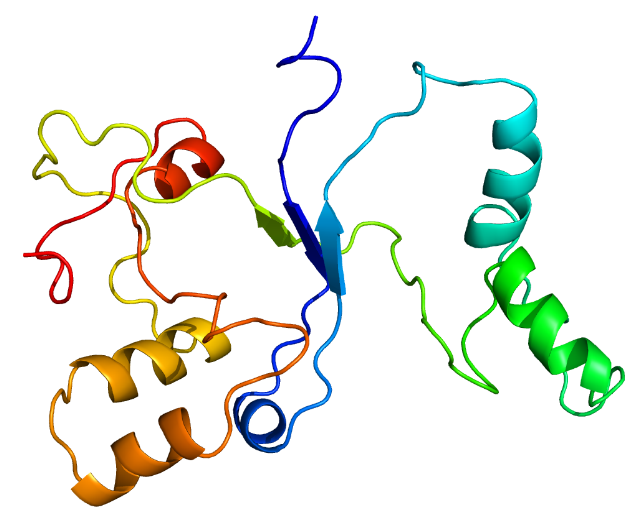


(A)

(B)

**Figure S11.** *In silico* analysis of Tne3 reveals predicted NADase activity. Phyre2 model of (**A**) Tne3 and (**B**) Tni3. The red box highlights the C-terminal structural homology to NAD^+^ glycohydrolase activity-containing proteins.

(B)

(A)


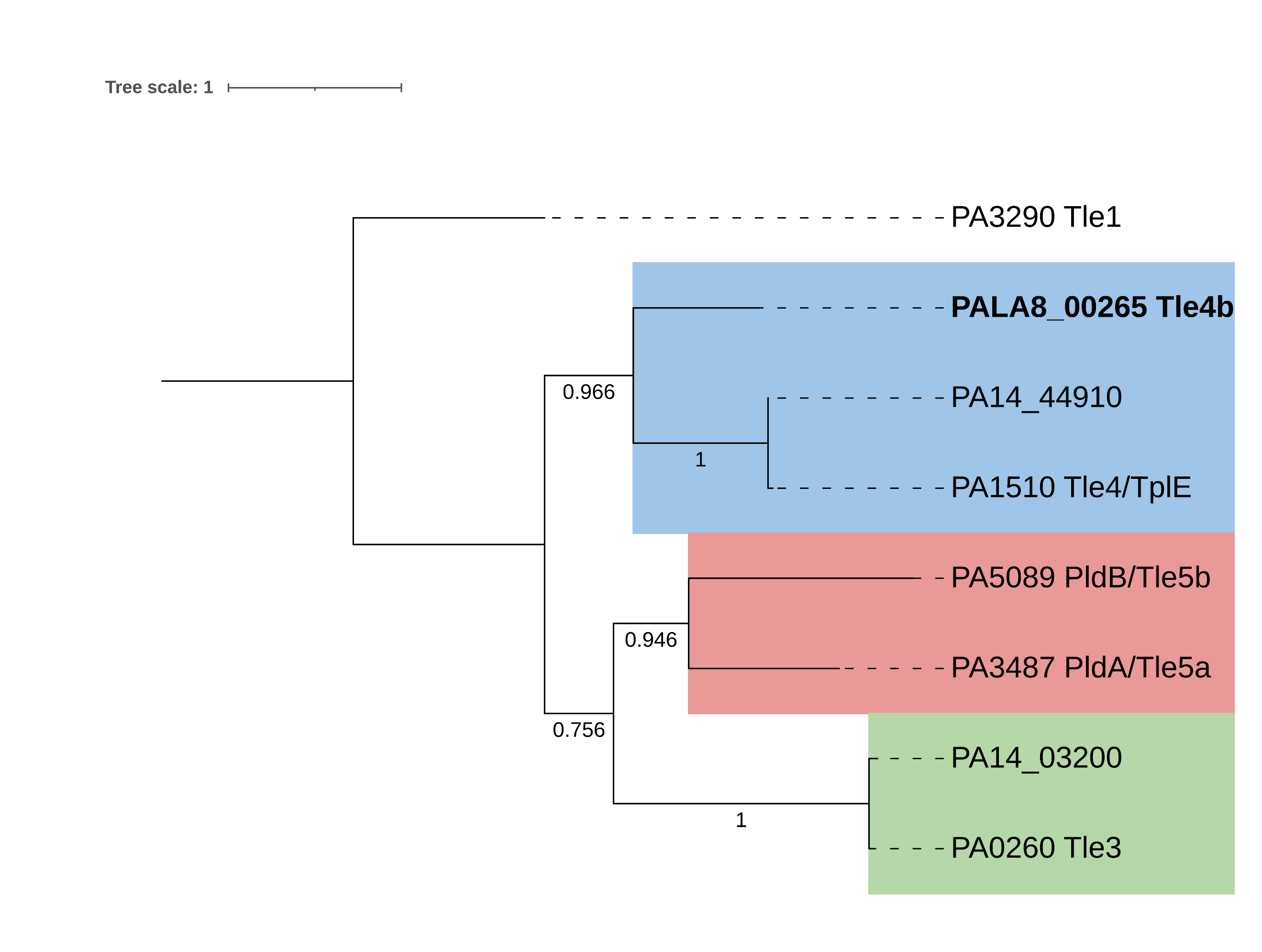


**Figue S12.** Tle4b (PALA8_00265) belongs to the lipase effector family 4. (**A**) Partial amino acid sequence alignment of Tle4b from isolate PALA8 (00265), Tle3 (PA0260) and Tle4a/TplE from PAO1 (PA1510) showing the lipase catalytic triad Ser-Asp-His highlighted in yellow and indicated by an #. Residues of the G/TxSxG motif are coloured red (Russell *et al.,* 2013). Residues of the PGAP1-like domain (PF07819) seen in TplE/Tle4^PAO1^ are coloured blue (Jiang *et al.,* 2016). The alignment was created using ClustalOmega (EMBL-EBI). (**B**) Phylogenetic tree derived from a Maximum likelihood analysis on a MUSCLE alignment of amino acid sequences from Tle4b, *P. aeruginosa* PAO1 proteins Tle1, Tle4a/TplE, PldA/Tle5a, PldB/Tle5b, and Tle3, and *P. aeruginosa* PA14 proteins Tle4a/TplE and Tle3. Phylogenetic analysis was conducted in MEGAX using the WAG model, G+I (n=5), bootstrap n = 500, partial deletion (95%), and NNI ML heuristic model parameters. The tree was visualised using the iTOL (v6) webserver and rooted at the midpoint. The scale bar indicates the genetic distance. Bootstrap values are given at the respective nodes.

(A)

**
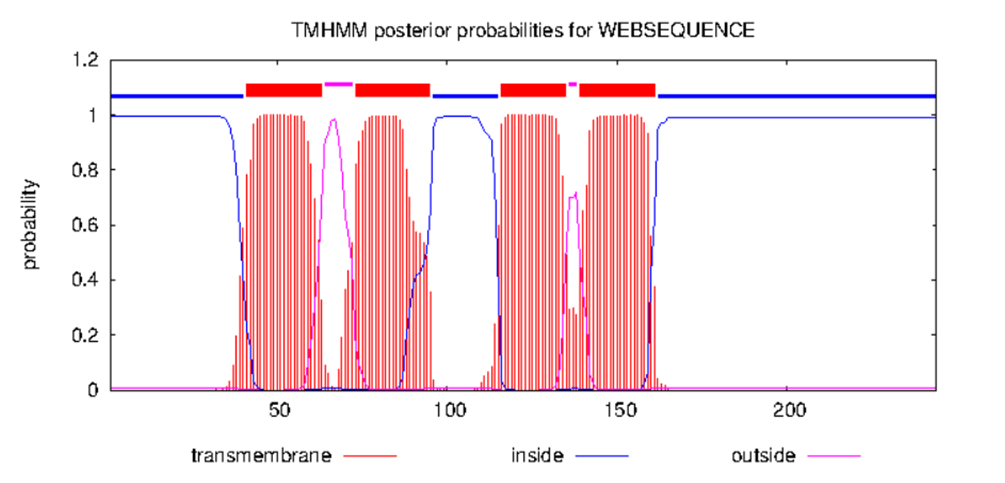
**

(B)

**
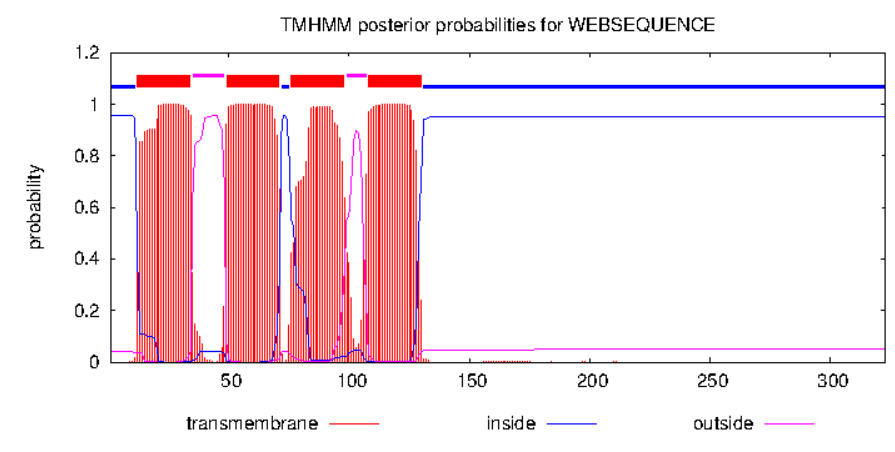
**

(C)

**Figure S13**. (**A**) Amino acid sequence alignment of TspE1a, (PALA52_00191), TspE1b (PAO1 PA5265), and TspE1c (PALA8_05833) show the T6SS MIX motifs highlighted in green and described by Salomon et al. (Salomon *et al.,* 2014). TMHMM v2.0 (Sonnhammer et al., 1998) analysis shows that TspI1a (**B**) and TspI1b (**C**) have four putative transmembrane helices and thus would likely be located within the membrane.
