# Supplementary material for: Diversity and prevalence of type VI secretion system effectors in clinical Pseudomonas aeruginosa isolates
Source: Front Microbiol. 2023 Jan 4;13:1042505. doi: 10.3389/fmicb.2022.1042505 (PMC9846239; doi:10.3389/fmicb.2022.1042505)
Supplement: Supplementary file 1 [file Data_Sheet_1.zip › Supplementary Materials, Tables S1-S2, S4, S6.docx]

***Supplementary Material***

**Supplementary Tables**

**Table S1.** Genome characteristics and descriptions of the 57 *P. aeruginosa* clinical isolates used in this study and reference strains PAO1, PA14, and PAK.

| Strain ID | Patient ID | Genome Size (bp) | Year of isolation | Source of isolation | Contigs | CDS | G+C% | tRNAs | N(50) | Sequence Type | Genome Accession ID |
| --- | --- | --- | --- | --- | --- | --- | --- | --- | --- | --- | --- |
| PAO1 |  | 6264404 |  |  |  | 5572 | 66.56 | 63 |  | 549 | NC_002516.2/ AE004091.2 |
| PA14 |  | 6537648 |  |  |  | 5880 | 66.29 | 63 |  | 253 | NC_008463.1/ CP000438.1 |
| PAK |  | 6395872 |  |  |  | 5769 | 66.44 | 63 |  | 693 | LR657304.1 |
| PALA52 | 71 | 6236756 | 2019 | Sputum | 1 | 5629 | 66.43 | 77 | 6236756 | 3016 | CP110347 |
| PALA8 | 74 | 6669237 | 2016 | Sputum | 1 | 6069 | 66.31 | 73 | 6669237 | 308 | CP104869 |
| PALA9 | 75 | 7126864 | 2018 | Sputum | 1 | 6471 | 65.91 | 75 | 7126864 | 179 | CP104870 |
| PALA10 | 80 | 6586512 | 2017 | Sputum | 2 | 5977 | 66.16 | 73 | 6532263 | 381 | JAPEVK000000000 |
| PALA45 | 113 | 6536263 | 2018 | Sputum | 1 | 5988 | 66.36 | 74 | 6536263 | 146 | CP110351 |
| PALA11 | 128 | 6330533 | 2019 | Sputum | 1 | 5751 | 66.48 | 75 | 6330533 | 954 | CP106680 |
| PALA12 | 360 | 6739580 | 2018 | Sputum | 1 | 6086 | 66.19 | 76 | 6739580 | 179 | CP106681 |
| PALA50 | 407 | 6675726 | 2019 | Sputum | 2 | 6076 | 65.99 | 75 | 6453705 | 241 | CP111034/CP111035 |
| PALA56 | 1633 | 6338470 | 2020 | Sputum | 1 | 5794 | 66.56 | 75 | 6338470 | 1239 | CP109919 |
| PALA20 | 1737 | 7003703 | 2018 | Sputum | 1 | 6447 | 66.01 | 74 | 7003703 | 235 | CP107064 |
| PALA21 | 2045 | 6988869 | 2019 | Sputum | 2 | 6440 | 66.03 | 75 | 6885451 | 17 | JAOVYS000000000 |
| PALA39 | 2145 | 7015423 | 2017 | Sputum | 1 | 6501 | 65.9 | 77 | 7015423 | 395 | CP109920 |
| PALA55 | 2417 | 6463386 | 2020 | Sputum | 1 | 5863 | 66.26 | 73 | 6463386 | 2552 | CP109918 |
| PALA22 | 112 | 6932838 | 2018 | Sputum | 1 | 6355 | 66.13 | 75 | 6932838 | 1123 | CP107275 |
| PALA23 | 100 | 6374444 | 2017 | Sputum | 1 | 5774 | 66.4 | 73 | 6374444 | 369 | CP109833 |
| PALA41 | 131 | 6437528 | 2017 | Sputum | 5 | 5867 | 66.41 | 75 | 6085978 | 2362 | JAPKIL000000000 |
| PALA42 | 312 | 6381417 | 2017 | Sputum | 1 | 5824 | 66.41 | 75 | 6381417 | 612 | CP109931 |
| PALA24 | 354 | 6780197 | 2017 | Sputum | 1 | 6251 | 66.11 | 77 | 6780197 | 348 | CP110344 |
| PALA57 | 2385 | 6840240 | 2020 | Sputum | 3 | 6333 | 65.98 | 78 | 4090943 | 348 | JAPKII000000000 |
| PALA48 | 2579 | 6540752 | 2018 | Sputum | 1 | 5984 | 66.36 | 74 | 6540752 | 146 | CP110353 |
| PALA25 | 73 | 6289320 | 2017 | Sputum | 1 | 5717 | 66.52 | 71 | 6289320 | 775 | CP109834 |
| PALA53 |  | 6292498 | 2019 | Sputum | 1 | 5713 | 66.52 | 69 | 6292498 | New | CP109856 |
| PALA43 | 366 | 6305107 | 2017 | Sputum | 1 | 5744 | 66.37 | 69 | 6305107 | New | CP109932 |
| PALA1 | 2641 | 6677289 | 2019 | Sputum | 1 | 6137 | 66.06 | 74 | 6677289 | 316 | CP104254 |
| PALA2 | 2605 | 6203666 | 2020 | Sputum | 1 | 5661 | 66.5 | 72 | 6203666 | 217 | CP104865 |
| PALA3 | 2474 | 6931652 | 2019 | Sputum | 2 | 6337 | 65.97 | 72 | 6930873 | 155 | JAPEVM000000000 |
| PALA4 | 2461 | 6832860 | 2019 | Sputum | 1 | 6194 | 66.02 | 71 | 6832860 | 671 | CP104866 |
| PALA5 | 2115 | 6962680 | 2019 | Cough Swab | 2 | 6460 | 66.05 | 77 | 6962157 | 175 | JAPEVL000000000 |
| PALA6 | 1740 | 6262103 | 2018 | Sputum | 1 | 5738 | 66.52 | 73 | 6262103 | 217 | CP104867 |
| PALA7 | 1462 | 6573599 | 2018 | Sputum | 1 | 5957 | 66.28 | 75 | 6573599 | 175 | CP104868 |
| PALA46 | 474 | 5844727 | 2018 | Sputum | 13 | 5354 | 66.25 | 73 | 3073432 | 485 | JAPKIJ000000000 |
| PALA13 | 2646 | 6879940 | 2019 | Sputum | 1 | 6293 | 66.1 | 75 | 6879940 | 17 | CP106682 |
| PALA14 | 2633 | 7015358 | 2020 | Sputum | 1 | 6512 | 65.91 | 78 | 7015358 | 395 | CP106742 |
| PALA49 | 2512 | 6624226 | 2019 | Sputum | 3 | 6081 | 66.29 | 68 | 4117471 | 27 | JAPKIK000000000 |
| PALA15 | 2482 | 6360180 | 2019 | Sputum | 1 | 5742 | 66.5 | 73 | 6360180 | 1033 | CP106743 |
| PALA16 | 2456 | 6910473 | 2019 | Sputum | 1 | 6349 | 66.03 | 75 | 6910473 | 155 | CP106744 |
| PALA47 | 1921 | 6576722 | 2018 | Sputum | 1 | 5957 | 66.28 | 75 | 6576722 | 175 | CP110352 |
| PALA17 |  | 6396161 | 2019 | Sputum | 1 | 5869 | 66.39 | 74 | 6396161 | 146 | CP106745 |
| PALA18 | 473 | 6966427 | 2019 | Sputum | 2 | 6275 | 65.8 | 75 | 6666127 | 261 | JAOZVO000000000 |
| PALA19 | 452 | 6504256 | 2020 | Sputum | 1 | 5948 | 66.3 | 75 | 6504256 | 882 | CP107029 |
| PALA36 | 2092 | 6844349 | 2015 | Sputum | 1 | 6268 | 66.03 | 72 | 6844349 | 532 | CP110348 |
| PALA32 | 2227 | 6575888 | 2015 | Sputum | 1 | 5973 | 66.28 | 73 | 6575888 | 485 | CP109844 |
| PALA31 | 2322 | 6425734 | 2015 | Sputum | 4 | 5820 | 66.22 | 73 | 4762364 | 260 | JAOXRP000000000 |
| PALA29 | 96 | 6748803 | 2014 | Cough Swab | 1 | 6264 | 66.13 | 76 | 6748803 | 27 | CP109843 |
| PALA27 | 2288 | 6806002 | 2014 | Cough Swab | 2 | 6257 | 66.2 | 77 | 6706518 | 27 | JAOZVP000000000 |
| PALA35 | 2101 | 6783004 | 2015 | Cough Swab | 1 | 6159 | 65.98 | 75 | 6783004 | New | CP110346 |
| PALA26 | 413 | 6733209 | 2014 | Sputum | 1 | 6156 | 66.15 | 72 | 6733209 | 253 | CP109835 |
| PALA40 |  | 7089219 | 2017 | Sputum | 1 | 6497 | 65.9 | 74 | 7089219 | 179 | CP110349 |
| PALA38 | 2263 | 6951781 | 2016 | Sputum | 2 | 6382 | 66.02 | 71 | 6774407 | 319 | CP111030/CP111031 |
| PALA54 |  | 6995283 | 2020 | Sputum | 2 | 6439 | 66.01 | 71 | 6817909 | 319 | CP111032/CP111033 |
| PALA30 |  | 6807710 | 2015 | Sputum | 1 | 6215 | 66.15 | 74 | 6807710 | 815 | CP110345 |
| PALA34 | 2462 | 6928454 | 2015 | Cough Swab | 1 | 6335 | 65.96 | 72 | 6928454 | 155 | CP109849 |
| PALA37 |  | 6437547 | 2016 | Cough Swab | 1 | 5807 | 66.25 | 71 | 6437547 | New | CP109850 |
| PALA28 | 111 | 6814760 | 2014 | Cough Swab | 2 | 6262 | 66.18 | 73 | 6771964 | 390 | JAOZVQ000000000 |
| PALA51 |  | 6420794 | 2019 | Sputum | 1 | 5898 | 66.37 | 75 | 6420794 | 386 | CP109851 |
| PALA33 | 2156 | 6948888 | 2015 | Cough Swab | 1 | 6395 | 66.06 | 76 | 6948888 | 17 | CP109845 |
| PALA44 |  | 6843186 | 2017 | Cough Swab | 1 | 6271 | 66.03 | 72 | 6843186 | 532 | CP110350 |
| Mean* |  | 6658993 |  |  | 2 | 6086 | 66.19 | 73 | 6462140 |  |  |

* 57 sequenced isolates (excludes reference genomes) *bp* = base pairs, CDS = coding DNA sequences

**Table S2:** Gene names, locus_tags, and references for downloaded *P. aeruginosa* strain PAO1 and PA14 T6SS components, effectors, immunity proteins, accessory proteins, and cognate secreted substrates.

| **Gene locus_tag** | | **Gene name** | **Alternative gene name** | **Reference(s)** |
| --- | --- | --- | --- | --- |
| **PA14 (RefSeq/Old)** | **PAO1** |  |  |  |
| PA14_RS00355/ PA14_00820 | PA0070 | *tagQ1* |  | Mougous *et al.,* 2006; Filloux *et al.,* 2008; Hsu *et al.,* 2009; Allsopp *et al.,* 2017 |
| PA14_RS00360/ PA14_00830 | PA0071 | *tagR1* |  | Filloux *et al.,* 2008; Mougous *et al.,* 2006; Hsu *et al.,* 2009; Allsopp *et al.,* 2017 |
| PA14_RS00365/ PA14_00850 | PA0072 | *tagS1* |  | Filloux *et al.,* 2008; Mougous *et al.,* 2006; Hsu *et al.,* 2009; Allsopp *et al.,* 2017 |
| PA14_RS00370/ PA14_00860 | PA0073 | *tagT1* |  | Filloux *et al.,* 2008; Mougous *et al.,* 2006; Hsu *et al.,* 2009; Allsopp *et al.,* 2017 |
| PA14_RS00375/ PA14_00875 | PA0074 | *ppkA* |  | Mougous *et al.,* 2007; Filloux *et al.,* 2008; Hsu *et al.,* 2009; Allsopp *et al.,* 2017 |
| PA14_RS00380/PA14_00890 | PA0075 | *pppA* |  | Filloux *et al.,* 2008; Mougous *et al.,* 2007; Hsu *et al.,* 2009; Allsopp *et al.,* 2017 |
| PA14_RS00385/PA14_00900 | PA0076 | *tagF1* |  | Filloux *et al.,* 2008; Mougous *et al.,* 2006; Hsu *et al.,* 2009; Allsopp *et al.,* 2017 |
| PA14_RS00390/ PA14_00910 | PA0077 | *tssM1* |  | Filloux *et al.,* 2008; Mougous *et al.,* 2006; Allsopp *et al.,* 2017 |
| PA14_RS00395/ PA14_00925 | PA0078 | *tssL1* |  | Filloux *et al.,* 2008; Mougous *et al.,* 2006; Allsopp *et al.,* 2017 |
| PA14_RS00400/ PA14_00940 | PA0079 | *tssK1* |  | Filloux *et al.,* 2008; Mougous *et al.,* 2006; Allsopp *et al.,* 2017 |
| PA14_RS00405/ PA14_00960 | PA0080 | *tssJ1* |  | Filloux *et al.,* 2008; Mougous *et al.,* 2006; Allsopp *et al.,* 2017 |
| PA14_RS31515/ PA14_00980 | PA0081 | *fha1* |  | Filloux *et al.,* 2008; Mougous *et al.,* 2006; Allsopp *et al.,* 2017 |
| PA14_RS00415/ PA14_00990 | PA0082 | *tssA1* |  | Filloux *et al.,* 2008; Mougous *et al.,* 2006; Allsopp *et al.,* 2017 |
| PA14_RS00420/ PA14_01010 | PA0083 | *tssB1* |  | Filloux *et al.,* 2008; Mougous *et al.,* 2006; Allsopp *et al.,* 2017 |
| PA14_RS00425/ PA14_01020 | PA0084 | *tssC1* |  | Filloux *et al.,* 2008; Mougous *et al.,* 2006; Allsopp *et al.,* 2017 |
| PA14_RS00430/ PA14_01030 | PA0085 | *hcp1* |  | Filloux *et al.,* 2008; Mougous *et al.,* 2006; Allsopp *et al.,* 2017 |
| PA14_RS00435/ PA14_01040 | PA0086 | *tagJ1* |  | Filloux *et al.,* 2008; Mougous *et al.,* 2006; Allsopp *et al.,* 2017 |
| PA14_RS00440/ PA14_01060 | PA0087 | *tssE1* |  | Filloux *et al.,* 2008; Mougous *et al.,* 2006; Allsopp *et al.,* 2017 |
| PA14_RS00445/ PA14_01070 | PA0088 | *tssF1* |  | Filloux *et al.,* 2008; Mougous *et al.,* 2006; Allsopp *et al.,* 2017 |
| PA14_RS00450/ PA14_01080 | PA0089 | *tssG1* |  | Filloux *et al.,* 2008; Mougous *et al.,* 2006; Allsopp *et al.,* 2017 |
| PA14_RS00455/ PA14_01100 | PA0090 | *clpV1* |  | Filloux *et al.,* 2008; Mougous *et al.,* 2006; Allsopp *et al.,* 2017 |
| PA14_RS00460/ PA14_01110 | PA0091 | *vgrG1a* |  | Hachani *et al.,* 2011 |
| PA14_RS00465/ PA14_01120 | PA0092 | *tsi6* | *tni1* | Whitney *et al.,* 2014; Alcoforado Diniz *et al.,* 2015 |
| PA14_RS00475/ PA14_01140 | PA0093 | *tse6* | *PAAR1/tas1/*  *tne1/tne3* | Hachani *et al.,* 2014; Whitney *et al.,* 2014; Alcoforado Diniz *et al.,* 2015; Ahmad *et al.,* 2019; This study |
| PA14_RS00480/ PA14_01150 | PA0094 | *eagT6* |  | Alcoforado Dinz *et al.,* 2015 |
| PA14_RS00485/ PA14_01160 | PA0095 | *vgrG1b* |  | Hachani *et al.,* 2011 |
| PA14_RS00490/ PA14_01170 | PA0096 |  |  | Filloux *et al.,* 2008; Mougous *et al.,* 2006; Allsopp *et al.,* 2017 |
| PA14_RS00495/ PA14_01180 | PA0097 |  |  | Filloux *et al.,* 2008; Mougous *et al.,* 2006; Allsopp *et al.,* 2017 |
| PA14_RS00500/ PA14_01190 | PA0098 |  |  | Filloux *et al.,* 2008; Mougous *et al.,* 2006; Allsopp *et al.,* 2017 |
| PA14_RS00505/ PA14_01200 | PA0099 | *tse7* | *tsd1* | Hachani *et al.,* 2014; Pissaridou *et al.,* 2018; This study |
| PA14_RS00510/ PA14_01220 | PA0100 | *tsi7* | *tsdI1* | Hachani *et al.,* 2014; Pissaridou *et al.,* 2018; This study |
| PA14_RS00515/ PA14_01230 | PA0101 |  |  | Filloux *et al.,* 2008; Mougous *et al.,* 2006; Allsopp *et al.,* 2017 |
| PA14_RS29980/ PA14_03190 | PA0259 | *tla3* |  | Berni *et al.,* 2019; Wood *et al.,* 2019a |
| PA14_RS01295/ PA14_03200 | PA0260 | *tle3* |  | Barret *et al.,* 2011; Russell *et al.,* 2013; Berni *et al.,* 2019; Wood *et al.,* 2019a |
| PA14_RS1300/ PA14_03210 | PA0261 | *tli3* |  | Barrett *et al.,* 2011; Russell *et al.,* 2013; Berni *et al.,* 2019; Wood *et al.,* 2019a |
| PA14_RS01305/ PA14_03220 | PA0262 | *vgrG2b* |  | Hachani *et al.,* 2011; Sana *et al.,* 2013; Wood *et al.,* 2019a |
| PA14_RS01310/ PA14_03240 | PA0263 | *hcpC* |  | Hachani *et al.,* 2011 |
| PA14_RS21915/PA14_53820 | PA0807 | *ampDh3* |  | Wang *et al.,* 2020 |
| PA14_RS21910/PA14_53810 | PA0808 |  |  | Wang *et al.,* 2020 |
| PA14_RS21845/PA14_53660 | PA0821 | *tsiV* | *tpiA* | Wang *et al.,* 2021a |
| PA14_RS21840 | PA0822 | *tseV* | *tpnA* | Wang *et al.,* 2021a |
| PA14_RS21835/ PA14_53640 | PA0824 | *PAAR2* |  | Wood *et al.,* 2019b |
| PA14_RS18265/ PA14_44930 | PA1508 | *PAAR3* |  | Wood *et al.,* 2019b |
| PA14_RS18260/ PA14_44920 | PA1509 | *tli4* | *tli4a* | Jiang *et al.,* 2016 |
| PA14_RS18255/ PA14_44910 | PA1510 | *tle4* | *tplE/tle4a* | Jiang *et al.,* 2016; Russell *et al.,* 2013 |
| PA14_RS18250/ PA14_44900 | PA1511 | *vgrG2a* |  | Hachani *et al.,* 2011 |
| PA14_RS18245/ PA14_44890 | PA1512 | *hcpA* | *hcp2* | Filloux *et al.,* 2008; Mougous *et al.,* 2006; Allsopp *et al.,* 2017 |
| PA14_RS17490/ PA14_43050 | PA1656 | *tssA2* |  | Filloux *et al.,* 2008; Mougous *et al.,* 2006; Allsopp *et al.,* 2017 |
| PA14_RS17485/ PA14_43040 | PA1657 | *tssB2* |  | Filloux *et al.,* 2008; Mougous *et al.,* 2006; Allsopp *et al.,* 2017 |
| PA14_RS17480/ PA14_43030 | PA1658 | *tssC2* |  | Filloux *et al.,* 2008; Mougous *et al.,* 2006; Allsopp *et al.,* 2017 |
| PA14_RS17475/ PA14_43020 | PA1659 | *tssE2* |  | Filloux *et al.,* 2008; Mougous *et al.,* 2006; Allsopp *et al.,* 2017 |
| PA14_RS31155 | PA1659.1 | *PAAR5* |  | Wood *et al.,* 2019b |
| PA14_RS17470/ PA14_43000 | PA1660 | *tssF2* |  | Filloux *et al.,* 2008; Mougous *et al.,* 2006; Allsopp *et al.,* 2017 |
| PA14_RS17465/ PA14_42990 | PA1661 | *tssG2* |  | Filloux *et al.,* 2008; Mougous *et al.,* 2006; Allsopp *et al.,* 2017 |
| PA14_RS17460/ PA14_42980 | PA1662 | *clpV2* |  | Filloux *et al.,* 2008; Mougous *et al.,* 2006; Allsopp *et al.,* 2017 |
| PA14_RS17455/ PA14_42970 | PA1663 | *sfa2* |  | Filloux *et al.,* 2008; Mougous *et al.,* 2006; Allsopp *et al.,* 2017; Allsopp *et al.,* 2022 |
| PA14_RS17450/ PA14_42960 | PA1664 | *orfX* |  | Filloux *et al.,* 2008; Mougous *et al.,* 2006; Allsopp *et al.,* 2017 |
| PA14_RS17445/ PA14_42950 | PA1665 | *fha2* |  | Filloux *et al.,* 2008; Mougous *et al.,* 2006; Allsopp *et al.,* 2017 |
| PA14_RS17440/ PA14_42940 | PA1666 | *tssJ2* |  | Filloux *et al.,* 2008; Mougous *et al.,* 2006; Allsopp *et al.,* 2017 |
| PA14_RS17435/ PA14_42920 | PA1667 | *tssK2* |  | Filloux *et al.,* 2008; Mougous *et al.,* 2006; Allsopp *et al.,* 2017 |
| PA14_RS17430/ PA14_42910 | PA1668 | *tssL2* |  | Filloux *et al.,* 2008; Mougous *et al.,* 2006; Allsopp *et al.,* 2017 |
| PA14_RS17425/ PA14_42900 | PA1669 | *tssM2* |  | Filloux *et al.,* 2008; Mougous *et al.,* 2006; Allsopp *et al.,* 2017 |
| PA14_RS17420/ PA14_42890 | PA1670 | *stp1* |  | Filloux *et al.,* 2008; Mougous *et al.,* 2006; Allsopp *et al.,* 2017 |
| PA14_RS17415/ PA14_42880 | PA1671 | *stk1* |  | Filloux *et al.,* 2008; Mougous *et al.,* 2006; Allsopp *et al.,* 2017 |
| PA14_RS16485/ PA14_40660 | PA1844 | *tse1* | *tae1* | Hood *et al.,* 2010; Russell *et al.,* 2011; Silverman *et al.,* 2013 |
| PA14_RS16480/ PA14_40650 | PA1845 | *tsi1* |  | Hood *et al.,* 2010; Silverman *et al.,* 2013; Russell *et al.,* 2011 |
| PA14_RS16385/ PA14_40390 | PA1863 | *modA* |  | Wang *et al.,* 2021b |
| PA14_RS13925/ PA14_34150 | PA2359 | *sfa3* |  | Filloux *et al.,* 2008; Mougous *et al.,* 2006; Allsopp *et al.,* 2017 |
| PA14_RS13920/ PA14_34140 | PA2360 | *tssA3* |  | Filloux *et al.,* 2008; Mougous *et al.,* 2006; Allsopp *et al.,* 2017 |
| PA14_RS13915/ PA14_34130 | PA2361 | *tssM3* |  | Filloux *et al.,* 2008; Mougous *et al.,* 2006; Allsopp *et al.,* 2017 |
| PA14_RS13910/ PA14_34110 | PA2362 | *tssL3* |  | Filloux *et al.,* 2008; Mougous *et al.,* 2006; Allsopp *et al.,* 2017 |
| PA14_RS13905/ PA14_34100 | PA2363 | *tssK3* |  | Filloux *et al.,* 2008; Mougous *et al.,* 2006; Allsopp *et al.,* 2017 |
| PA14_RS13900/ PA14_34080 | PA2364 | *tssJ3* |  | Filloux *et al.,* 2008; Mougous *et al.,* 2006; Allsopp *et al.,* 2017 |
| PA14_RS13895/ PA14_34070 | PA2365 | *tssB3* |  | Filloux *et al.,* 2008; Mougous *et al.,* 2006; Allsopp *et al.,* 2017 |
| PA14_RS13890/ PA14_34050 | PA2366 | *tssC3* |  | Filloux *et al.,* 2008; Mougous *et al.,* 2006; Allsopp *et al.,* 2017 |
| PA14_RS13885/ PA14_34030 | PA2367 | *hcp3* |  | Filloux *et al.,* 2008; Mougous *et al.,* 2006; Allsopp *et al.,* 2017 |
| PA14_RS13880/ PA14_34020 | PA2368 | *tssE3* |  | Filloux *et al.,* 2008; Mougous *et al.,* 2006; Allsopp *et al.,* 2017 |
| PA14_RS13875/ PA14_34010 | PA2369 | *tssF3* |  | Filloux *et al.,* 2008; Mougous *et al.,* 2006; Allsopp *et al.,* 2017 |
| PA14_RS13870/ PA14_34000 | PA2370 | *tssG3* |  | Filloux *et al.,* 2008; Mougous *et al.,* 2006; Allsopp *et al.,* 2017 |
| PA14_RS13865/ PA14_33990 | PA2371 | *clpV3* |  | Filloux *et al.,* 2008; Mougous *et al.,* 2006; Allsopp *et al.,* 2017 |
|  | PA2372 |  |  | Filloux *et al.,* 2008; Mougous *et al.,* 2006; Allsopp *et al.,* 2017 |
| PA14_RS13850/ PA14_33960 | PA2373 | *vgrG3* |  | Hachani *et al.,* 2011 |
| PA14_RS13845/ PA14_33940 | PA2374 | *tseF* |  | Lin *et al.,* 2017 |
| PA14_RS13840/ PA14_33930 | PA2375 | *PAAR-like* |  | Wood *et al.,* 2019b |
| PA14_RS11990/ PA14_29400 | PA2684 | *tse5* | *rhsP1* | Hachani *et al.,* 2014; Whitney *et al.,* 2014 |
| PA14_RS11995 | PA2684.1 | *tsi5* |  | Hachani *et al.,* 2014; Whitney *et al.,* 2014 |
| PA14_RS11985/ PA14_29390 | PA2685 | *vgrG1c* | *vgrG4* | Hood *et al.,* 2010 |
| PA14_RS11900/ PA14_29200 | PA2702 | *tse2* |  | Hood *et al.,* 2010; Silverman *et al.,* 2013; Russell *et al.,* 2011; Robb *et al.,* 2016 |
| PA14_RS11895/ PA14_29190 | PA2703 | *tsi2* |  | Hood *et al.,* 2010; Silverman *et al.,* 2013; Russell *et al.,* 2011 |
| PA14_RS11455/ PA14_28210 | PA2774 | *tse4* |  | Whitney *et al.,* 2014; LaCourse *et al.,* 2018 |
| PA14_RS11450/ PA14_28200 | PA2775 | *tsi4* |  | Whitney *et al.,* 2014 |
| PA14_RS30250/ PA14_21490 | PA3290 | *tle1* |  | Barrett *et al.,* 2011; Russell *et al.,* 2013 |
| PA14_RS08635/ PA14_21480 | PA3291 | *tli1a* |  | Barrett *et al.,* 2011; Russell *et al.,* 2013 |
| PA14_RS31380/ PA14_21470 | PA3292 | *tli1b* |  | Barrett *et al.,* 2011; Russell *et al.,* 2013 |
| PA14_RS08630/ PA14_21460 | PA3293 | *tap4a* |  | Wood *et al.,* 2019a |
| PA14_RS08625/ PA14_21450 | PA3294 | *vgrG4a* |  | Hachani *et al.,* 2011 |
| PA14_RS07625/ PA14_19020 | PA3484 | *tse3* |  | Hood *et al.,* 2010; Silverman *et al.,* 2013; Russell *et al.,* 2011 |
| PA14_RS07620/ PA14_19010 | PA3485 | *tsi3* |  | Hood *et al.,* 2010; Silverman *et al.,* 2013; Russell *et al.,* 2011 |
| PA14_RS07615/ PA14_18985 | PA3486 | *vgrG4b* |  | Hachani *et al.,* 2011 |
| PA14_RS07610/ PA14_18970 | PA3487 | *pldA* | *tle5a* | Russell *et al.,* 2013; Wilderman *et al.,* 2001 |
| PA14_RS07605/ PA14_18960 | PA3488 | *tli5a* |  | Russell *et al.,* 2013 |
| PA14_RS05390/ PA14_13390 | PA3904 | *PAAR4* |  | Burkinshaw *et al.,* 2018 |
| PA14_RS05385/ PA14_13380 | PA3905 | *tecT* | *tapN* | Burkinshaw *et al.,* 2018 |
| PA14_RS05380/ PA14_13370 | PA3906 | *coTecT* |  | Burkinshaw *et al.,* 2018 |
| PA14_RS30125/ PA14_13360 | PA3907 | *tseT* |  | Burkinshaw *et al.,* 2018 |
| PA14_RS05370/ PA14_13350 | PA3908 | *tsiT* |  | Burkinshaw *et al.,* 2018 |
| PA14_RS04045/ PA14_10010 | PA4163 | *tse8* |  | Nolan *et al.,* 2021 |
| PA14_RS04050/ PA14_10020 | PA4164 | *tsi8* |  | Nolan *et al.,* 2021 |
| PA14_RS26580/ PA14_65000 | PA4922 | *azu* |  | Han *et al.,* 2019 |
| PA14_RS27415/ PA14_67190 | PA5086 | *tli5b_3_* |  | Jiang *et al.,* 2014 |
| PA14_RS27420/ PA14_67200 | PA5087 | *tli5b_2_* |  | Jiang *et al.,* 2014 |
| PA14_RS31055/ PA14_67210 | PA5088 | *tli5b_1_* |  | Jiang *et al.,* 2014 |
| PA14_RS27425/ PA14_67220 | PA5089 | *pldB* | *tle5b* | Jiang *et al.,* 2014; Russell *et al.,* 2013; Wettstadt *et al.,* 2019 |
| PA14_RS27430/ PA14_67230 | PA5090 | *vgrG5* |  | Hachani *et al.,* 2011 |
| PA14_RS28330/ PA14_69510 | PA5264 | *tspI1a/b/c* |  | This study |
| PA14_RS28335/ PA14_69520 | PA5265 | *tspE1a/b/c* |  | This study |
| PA14_RS28340/ PA14_69540 |  | *DUF4123* |  | This study |
| PA14_RS28345/ PA14_69550 | PA5266 | *vgrG6* |  | Hachani *et al.,* 2011 |
| PA14_RS28350/ PA14_69560 | PA5267 | *hcpB* |  | Hachani *et al.,* 2011 |
| PA14_RS00470/ PA14_01130 |  | *tis1* |  | Ahmad *et al.,* 2019 |
| PA14_RS17495/ PA14_43070 |  | *hcp2* |  | Hachani *et al.,* 2014 |
| PA14_RS17500/ PA14_43080 |  | *vgrG14* |  | Hachani *et al.,* 2014; Jones *et al.,* 2014 |
| PA14_RS17505/ PA14_43090 |  | *tap14* |  | Hachani *et al.,* 2014 |
| PA14_RS17510/ PA14_43100 |  | *rhsP2* |  | Hachani *et al.,* 2014; Jones *et al.,* 2014 |
| PA14_RS30650 |  | *rhsI2* |  | Hachani *et al.,* 2014 |
| PA14_RS31050/ PA14_67180 |  | *tli5b_4_* |  | Allsopp *et al.,* 2017 |
| PA14_RS13860/ PA14_33980 |  | *tsd2* |  | This study; Allsopp *et al.,* 2017 |
| PA14_RS13855/ PA14_33970 |  | *tpe2* |  | This study; Allsopp *et al.,* 2017 |
| N/A | N/A | *vgrG7** |  | This study |

Note * = *vgrG7* is not present in PA14 or PAO1. Is present in PAK (LR657304.1) and PA7 (CP000744.1).

**Table S4:** Significantly enriched COGs in both the core and accessory genomes of the investigated *P. aeruginosa* pangenome. Statistical analysis was conducted using a Chi-squared test and a Bonferroni corrected *p-*value < 0.0025. Log odds ratio (LOR) relative to the core genome is also shown.

| **COG Category** | **Proportion of genes in core (%)** | **Proportion of genes in accessory (%)** | ***p-*value^*^** | **LOR (three s.f.)** |
| --- | --- | --- | --- | --- |
| **Core genome** | | | | |
| J: Translation/ribosomal structure | 3.911 | 0.442 | < 0.001 | 0.962 |
| E: Amino acid transport/metabolism | 9.415 | 3.414 | < 0.001 | 0.468 |
| C: Energy production/conversion | 6.192 | 2.348 | < 0.001 | 0.439 |
| F: Nucleotide transport/metabolism | 2.299 | 0.431 | < 0.001 | 0.736 |
| T: Signal transduction | 5.667 | 2.585 | < 0.001 | 0.355 |
| K: Transcription | 8.981 | 7.529 | 0.0017 | 0.083 |
| G: Carbohydrate transport/metabolism | 4.219 | 1.573 | < 0.001 | 0.440 |
| P: Inorganic ion metabolism | 6.355 | 3.414 | < 0.001 | 0.283 |
| I: Lipid transport/metabolism | 4.436 | 2.143 | < 0.001 | 0.326 |
| H: Coenzyme transport/metabolism | 3.476 | 1.303 | < 0.001 | 0.436 |
| O: Chaperones | 3.205 | 2.090 | < 0.001 | 0.191 |
| M: Cell wall/membrane/envelope biogenesis | 4.871 | 3.296 | < 0.001 | 0.177 |
| Q: Secondary metabolite biosynthesis/transport/catabolism | 3.150 | 1.874 | < 0.001 | 0.231 |
| **Accessory genome** | | | | |
| L: Replication/recombination/repair | 2.299 | 16.34 | < 0.001 | -0.919 |
| V: Defence mechanisms | 1.032 | 2.273 | < 0.001 | -0.348 |
| U: Intracellular trafficking/secretion | 3.150 | 4.136 | 0.0023 | -0.122 |
| S: Function unknown | 19.28 | 22.99 | < 0.001 | -0.096 |

^*^Chi-squared (χ^2^) test s.f. = significant figures

**Table S6:** Presence of characterised and putative T6SS effectors within the 52 clinical *P. aeruginosa* isolates under study (NP = Not present).

|  |  | PA0093 | PA0099 | PA0260 | PA0262 | PA0807 | PA0822 | PA1510 | PA1844 | PA1863 | PA2372 | PA2374 | PA2684 | PA2702 | PA2774 | PA3290 | PA3484 | PA3487 | PA3907 | PA4163 | PA4922 | PA5089 | PA5265 | PA14_RS17510 | PAKAF_03765 |
| --- | --- | --- | --- | --- | --- | --- | --- | --- | --- | --- | --- | --- | --- | --- | --- | --- | --- | --- | --- | --- | --- | --- | --- | --- | --- |
| **Isolate ID** | **Patient ID** | *tse6* | *tse7* | *tle3* | *vgrG2b* | *ampDh3* | *tseV* | *tle4* | *tse1* | *modA* |  | *tseF* | *tse5* | *tse2* | *tse4* | *tle1* | *tse3* | *pldA* | *tseT* | *tse8* | *azu* | *pldB* | *tspE1* | *rhsP2* | *tle2* |
| PALA26 | 413 | *tas1* |  |  |  |  |  |  |  |  | *tsd2 + tpe2* |  |  |  |  |  |  |  |  |  |  |  | *tspE1c* |  | NP |
| PALA27 | 2288 |  |  |  |  |  |  |  |  |  |  |  |  |  |  |  |  | NP |  |  |  |  | *tspE1c* | NP | NP |
| PALA28 | 111 |  |  |  |  |  |  |  |  |  | *tsd2 + tpe2* |  |  |  |  |  |  | NP |  |  |  |  | *tspE1c* | NP | NP |
| PALA29 | 96 |  |  |  |  |  |  |  |  |  |  |  |  |  |  |  |  | NP |  |  |  |  | *tspE1c* | NP | NP |
| PALA30 | 2263 | *tas1* |  |  |  |  |  |  |  |  | *tsd2 + tpe2* |  |  |  |  |  |  |  |  |  |  |  | *tspE1c* |  | NP |
| PALA32 | 2227 |  |  |  |  |  | NP |  |  |  |  |  |  |  |  |  |  | NP |  |  |  | NP | *tspE1b* | NP | NP |
| PALA33 | 2156 |  |  | NP | NP |  |  |  |  |  |  |  |  |  |  |  |  | NP |  |  |  |  | *tspE1b* | NP | NP |
| PALA34 | 2462 |  |  |  |  |  | NP |  |  |  |  |  |  |  |  |  |  | NP |  |  |  |  | *tspE1b* | NP | NP |
| PALA35 | 2101 | *tne3* | *tsd1* |  |  |  |  |  |  |  | *tsd2 + tpe2* |  |  |  |  |  |  | NP |  |  |  |  | *tspE1b* |  |  |
| PALA36 | 2092 |  |  |  |  |  | x2 |  |  |  |  |  |  |  |  |  |  |  |  |  |  |  | *tspE1c* |  |  |
| PALA37 | 2462 | *tne3* | *tsd1* | NP | NP |  |  |  |  |  | *tsd2 + tpe2* |  |  |  | NP |  |  | NP |  |  |  |  | *tspE1c* |  |  |
| PALA38 | 2263 | *tas1* |  |  |  |  |  |  |  |  | *tsd2 + tpe2* |  |  |  |  |  |  | NP |  |  |  |  | *tspE1c* |  | NP |
| PALA8 | 74 |  |  |  |  |  | NP |  |  |  | *tsd2 + tpe2* | x3 |  |  |  |  |  |  |  |  |  |  | *tspE1c* |  | NP |
| PALA39 | 2145 |  |  |  |  |  |  |  |  |  |  |  |  |  |  |  |  | NP |  |  |  |  | *tspE1b* | NP | NP |
| PALA40 | 413 |  |  |  |  |  | NP |  |  |  |  |  |  |  |  |  |  | NP |  |  |  |  | *tspE1b* | NP | NP |
| PALA10 | 80 |  |  |  |  |  |  |  |  |  |  |  |  |  |  |  |  | NP |  |  |  |  | *tspE1b* | NP | NP |
| PALA24 | 354 |  |  |  |  |  |  |  |  |  | *tsd2 + tpe2* |  |  |  |  |  |  | NP |  |  |  |  | *tspE1b* |  | NP |
| PALA42 | 312 |  |  |  |  |  |  |  |  |  |  |  |  |  |  |  |  | NP |  |  |  |  | *tspE1c* | NP | NP |
| PALA23 | 100 | *tas1* |  | NP | NP |  |  |  |  |  | *tsd2 + tpe2* |  |  |  |  |  |  | NP |  |  |  |  | *tspE1b* | NP | NP |
| PALA25 | 73 | *tas1* |  |  |  |  | NP |  |  |  |  |  |  |  |  |  |  | NP |  |  |  |  | *tspE1b* | NP | NP |
| PALA43 | 366 |  |  |  |  |  |  |  |  |  |  |  |  |  |  |  |  | NP |  |  |  |  | *tspE1b* | NP | NP |
| PALA44 | 2156 |  |  |  |  |  | x2 |  |  |  |  |  |  |  |  |  |  |  |  |  |  |  | *tspE1c* |  |  |
| PALA9 | 75 |  |  |  |  |  | NP |  |  |  |  |  |  |  |  |  |  | NP |  |  |  |  | *tspE1b* | NP | NP |
| PALA45 | 113 |  |  |  |  |  |  |  |  |  | *tsd2 + tpe2* |  |  |  |  |  |  | NP |  |  |  |  | *tspE1b* | NP | NP |
| PALA22 | 112 |  |  |  |  |  |  |  |  |  |  |  |  |  |  |  |  | NP |  |  |  |  | *tspE1b* | NP | NP |
| PALA20 | 1737 | *tas1* |  |  |  |  | NP |  |  |  | *tsd2 + tpe2* |  |  |  |  |  |  |  |  |  |  |  | *tspE1b* |  | NP |
| PALA12 | 360 |  |  |  |  |  | NP |  |  |  |  |  |  |  |  |  |  | NP |  |  |  |  | *tspE1b* | NP | NP |
| PALA6 | 1740 |  |  |  |  |  | NP |  |  |  |  |  |  |  |  |  |  | NP |  |  |  |  | *tspE1b* | NP | NP |
| PALA7 | 1462 |  |  |  |  |  | NP |  |  |  |  |  |  |  |  |  |  | NP |  |  |  | NP | *tspE1b* | NP | NP |
| PALA47 | 1921 |  |  |  |  |  | NP |  |  |  |  |  |  |  |  |  |  | NP |  |  |  | NP | *tspE1b* | NP | NP |
| PALA48 | 2579 |  |  |  |  |  |  |  |  |  | *tsd2 + tpe2* |  |  |  |  |  |  | NP |  |  |  |  | *tspE1b* | NP | NP |
| PALA16 | 2456 |  |  |  |  |  | NP |  |  |  |  |  |  |  |  |  |  | NP |  |  |  |  | *tspE1b* | NP | NP |
| PALA15 | 2482 |  |  |  |  |  | NP |  |  |  |  |  |  |  |  |  |  | NP |  |  |  | NP | *tspE1b* | NP | NP |
| PALA4 | 2461 | *tas1* |  |  |  |  |  |  |  |  | *tsd2 + tpe2* |  |  |  |  |  |  |  |  |  |  |  | *tspE1b* |  | NP |
| PALA18 | 473 |  |  |  |  |  |  |  |  |  | *tsd2 + tpe2* |  |  |  |  |  |  | NP |  |  |  |  | *tspE1b* | NP | NP |
| PALA50 | 407 |  |  |  |  |  | NP |  |  |  |  |  |  |  |  |  |  | NP |  |  |  |  | *tspE1b* | NP | NP |
| PALA3 | 2474 |  |  |  |  |  | NP |  |  |  |  |  |  |  |  |  |  | NP |  |  |  |  | *tspE1b* | NP | NP |
| PALA1 | 2641 | *tas1* |  | NP | NP |  |  |  |  |  |  |  |  |  |  |  |  |  |  |  |  |  | *tspE1b* | NP |  |
| PALA13 | 2646 |  |  | NP | NP |  |  |  |  |  |  |  |  |  |  |  |  | NP |  |  |  |  | *tspE1b* | NP | NP |
| PALA17 | 1921 |  |  |  |  |  |  |  |  |  | *tsd2 + tpe2* |  |  |  |  |  |  | NP |  | NP |  |  | *tspE1b* | NP | NP |
| PALA11 | 128 |  |  |  |  |  | NP |  |  |  |  |  |  |  |  |  |  | NP |  |  |  |  | *tspE1c* | NP | NP |
| PALA5 | 2115 |  |  |  |  |  | NP |  |  |  |  |  |  |  |  |  |  | NP |  |  |  | NP | *tspE1b* | NP | NP |
| PALA51 | 111 |  |  |  |  |  | NP |  |  |  |  |  |  |  |  |  |  | NP |  |  |  |  | *tspE1b* | NP | NP |
| PALA52 | 71 |  |  |  |  |  | NP |  |  |  |  |  |  |  |  |  |  | NP |  |  |  |  | *tspE1a* | NP | NP |
| PALA21 | 2045 |  |  | NP | NP |  |  |  |  |  |  |  |  |  |  |  |  | NP |  |  |  |  | *tspE1b* | NP | NP |
| PALA53 | 73 | *tas1* |  |  |  |  | NP |  |  |  |  |  |  |  |  |  |  | NP |  |  |  |  | *tspE1b* | NP | NP |
| PALA54 | 2263 | *tas1* |  |  |  |  |  |  |  |  | *tsd2 + tpe2* |  |  |  |  |  |  | NP |  |  |  |  | *tspE1c* |  | NP |
| PALA55 | 2417 |  |  |  |  |  |  |  |  |  |  |  |  |  |  |  |  | NP |  |  |  |  | *tspE1b* | NP | NP |
| PALA14 | 2633 |  |  |  |  |  |  |  |  |  |  |  |  |  |  |  |  | NP |  |  |  |  | *tspE1b* | NP | NP |
| PALA56 | 1633 |  |  |  |  |  |  |  |  |  |  |  |  |  |  |  |  | NP |  |  |  |  | *tspE1b* | NP | NP |
| PALA19 | 452 |  |  |  |  |  |  |  |  |  |  |  |  |  |  |  |  | NP |  |  |  |  | *tspE1b* | NP | NP |
| PALA2 | 2605 |  |  |  |  |  | NP |  |  |  |  |  |  |  |  |  |  | NP |  |  |  |  | *tspE1b* | NP | NP |

**Supplementary References**

Ahmad, S., Wang, B., Walker, M. D., Tran, H. K. R., Stogios, P. J., Savchenko, A., et al. (2019). An interbacterial toxin inhibits target cell growth by synthesizing (p)ppApp. *Nature 2019 575:7784* 575, 674–678. doi: 10.1038/s41586-019-1735-9.

Alcoforado Diniz, J., Liu, Y. C., and Coulthurst, S. J. (2015). Molecular weaponry: Diverse effectors delivered by the Type VI secretion system. *Cellular Microbiology* 17, 1742–1751. doi: 10.1111/CMI.12532.

Allsopp, L. P., Collins, A. C. Z., Hawkins, E., Wood, T. E., and Filloux, A. (2022). RpoN/Sfa2-dependent activation of the *Pseudomonas aeruginosa* H2-T6SS and its cognate arsenal of antibacterial toxins. *Nucleic Acids Research* 50, 227–243. doi: 10.1093/NAR/GKAB1254.

Allsopp, L. P., Wood, T. E., Howard, S. A., Maggiorelli, F., Nolan, L. M., Wettstadt, S., et al. (2017). RsmA and AmrZ orchestrate the assembly of all three type VI secretion systems in *Pseudomonas aeruginosa*. *Proc Natl Acad Sci U S A* 114, 7707–7712. doi: 10.1073/pnas.1700286114.

Barret, M., Egan, F., Fargier, E., Morrissey, J. P., and O’Gara, F. (2011). Genomic analysis of the type VI secretion systems in Pseudomonas spp.: novel clusters and putative effectors uncovered. *Microbiology (N Y)* 157, 1726–1739. doi: 10.1099/MIC.0.048645-0.

Berni, B., Soscia, C., Djermoun, S., Ize, B., and Bleves, S. (2019). A Type VI Secretion System Trans-Kingdom Effector Is Required for the Delivery of a Novel Antibacterial Toxin in *Pseudomonas aeruginosa*. *Frontiers in Microbiology* 10, 1218. doi: 10.3389/fmicb.2019.01218.

Burkinshaw, B. J., Liang, X., Wong, M., Le, A. N. H., Lam, L., and Dong, T. G. (2018). A type VI secretion system effector delivery mechanism dependent on PAAR and a chaperone–co-chaperone complex. *Nature Microbiology 2018 3:5* 3, 632–640. doi: 10.1038/s41564-018-0144-4.

Filloux, A., Hachani, A., and Bleves, S. (2008). The bacterial type VI secretion machine: yet another player for protein transport across membranes. *Microbiology (N Y)* 154, 1570–1583. doi: 10.1099/MIC.0.2008/016840-0.

Hachani, A., Allsopp, L. P., Oduko, Y., and Filloux, A. (2014). The VgrG proteins are “à la carte” delivery systems for bacterial type VI effectors. *Journal of Biological Chemistry* 289, 17872–17884. doi: 10.1074/jbc.M114.563429.

Hachani, A., Lossi, N. S., Hamilton, A., Jones, C., Bleves, S., Albesa-Jové, D., et al. (2011). Type VI Secretion System in *Pseudomonas aeruginosa*: Secretion and Multimerization of VgrG Proteins. *Journal of Biological Chemistry* 286, 12317–12327. doi: 10.1074/JBC.M110.193045.

Han, Y., Wang, T., Chen, G., Pu, Q., Liu, Q., Zhang, Y., et al. (2019). A *Pseudomonas aeruginosa* type VI secretion system regulated by CueR facilitates copper acquisition. *PLOS Pathogens* 15, e1008198. doi: 10.1371/journal.ppat.1008198.

Hood, R. D., Singh, P., Hsu, F. S., Güvener, T., Carl, M. A., Trinidad, R. R. S., et al. (2010). A Type VI Secretion System of *Pseudomonas aeruginosa* Targets a Toxin to Bacteria. *Cell Host and Microbe* 7, 25–37. doi: 10.1016/j.chom.2009.12.007.

Hsu, F., Schwarz, S., and Mougous, J. D. (2009). TagR promotes PpkA-catalysed type VI secretion activation in *Pseudomonas aeruginosa*. *Molecular Microbiology* 72, 1111–1125. doi: 10.1111/J.1365-2958.2009.06701.X.

Jiang, F., Wang, X., Wang, B., Chen, L., Zhao, Z., Waterfield, N. R., et al. (2016). The *Pseudomonas aeruginosa* Type VI Secretion PGAP1-like Effector Induces Host Autophagy by Activating Endoplasmic Reticulum Stress. *Cell Reports* 16, 1502–1509. doi: 10.1016/j.celrep.2016.07.012.

Jiang, F., Waterfield, N. R., Yang, J., Yang, G., and Jin, Q. (2014). A *Pseudomonas aeruginosa* type VI secretion phospholipase D effector targets both prokaryotic and eukaryotic cells. *Cell Host and Microbe* 15, 600–610. doi: 10.1016/j.chom.2014.04.010.

Jones, C., Hachani, A., Manoli, E., and Filloux, A. (2014). An rhs gene linked to the second type VI secretion cluster is a feature of the *Pseudomonas aeruginosa* strain PA14. *Journal of Bacteriology* 196, 800–810. doi: 10.1128/JB.00863-13.

LaCourse, K. D., Peterson, S. B., Kulasekara, H. D., Radey, M. C., Kim, J., and Mougous, J. D. (2018). Conditional toxicity and synergy drive diversity among antibacterial effectors. *Nature Microbiology 2018 3:4* 3, 440–446. doi: 10.1038/s41564-018-0113-y.

Lin, J., Zhang, W., Cheng, J., Yang, X., Zhu, K., Wang, Y., et al. (2017). A Pseudomonas T6SS effector recruits PQS-containing outer membrane vesicles for iron acquisition. *Nature Communications* 8, 1–12. doi: 10.1038/ncomms14888.

Mougous, J. D., Cuff, M. E., Raunser, S., Shen, A., Zhou, M., Gifford, C. A., et al. (2006). A virulence locus of *Pseudomonas aeruginosa* encodes a protein secretion apparatus. *Science (1979)* 312, 1526–1530. doi: 10.1126/SCIENCE.1128393.

Mougous, J. D., Gifford, C. A., Ramsdell, T. L., and Mekalanos, J. J. (2007). Threonine phosphorylation post-translationally regulates protein secretion in *Pseudomonas aeruginosa*. *Nature Cell Biology 2007 9:7* 9, 797–803. doi: 10.1038/ncb1605.

Nolan, L. M., Cain, A. K., Clamens, T., Furniss, R. C. D., Manoli, E., Sainz-Polo, M. A., et al. (2021). Identification of Tse8 as a Type VI secretion system toxin from *Pseudomonas aeruginosa* that targets the bacterial transamidosome to inhibit protein synthesis in prey cells. *Nature Microbiology 2021 6:9* 6, 1199–1210. doi: 10.1038/s41564-021-00950-8.

Pissaridou, P., Allsopp, L. P., Wettstadt, S., Howard, S. A., Mavridou, D. A. I., and Filloux, A. (2018). The *Pseudomonas aeruginosa* T6SS-VgrG1b spike is topped by a PAAR protein eliciting DNA damage to bacterial competitors. *Proceedings of the National Acad Sciences USA* 115, 12519–12524. doi: 10.1073/pnas.1814181115.

Robb, C. S., Robb, M., Nano, F. E., and Boraston, A. B. (2016). The Structure of the Toxin and Type Six Secretion System Substrate Tse2 in Complex with Its Immunity Protein. *Structure* 24, 277–284. doi: 10.1016/J.STR.2015.11.012.

Russell, A. B., Hood, R. D., Bui, N. K., Leroux, M., Vollmer, W., and Mougous, J. D. (2011). Type VI secretion delivers bacteriolytic effectors to target cells. *Nature* 475, 343–349. doi: 10.1038/nature10244.

Russell, A. B., Leroux, M., Hathazi, K., Agnello, D. M., Ishikawa, T., Wiggins, P. A., et al. (2013). Diverse type VI secretion phospholipases are functionally plastic antibacterial effectors. *Nature* 496, 508–512. doi: 10.1038/nature12074.

Sana, T. G., Soscia, C., Tonglet, C. M., Garvis, S., and Bleves, S. (2013). Divergent Control of Two Type VI Secretion Systems by RpoN in *Pseudomonas aeruginosa*. *PLOS ONE* 8, e76030. doi: 10.1371/JOURNAL.PONE.0076030.

Silverman, J. M., Agnello, D. M., Zheng, H., Andrews, B. T., Li, M., Catalano, C. E., et al. (2013). Haemolysin Coregulated Protein Is an Exported Receptor and Chaperone of Type VI Secretion Substrates. *Molecular Cell* 51, 584–593. doi: 10.1016/j.molcel.2013.07.025.

Wang, S., Geng, Z., Zhang, H., She, Z., and Dong, Y. (2021a). The *Pseudomonas aeruginosa* PAAR2 cluster encodes a putative VRR-NUC domain-containing effector. *The FEBS Journal* 288, 5755–5767. doi: 10.1111/FEBS.15870.

Wang, T., Du, X., Ji, L., Han, Y., Dang, J., Wen, J., et al. (2021b). *Pseudomonas aeruginosa* T6SS-mediated molybdate transport contributes to bacterial competition during anaerobiosis. *Cell Reports* 35, 108957. doi: 10.1016/J.CELREP.2021.108957.

Wang, T., Hu, Z., Du, X., Shi, Y., Dang, J., Lee, M., et al. (2020). A type VI secretion system delivers a cell wall amidase to target bacterial competitors. *Molecular Microbiology* 114, 308–321. doi: 10.1111/MMI.14513.

Wettstadt, S., Wood, T. E., Fecht, S., and Filloux, A. (2019). Delivery of the *Pseudomonas aeruginosa* phospholipase effectors PldA and PldB in a VgrG- And H2-T6SS-dependent manner. *Frontiers in Microbiology* 10, 1718. doi: 10.3389/FMICB.2019.01718.

Whitney, J. C., Beck, C. M., Goo, Y. A., Russell, A. B., Harding, B. N., de Leon, J. A., et al. (2014). Genetically distinct pathways guide effector export through the type VI secretion system. *Molecular Microbiology* 92, 529–542. doi: 10.1111/mmi.12571.

Wilderman, P. J., Vasil, A. I., Johnson, Z., and Vasil, M. L. (2001). Genetic and biochemical analyses of a eukaryotic-like phospholipase D of *Pseudomonas aeruginosa* suggest horizontal acquisition and a role for persistence in a chronic pulmonary infection model. *Molecular Microbiology* 39, 291–304. doi: 10.1046/J.1365-2958.2001.02282.X.

Wood, T. E., Howard, S. A., Förster, A., Nolan, L. M., Manoli, E., Bullen, N. P., et al. (2019a). The *Pseudomonas aeruginosa* T6SS Delivers a Periplasmic Toxin that Disrupts Bacterial Cell Morphology. *Cell Reports* 29, 187-201.e7. doi: 10.1016/J.CELREP.2019.08.094.

Wood, T. E., Howard, S. A., Wettstadt, S., and Filloux, A. (2019b). PAAR proteins act as the ‘sorting hat’ of the type VI secretion system. *Microbiology (N Y)* 165, 1203. doi: 10.1099/MIC.0.000842.
